# Dual EMCV-IRES-integrated dengue virus can express an exogenous gene and cellular Mdm2 integration suppresses the dengue viral replication

**DOI:** 10.3389/fmicb.2025.1533062

**Published:** 2025-01-22

**Authors:** Tadahisa Teramoto

**Affiliations:** Department of Microbiology and Immunology, Georgetown University, Washington, DC, United States

**Keywords:** dengue virus, IRES, TP53, Mdm2, eGFP, Rluc, replication efficiency, GP160

## Abstract

Flaviviruses transmit through a wide range of vertebrate and arthropod hosts, while the other genera in *Flaviviridae* replicate in a limited set of vertebrate hosts. Flaviviruses possess a 5′ cap in their genome RNA for translation, while the other genera utilize their internal ribosome entry site (IRES) sequences instead of a 5′ cap. In this study, the translational modification to add an IRES sequence was examined. An IRES sequence derived from encephalomyocarditis (EMCV) was inserted into dengue virus serotype 2 (DENV2); a non-structural (NS) polyprotein was translated by IRES separately from 5′ cap-induced structural polyprotein translation. It was revealed that the IRES-integrated DENV2 is prevented from replicating in C6/36 mosquito cells, suggesting that the 5′ cap is an advantageous mechanism for flavivirus translation in invertebrate species. I further created dual IRES-integrated DENV2, in which a non-viral gene can be expressed by the flanking IRESs. The insertion of eGFP fluorescently visualized the virus spread. The renilla luciferase (Rluc) integration enabled the viral replication quantification. It was also revealed that a cellular gene, Mdm2, which antagonizes tumor suppressor protein p53 (TP53), could terminate the viral replication in BHK21 cells. Thus, the modifications of the DENV genome with IRES and the subsequent foreign gene could be utilized for controlling viral replications.

## Introduction

The genus *Flavivirus* belongs to the family *Flaviviridae,* which also includes the genera *Hepacivirus* and *Pestivirus*. The family *Flaviviridae* are enveloped viruses that have single-stranded, positive sense RNA genomes and that encode single polyproteins composed of N-terminal structural proteins and C-terminal NS proteins. Flaviviruses have evolved to replicate in both vertebrates (mostly mammals and birds) and hematophagous arthropods (typically mosquitoes and ticks). Humans are occasional hosts for zoonotic infections of many flaviviruses, among which dengue virus serotypes 1–4 (DENV1–4) have established a human–mosquito transmission cycle. DENV infections can cause febrile illnesses occasionally accompanied by fatal complications, such as liver failure and coagulation disorders. They are increasing and expanding in their endemic and epidemic areas due to human populations growing in areas inhabited by vector mosquitoes. In part, the severe dengue symptoms are induced by an antibody-dependent enhancement (ADE) mechanism, which facilitates the second infection of DENV in entering cells, utilizing humoral suboptimal antibodies produced during the first infection by a different DENV serotype. The ADE causes difficulties in developing a DENV vaccine. The currently approved “Dengvaxia” was reported to have a risk of enhancing DENV symptoms via ADE (Halstead, 2017) and therefore is limited in its use. Therapeutic drugs against flavivirus infections are also long-awaited, with no specific medicine currently available.

Flaviviruses utilize the 5′ cap in their RNA genomes for their polyprotein translations. 5′ cap translation starts by binding cellular translation initiation factors (eIFs), which recruit the ribosome, a central player in the cellular mRNA translation machinery. On the other hand, hepaciviruses and pestiviruses lack a 5′ cap but instead possess internal ribosome entry site (IRES) sequences, which can also recruit ribosomes. 5′ cap is a universal molecular signal equipped by all mRNAs in eukaryotes, while IRESs are present in fewer (10–15%) mRNAs (Spriggs et al., 2008). IRES sequences have evolved variously in species and their cell types to respond to specific biological signals, such as mitosis and apoptosis (Spriggs et al., 2008; Cobbold et al., 2008; Braunstein et al., 2007). In contrast, 5′ cap is a common system among species and is useful for flaviviruses in replicating among various host animals and vector insects.

After the flavivirus polyproteins are translated, three structural proteins: capsid (C), precursor membrane (prM), and envelope (E), and seven NS proteins: NS1, 2A, 2B, 3, 4A, 4B, and 5 are cleaved by viral protease (NS2B-activated NS3 protease: PRO) or cellular signal peptidase (signalase) residing at the endoplasmic reticulum (ER). Flaviviral prM, E, NS2A, 2B, 4A, and 4B proteins contain transmembrane (TM) domains that play an essential role in anchoring these proteins to the ER. It was reported that TM domains in prM and E are crucial for making viral particles with the ER membrane (Op De Beeck et al., 2003). TM domains in NS proteins are also vital for making the replication compartment of the ER membrane, where the viral 5′ cap RNA is replicated by the NS3 and NS5 enzyme complexes. NS3 contains RNA triphosphatase and helicase (HEL), while NS5 has guanylyltransferase, methyltransferase (MT), and polymerase (POL). These enzymes collaborate for viral genome replication, from synthesizing negative-strand RNAs to completing 5′ cap positive-strand RNAs.

I have utilized IRES sequences and created reporter replicons to measure RNA replication efficiency (Teramoto et al., 2014; Teramoto, 2023). IRES sequences were used to modify the replicon RNA translation after removing the viral structural genes. In this study, I further created reporter viruses, the artificial DENV2 co-expressing non-viral genes, by inserting dual IRES sequences. A Rluc- or eGFP-reporter gene was integrated into the DENV2 RNA genome. These artificial viruses replicated, infected, and caused cytotoxicity in BHK21 mammalian cells in a slower replication manner compared with wild-type (WT) DENV2. It was also revealed that this IRES translation mechanism is not sufficient for replication in C6/36 mosquito cells. It was further found that the integration of a cellular gene, such as mouse double minute 2 (Mdm2) oncoprotein, which can antagonize tumor suppressor protein P53 (Tp53), suppressed DENV2 replication in BHK21 cells. Tp53 has been variously reported for its effects on viral replication, although it was unknown if Mdm2 affected viral replication.

Thus, the IRES-integrated artificial DENV2 that I created is useful for analyzing the roles and effects of cellular proteins on flavivirus replication.

## Results

### IRES translation for NS proteins enables DENV2 replication but requires a hydrophobic amino acid region of C-terminal E prior to NS1

**Figure 1:**
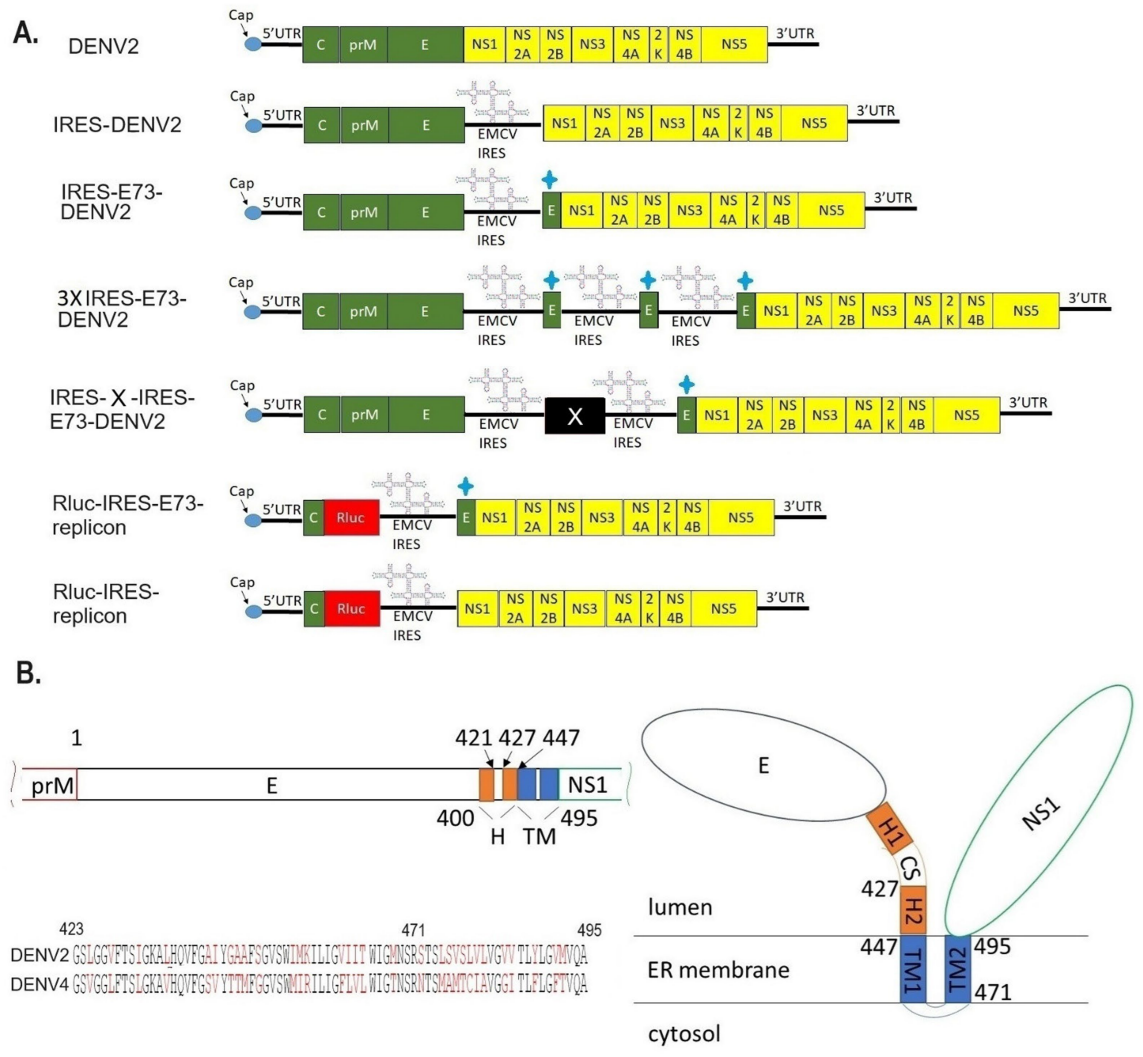
Designs of viral/replicon RNA genomes integrated with EMCV-IRES and/or the C-terminal E regions. **(A)** *In vitro* constructed RNAs. DENV2: WT DENV2 genome (top figure), IRES-DENV2: EMCV-derived IRES sequence was inserted between structural protein E, in which a stop codon was added at the C-terminus, and NS1, in which a start codon Met was added at the N-terminus (second figure). IRES-E73-DENV2: The repeated C-terminal E 73 amino acids were additionally inserted between Met and the first amino acid Asp in NS1 (third figure). 3XIRES-E73-DENV2: Triplicated IRES-E73 was inserted between structural protein E and NS1 (fourth figure). IRES-X-IRES-E73-DENV2: IRES-X-IRES-E73 was inserted at the boundary between E and NS1. X can be any exogenous gene. (fifth figure). Rluc-IRES-E73-replicon: The whole structural proteins except N-terminal C, which is necessary for 5′-3′ UTR circularization, were replaced with Rluc-IRES-E73 (sixth figure). Rluc-IRES-replicon: The whole structural proteins except N-terminal C were replaced with Rluc-IRES (seventh figure). + sign means the 73 a.a. from C-terminal E protein. **(B)** Theorized structure and sequences of C-terminal E. C-terminal E consists of H and TM domains (left top). TM domains are expected to anchor to the ER membrane (right). The alignments of E73 between DENV2 and DENV4 were compared (left bottom). The black letters are identical amino acids, while the red letters are the ones that are different between these serotypes. E73 (423–495 a.a.) and E25 (471–495 a.a.) are included. E25 has 48% identical a.a. between the two serotypes, while the rest of the a.a. in E73 is 63% identical.

**Figure 2:**
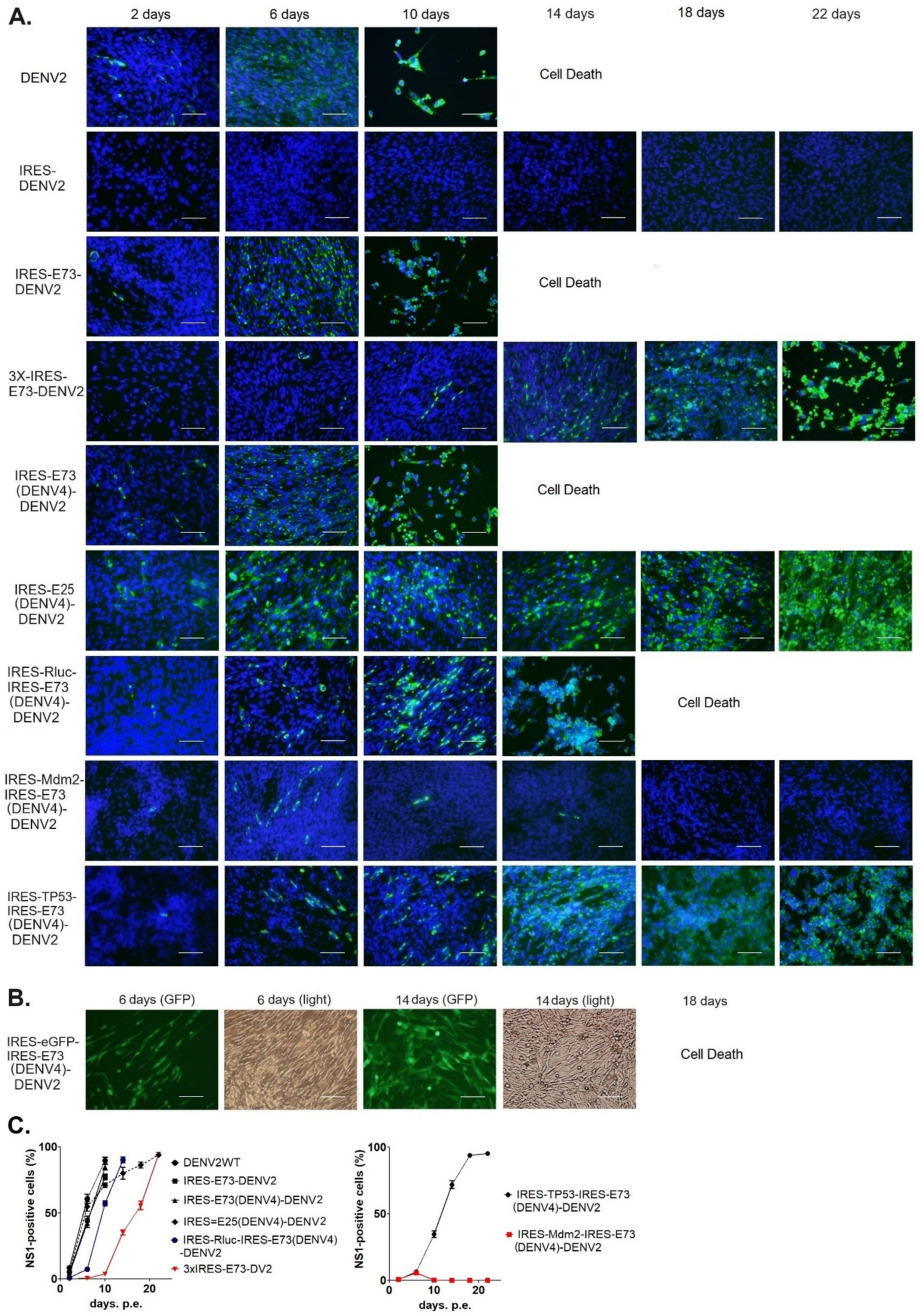
Immunofluorescence assay to monitor viral replications and expansions after electroporating into BHK21 cells. **(A)** Viral replications in the cells were monitored by immunofluorescence staining against DENV NS1 (IFA-NS1). FITC (green fluorescent)-labeled secondary antibody was used to detect the primary anti-NS1 antibody. IFA-NS1 (green fluorescent) and DAPI nuclei-blue fluorescent colors were merged to clarify the cellular localization of the viral NS1 protein. The scale bar represents 50 μm. **(B)** eGFP expressions (GFP) were visualized under a fluorescent microscope compared with cell shapes under light view. The scale bar represents 50 μm. **(C)** The experiments were performed in triplicate. NS1-positive cells were counted in six different microscopic views in each cell culture flask in three replicated experiments, using ImageJ software (NIH). The rates of NS1-positive cells were compared (means ± SEMs).

**Figure 3:**
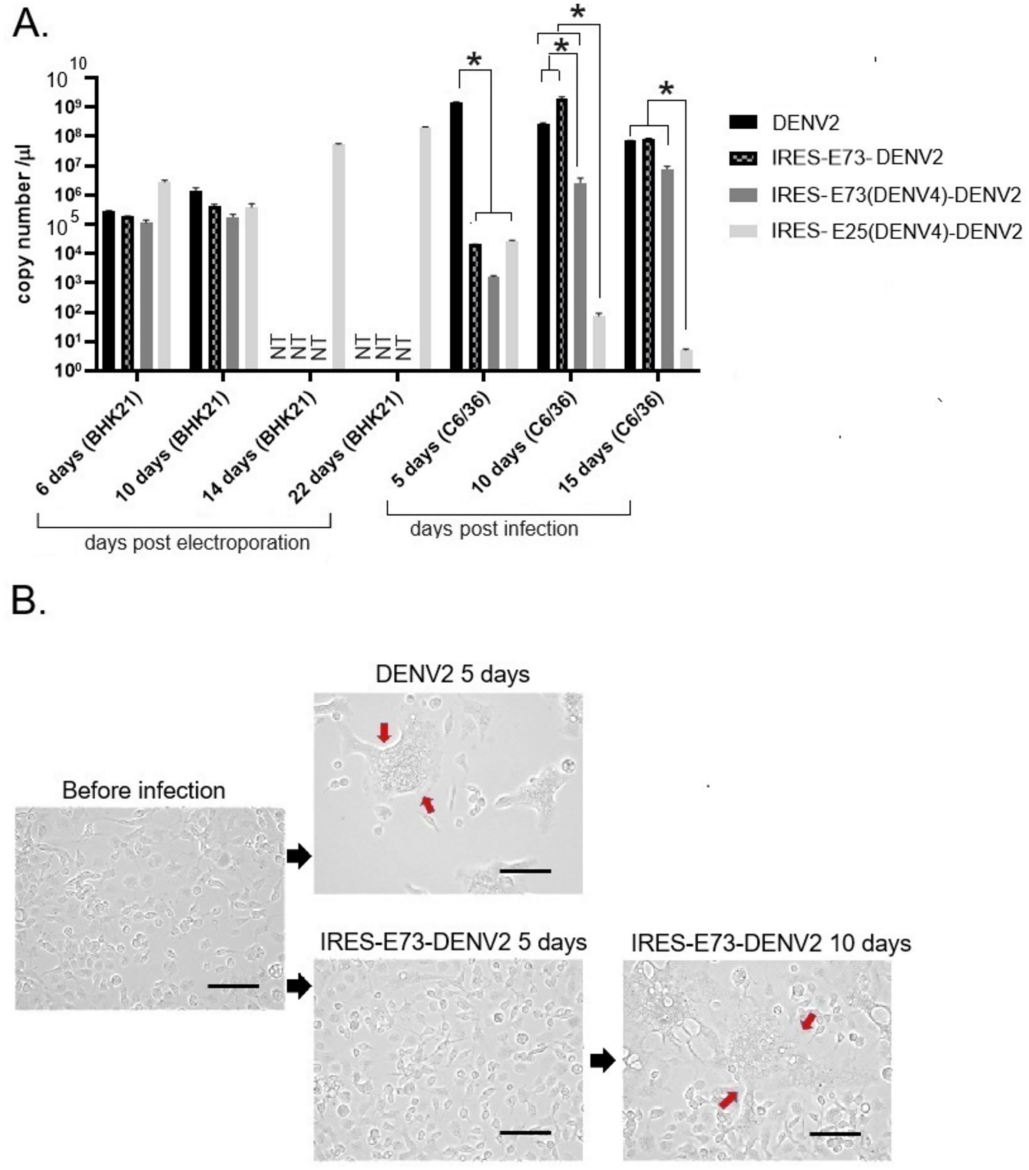
Viral RNA copy numbers and C6/36 cell morphological changes after DENV2 or IRES-E73-DENV2 infection. **(A)** Viral RNA copy numbers measured by RT-qPCR. RNAs were extracted from culture supernatants collected after electroporation into BHK21 cells or infection into C6/36 cells. NT (not tested) means that the cell culture was not continued due to cell death. Error bars show ranges between ±SEMs. Asterisk (*) means that *p* < 0.01 was confirmed by one-way ANOVA with Bonferroni post-test. **(B)** C6/36 cellular syncytia formation induced by DENV2 or IRES-E73-DENV2 infection. The large cells containing multiple nuclei (syncytia change) are indicated by red arrows. The scale bar represents 50 μm.

I designed and created EMCV-IRES-inserted DENV2 RNA, in which the IRES sequence translates NS polyprotein (NS1–NS5) separately from the 5′ cap-translated structural polyprotein (C-prM-E) (Figure 1A). The IRES-integrated DENV2 cDNAs were constructed through yeast recombination and were used for synthesizing each full-length RNA by *in vitro* transcription reaction with a cap analog. The *in vitro* synthesized RNAs were electroporated into BHK21 cells. I compared the effect of the presence or absence of C-terminal 73 amino acids (a.a.) of E (E73) since it was reported that hydrophobic residues at C-terminal E were necessary for the production of glycosylated NS1 (Falgout et al., 1989). There were also reports that C-terminal E contains two TM helices (TM1 and TM2) that anchor to the ER membrane and are necessary for virus assembly in DENV as well as in tick-borne encephalitis virus (Allison et al., 1999; Zhang et al., 2003) (Figure 1B). I inserted the C-terminal E covering TM1 and TM2 prior to NS1 under IRES-initiated translation (IRES-E73-DENV2) or did not include E73 (IRES-DENV2). The viral RNA replication and the viral expansion were monitored by immunofluorescence staining against NS1 (IFA-NS1). The IRES-E73-DENV2 showed initial replication at 2 days post-electroporation (p.e.) in a similar manner to wild-type (WT) DENV2 (Figure 2A). WT DENV2 and IRES-E73-DENV2 were quickly expanded through 6 days p.e. and induced cell death broadly at 10 days p.e., making it difficult to continue BHK21 cell cultures through later time points (Figures 2A,C). Viral copy numbers from supernatants of IRES-E73-DENV2 increased in BHK21 cells in similar amounts to WT DENV2 (Figure 3A). In contrast, IRES-DENV2 (without E73) did not show replication at any time points (Figure 2A). The results suggest that the preceding C-terminal E region prior to NS1 is necessary for IRES-DENV2 replication.

### Infectious viruses are produced from the 3x IRES-E73-integrated DENV2, despite slower replication compared to the 1x IRES-E73-integrated DENV2

When I constructed IRES-E73-DENV2 cDNA using the yeast recombination method, 3x IRES-E73-DENV2 was also made (Figure 1A). It is thought that the same sequences (~30 sequences) on both the 5′ and 3′ sides of the IRES-E73 fragment caused multiple recombinations. I tested if this 3x IRES-E73-DENV2 RNA was also replicable and infectious. After electroporating the 5′ cap RNA into BHK21 cells, IFA-NS1 showed the appearance of NS1-positive cells delayed at 6 days p.e. but gradually spread to the rest of cells and caused broad cell death (~22 days p.e.) (Figures 2A,C).

### IRES-integrated reporter replicon showed that E73 is necessary for RNA replication by NS polyproteins

**Figure 4:**
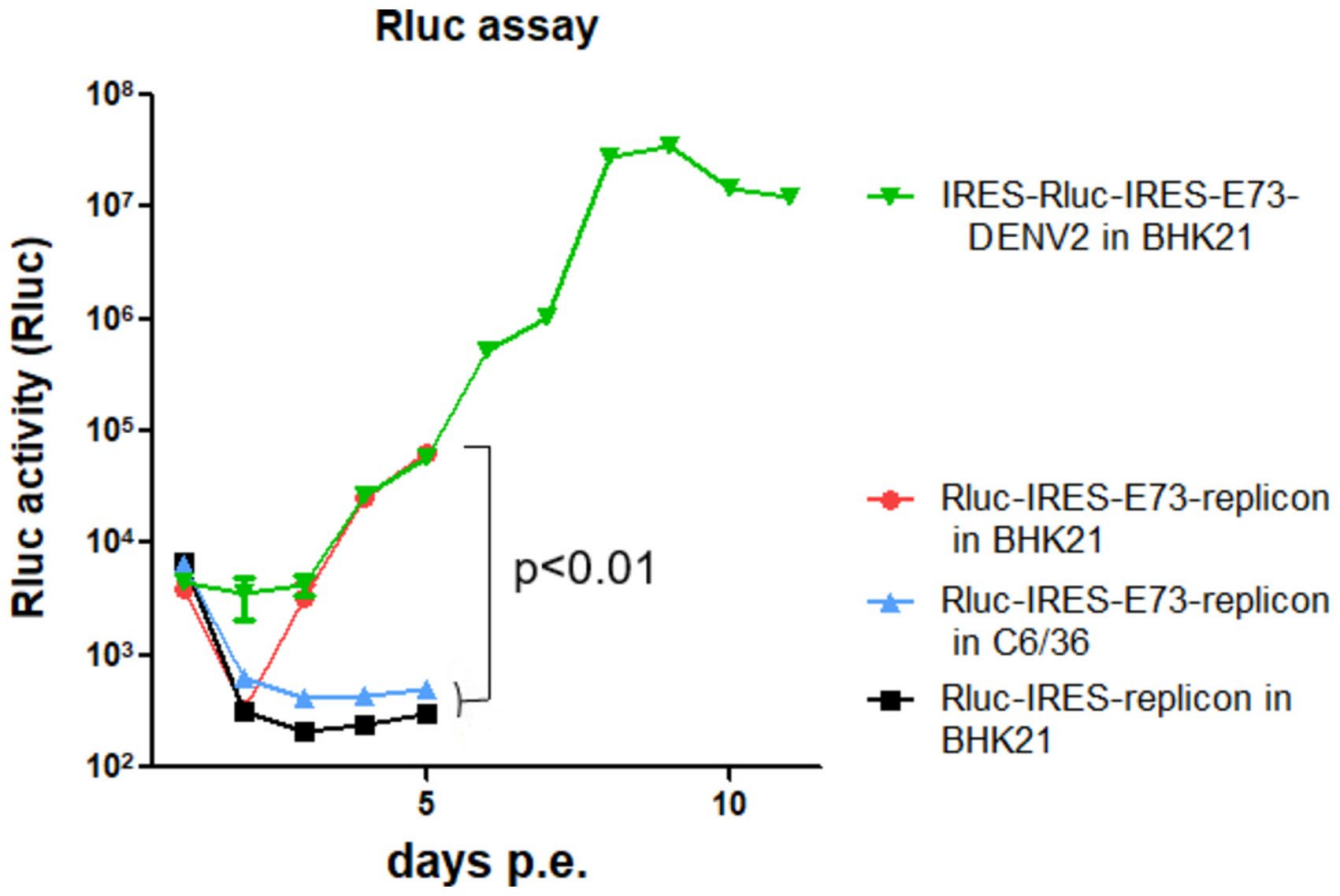
Rluc assays of reporter replicons (NS1–NS5) or reporter-DENV2. Rluc-IRES-E73-replicon was either electroporated into BHK21 or C6/36 cells, compared with Rluc-IRES electroporated into BHK21 cells. IRES-Rluc-IRES-E73(DENV4)-DENV2 was also electroporated into BHK21 cells. All RNAs showed similar Rluc activities at 4 h p.e. (the leftmost time points in the figure). These are the results of the 5′ cap-led translation of the input RNAs. There were no differences in Rluc activities, indicating that the transfection efficiencies and initial translation for Rluc protein induced by 5′ cap were similar among samples. All Rluc activities were reduced at 24 h p.e. by the natural decay of the input RNAs. After that, Rluc activities of replicon with E73 increased, while those without E73 did not. *p*-value of <0.01 was confirmed by one-way ANOVA with Bonferroni post-test.

I constructed *Renilla* luciferase (Rluc)-contained DENV2-NS polyprotein replicon RNA to measure RNA replication efficiency in the presence or absence of E73, prior to NS1 (Figure 1A). In the replicon RNAs, Rluc is translated by the 5′ cap, while NS proteins with or without E73 are translated by the EMCV-IRES. After each *in vitro* transcribed 5′ cap RNA was electroporated into BHK21 cells, Rluc activities were periodically measured. The presence of E73 led to a gradual increase in Rluc activity, while the absence of E73 did not (Figure 4), indicating that E73 is necessary for the RNA replication processes by NS proteins.

### IRES sequence functioned less and was removed from the viral genome during replication in C6/36 mosquito cells

**Figure 5:**
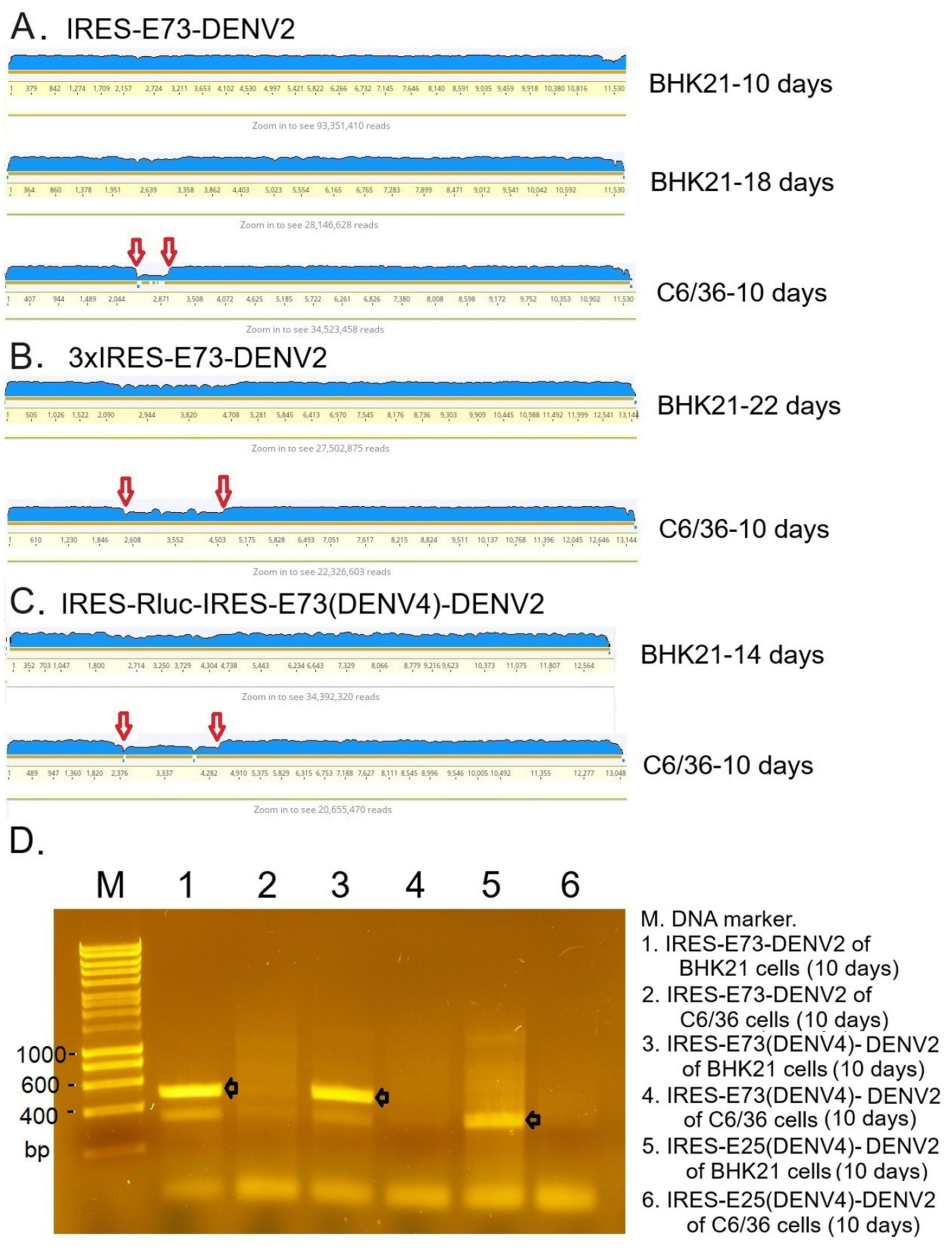
RNA-seq analysis mapping raw sequence data to the reference genome **(A–C)** and RT-PCR **(D)**. **(A)** IRES-E73-DENV2 viruses were analyzed in indicated cells on indicated days. **(B)** 3xIRES-E73-DENV2 viruses were analyzed in indicated cells on indicated days. **(C)** IRES-Rluc-IRES-E73(DENV4)-DENV2 viruses were analyzed in indicated cells on indicated days. The blue-colored layer corresponds to the number of fragments matching the reference genome. IRES-flanking regions, shown between the two red arrows, reduced after infecting C6/36 cells. **(D)** RNA was extracted from the indicated samples, and RT-PCR was performed to amplify IRES-E73 or IRES-E25 regions using primer pairs annealing to the IRES (forward) and NS1 (reverse) sequences. PCR elongation time was set to 20 s (the expected fragments, shown with black arrows, are ~500 bp for IRES-E75- and ~350 bp for IRES-E25-contained viral RNA, respectively). The PCR band detections were confirmed in all triplicate experiments.

**Figure 6:**
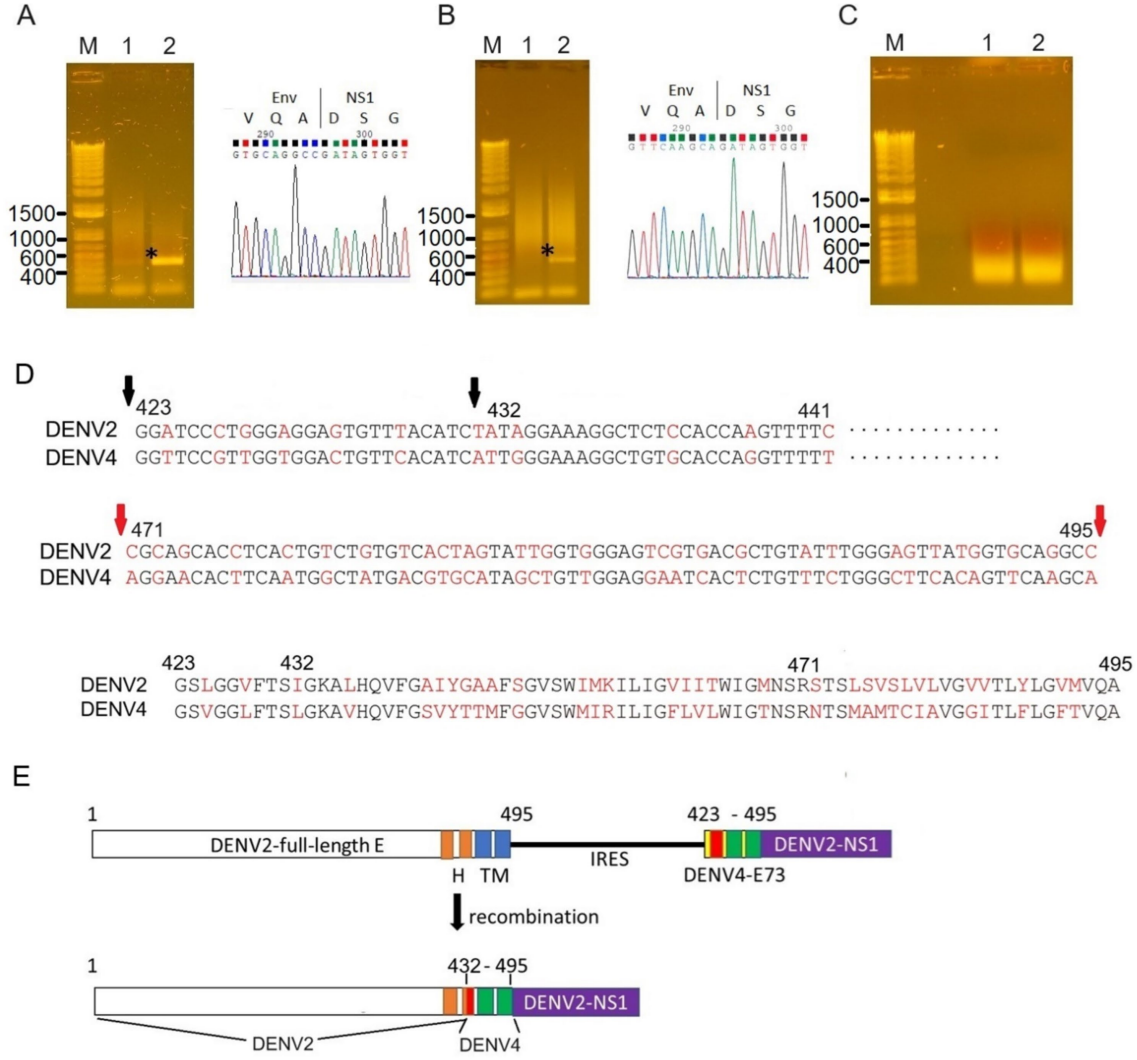
Sequence analyses of the recombined E-NS1 regions. RT-PCR was performed to amplify the potential recombined RNA between E and NS using primers corresponding to E (prior to E73, forward) and NS1 (reverse). Wild-E-NS1 length amplified by these primers was ~450 bp (shown as an asterisk). PCR elongation time was set to 15 s to avoid amplifying IRES-E73-contained viral RNAs (~1.3 kb). **(A)** The PCR band was detected from the supernatant of C6/36 cells (lane 2) but not from BHK21 cells (lane 1). The Sanger sequencing of this fragment corresponds to the wild-type sequence of E-NS1 (right picture). **(B)** The recombined fragment was detected from IRES-E73(DENV4)-DENV2 after infecting C6/36 cells. The E sequence was derived from DENV4 (right picture). **(C)** RT-PCR was not successful from IRES-E25(DENV4)-DENV2, both samples were from BHK21 cells and C6/36 cells. **(D)** DENV4-recombined E was analyzed by Sanger sequencing. C-terminal E maintained DENV2-derived nucleotides up to 432 a.a. (second black arrow, top), while the downstream region was replaced with the DENV4 sequence from E73. The different nucleotides between the DENV2 and DENV4 sequences are highlighted in red. C-terminal E25 nucleotides were more varied (shown between the flanking red arrows, middle). E75 a.a. is compared between DENV2 and DENV4 (bottom). Full sequence data are available in Supplementary material. **(E)** Recombined E genome made chimeric E between DENV2 and DENV4. The input RNA (top) was altered to chimeric E (bottom), which consists of 66 amino acids from DENV4 and at the C-terminus of E from DENV2. H: helical domain and TM: transmembrane domain. TM1 and TM2 were replaced with DENV4 sequences, while the H2 domain is composed of N-terminal DENV2 and C-terminal DENV4.

I tested if IRES translation functions in C6/36 mosquito cells. C6/36 cells were infected with IRES-E73-DENV2, which was collected from the supernatant in the electroporated BHK21 cells at 10 days p.e. As a control, the supernatant of wild-type (WT) DENV2-electroporated BHK21 cells at 10 days p.e. was used for infecting C6/36 cells. The copy numbers of the IRES-E73-DENV2 (~10^4^/μl) were significantly lower at 5 days post-infection (p.i.) compared to WT DENV2 (~10^8^/μl) but reached ~10^8^/μl at 10 days p.i. (Figure 3A). WT DENV2 induced the syncytial cytotoxic change in C6/36 cells within 5 days p.i., while IRES-E73-DENV2 delayed the morphological change to 10 days p.i. (Figure 3B). The syncytial formation and viral copy numbers in C6/36 cells were correlated in both WT and IRES-E73-integrated DENV2 replications. RNA-seq analyses were performed on the recovered viruses from BHK21 cells (10–22 days p.e.) and C6/36 cells (10 days p.i.) to analyze the viral sequences. The data showed that large numbers of RNA sequencing fragments corresponding to IRES sequences declined in IRES-E73-DENV2 after replicating in C6/36 cells at 10 days p.i. (200–400 reads, flanked regions shown by red arrows in Figure 5A) compared with those replicating in BHK21 cells at 18 days p.e. (100,000–200,000 reads), suggesting that the major virus population in C6/36 cells lost the IRES-integrated sequence. RT-PCR showed that the IRES was detected from collected viruses from BHK21 cell supernatants but not from C6/36 cells (Figure 5D). Furthermore, the WT length of E-NS1 was amplified from C6/36 cell supernatants but not from BHK21 ones (Figures 6A,B). The results confirmed that the viral genomes lost the IRES sequences after replicating in C6/36 cells and reconstituted the WT E-NS1 sequence. Sanger sequencing from the extracted RNA confirmed the lack of IRES sequences at the boundaries between the reconstituted E-NS1 sequences (Figure 6A,B). I tested the replication efficiency of the Rluc-IRES-E73-replicon RNA in C6/36 cells. It was revealed that the Rluc activity of the replicon electroporated into C6/36 cells did not increase through the time course (Figure 4), indicating that the IRES-E73 replicon is unable to replicate in C6/36 cells. All these results suggest that EMCV-IRES does not effectively function (i.e., does not translate NS proteins), resulting in viral replication failing selectively in C6/36 cells.

### The chimeric E73 replacement with the DENV4 corresponding could similarly produce virus progenies that efficiently replicate in BHK21 cells

I tested if DENV2 E73 could be replaced with a corresponding in different serotypes to determine if E73 is required for sequence identity for supporting NS proteins in replicating the RNA. I replaced DENV2 E73 with DENV4 corresponding, which has the lowest homology (58%) with DENV2 among the DENV four serotypes (Figure 1B). I also explored whether the reduced identities between structural E and E73 could prevent recombination to remove the IRES sequence. The *in vitro* transcribed IRES-E73(DENV4)-DENV2 showed efficient replication and expansion in BHK21 cells, similar to IRES-E73(non-chimeric)-DENV2 (Figures 2A,C). It was also revealed that IRES-E73(DENV4)-DENV2 delayed replicating viral copies in C6/36 cells in a slower manner than the IRES-E73(non-chimeric)-DENV2, but eventually reached the same levels at 15 days p.i. (Figure 3A). Sequence analysis showed that the recovered viruses from C6/36 cells removed the IRES sequence and reconstituted the structural E protein (Figure 6D,6E; Supplementary Figure 1). The recovered viruses reconstituted WT (DENV2) structural protein “E” to a chimeric protein, in which C-terminal 66 a.a of DENV2 were replaced by DENV4 sequences (Figure 6E), indicating that the C-terminal structural protein E does not require serotype sequence identity for envelope formation and function. However, since these 66 a.a. cover both TM1 and TM2 (Figure 1B), a question remains whether the same serotype pairing of TM1 and TM2 is required. In this regard, I further tested if the chimeric replacement targeting only TM2 (E25) could successfully replicate.

### “IRES-E25(DENV4)-DENV2” lacked cytotoxicity in BHK21 cells and did not induce recombination in C6/36 cells

I created IRES-E25(DENV4)-DENV2 RNA, containing the predicted TM2 domain (25 a.a. length, E25) only at the C-terminus to test if it supports IRES-integrated DENV2 replication and does not require a pairing with the same serotypic TM1 (Figure 1B). The 5′ cap RNA was electroporated into BHK21 cells. The viral RNA showed efficient replication in IFA until 6 days p.e., comparable to WT DENV2. However, after that, the virus progenies delayed cytotoxicity, enabling cell cultures beyond 22 days p.e. (Figure 2A). Viral copy numbers of IRES-E25(DENV4)-DENV2 increased similarly to those in WT DENV2 as well as IRES-E73-DENV2 (Figure 3A) but the numbers further increased at later time points, suggesting that a less cytotoxic effect contributes to replicating more viruses. Furthermore, after infecting C6/36 cells, IRES-E25(DENV4)-DENV2 decreased its copy numbers time-dependently (Figure 3A). I tested to detect the reconstituted viral genome, and IRES removal was not found by RT-PCR (Figure 6C). These results indicate that the virus did not revert to the wild-type sequence and could not expand in C6/36 cells.

### IRES-X-IRES-E73(DENV4)-DENV2 RNA (X: inserted gene) enables virus replication and expansion

Since Triad (3x) IRES-E73-integrated DENV2 RNA could produce infectious virus progeny in BHK21 cells, I designed a dual (2x) IRES-flanking X(gene)-integrated DENV2 (Figure 1A). I inserted reporter genes, such as Rluc or eGFP, into the IRES-flanking region to create IRES-Rluc-IRES-E73(DENV4)-DENV2 and IRES-eGFP-IRES-E73(DENV4)-DENV2. The *in vitro* transcribed 5′ cap RNAs were made from the cDNA plasmids and were electroporated into BHK21 cells. The viral replications and expansions were monitored by IFA-NS1 (Figure 2A) and by detecting eGFP (Figure 2B) under fluorescent microscopy. The viral expansion detected in IFA-NS1 showed that these dual IRES-integrated viruses spread faster in infecting all cells and inducing total cell death (~14 days p.e.) than 3x IRES-integrated DENV2 (~22 days p.e.), but slower than single (1x) IRES-E73-integrated DENV2 (~10 days p.e.). Rluc activities in the electroporated BHK21 cells increased time-dependently and reached the maximum level (>10^7^) on 10 days p.e. (Figure 4), correlating with the viral expansion shown by IFA-NS1 (Figure 2A). After infecting C6/36, IRES-Rluc-IRES-E73(DENV4)-DENV2 lost the IRES-flanking region, although the rate of the IRES-flanking site removal in the viral population was lower compared to IRES-E73-DENV2 (Figure 5C versus A). It is considered that the distance and less identical nucleotides between the structural protein E (DENV2) and E73 (DENV4) prior to NS1 suppress the recombination to reconstitute the E-NS1 sequence in C6/36 cells.

### The potential to regulate virus replication by integrating an exogenous gene into viral genomes; IRES-Mdm2-IRES-E73-DENV2 could suppress DENV2 replication

**Figure 7:**
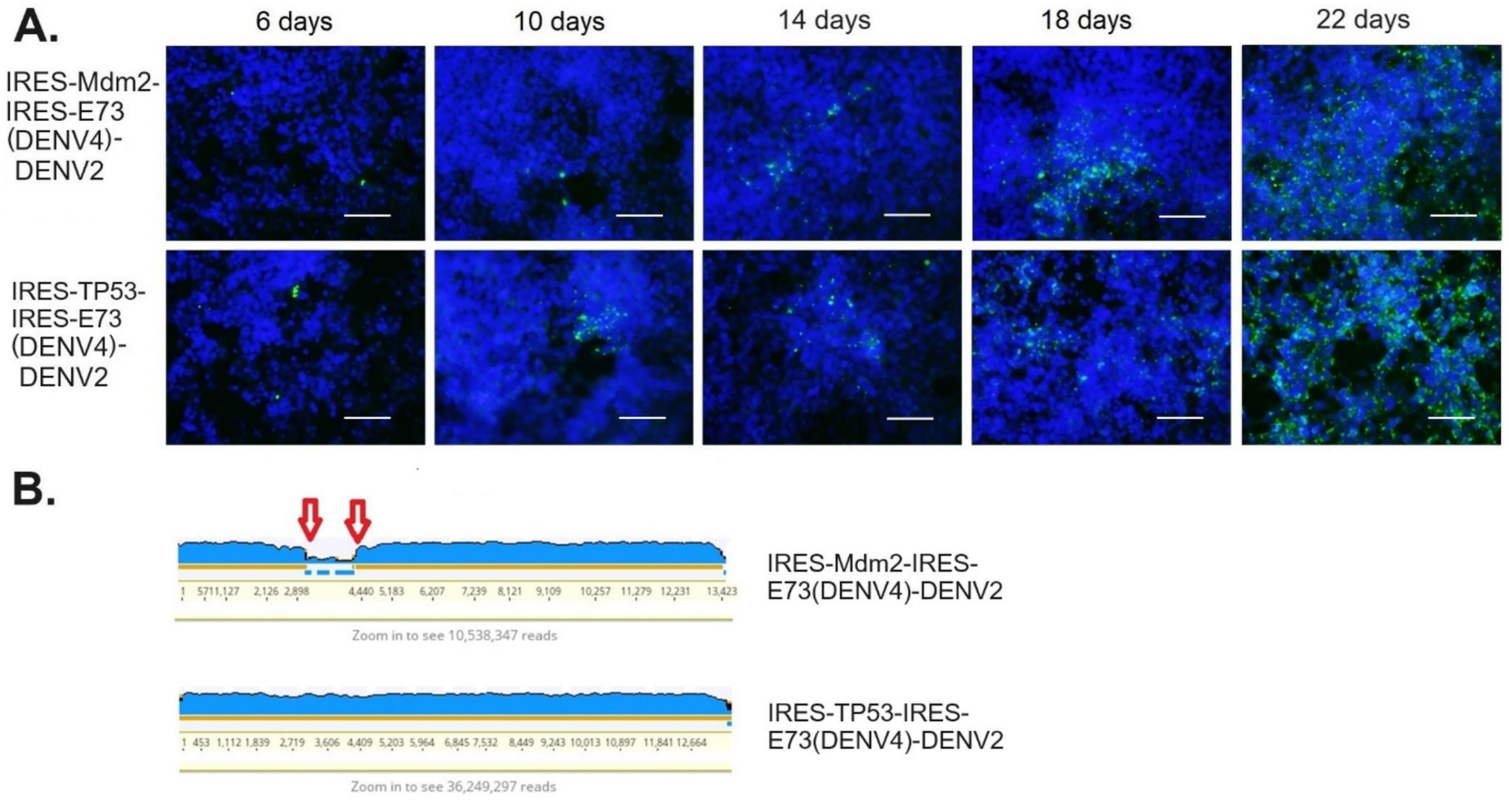
Mdm2- or TP53-integrated viral replications and expansions in HEK293 cells. **(A)** The viral RNAs were electroporated into HEK cells. Viral replications in the cells were monitored by IFA-NS1, coupled with FITC (green fluorescent)-labeled secondary antibody under DAPI nuclei-blue fluorescent staining. The scale bar represents 50 μm. **(B)** RNA-seq analysis of the supernatants at later time points (24 days p.e.) showed that IRES-Mdm2-integrated DENV2 reduced the IRES-Mdm2-IRES-E73 region (flanked by red arrows, top), while IRES-TP53-integrated DENV2 maintained the entire artificial viral sequence (bottom).

I also inserted cellular genes, such as TP53 or Mdm2, into IRES-flanking X (Figure 1A). It is known that the oncogene Mdm2 antagonizes the tumor suppressor TP53, which is an essential inducer of cell cycle arrest, senescence, and apoptosis, and moreover, has been reported to be involved in many viruses’ life cycles (reviewed in Aloni-Grinstein et al., 2018). Some viruses activate TP53 for their efficient replication, while others inactivate TP53. In flaviviruses, it was reported that the capsid protein in the West Nile virus (WNV) induces TP53-mediated apoptosis by intervening in the interaction between TP53 and Mdm2 (Yang et al., 2008). Zika virus (ZIKV) was also reported to elicit TP53 activation, which causes apoptosis in neuronal progenitor cells in an embryo, resulting in microcephaly in the infected fetus (Ghouzzi et al., 2016). Thus, programmed cell death could be induced via flavivirus-mediated TP53 activation, although it remains unknown if TP53 involvement is necessary for the flavivirus life cycle. In this study, I found that IRES-Mdm2-IRES-E73-DENV2 suppressed and ceased DENV replication until ~14 days p.e. In contrast, IRES-TP53-IRES-E73 replicated and expanded to the whole cell until ~14 days p.e. (Figure 2A). These results indicate that cellular Mdm2 affects DENV replication and has an anti-flavivirus function. The effect of Mdm2 was also tested in different cell lines, such as human embryonic kidney (HEK) 293 cells, in which WT-TP53 exists but its function is mediated by the adenovirus E1A and E1B oncoproteins (Debbas and White, 1993). It was revealed that IRES-Mdm2-IRES-E73-DENV2 could replicate and expand to the entire HEK293 cell in a similar manner to IRES-TP53-IRES-E73-DENV2 (Figure 7A). However, RNA-seq analysis showed that the IRES-flanking Mdm2 region was removed from replicating viruses (Figure 7B). In contrast, IRES-flanking TP53 was maintained in replicating viruses (Figure 7B). These results clearly showed that Mdm2 has an anti-flavivirus function and that it was thus necessary for it to be removed from the viral genome.

### The replacement of DENV E protein with human immunodeficiency virus (HIV) Env (GP160) did not affect IRES-E73(DENV4)-NS polyprotein replication

**Figure 8:**
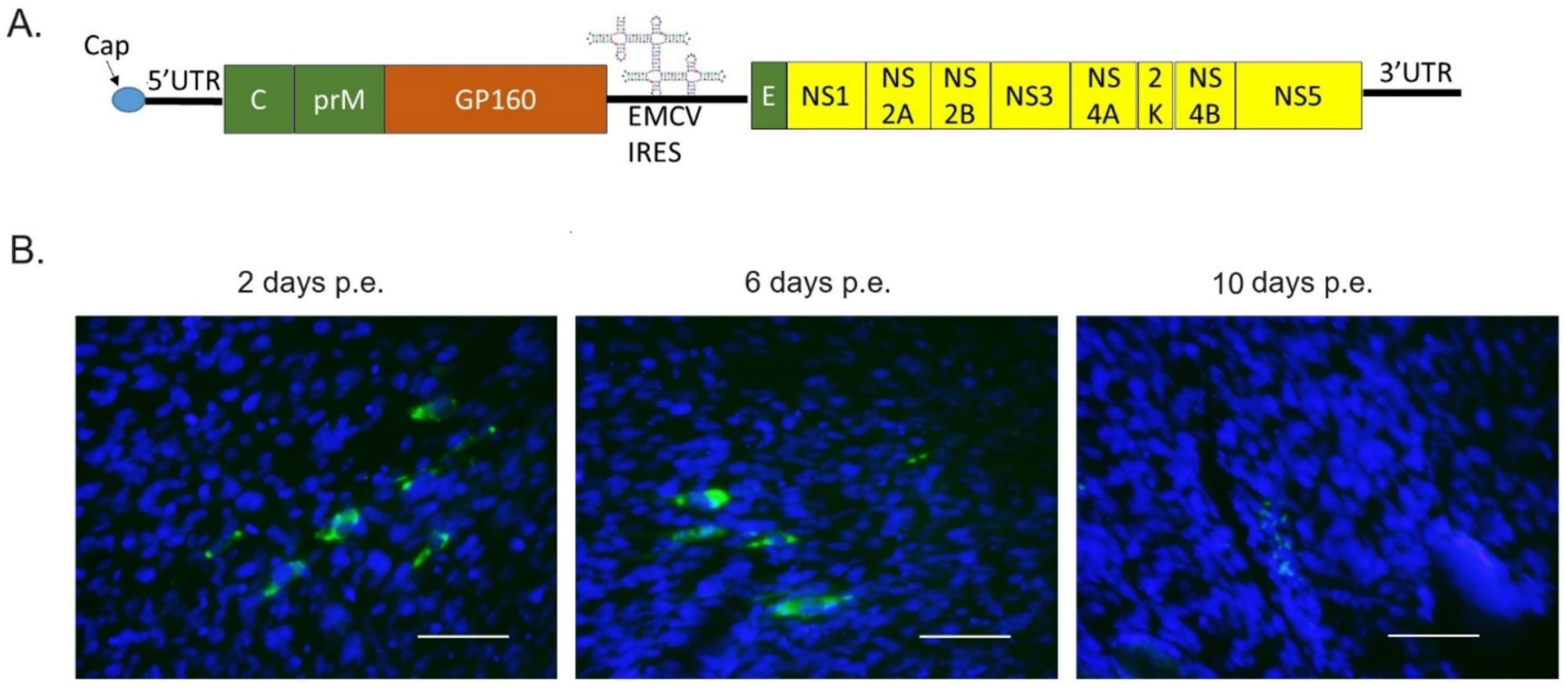
HIV GP160-replacing IRES-E73(DENV4)-DENV2 replicated in BHK21 cells. **(A)** The viral constructs were made to replace DENV structure protein E with HIV GP160. **(B)** The transcribed RNA was electroporated into BHK21 cells. Viral replications in the cells were monitored by IFA-NS1. FITC (green fluorescent)-labeled secondary antibody detects NS1 via the primary anti-NS1 antibody and is merged with DAPI nuclei-blue fluorescent staining. The scale bar represents 50 μm.

I further tested if the sequences of the structural E protein distantly affect RNA replication by the E73-NS polyprotein. I replaced the full DENV E with HIV env (GP160) (Figure 8A) and examined if viral replication and expansion in BHK21 cells would be affected. IFA-NS1 showed that the virus replicated at 2 days p.e. but diminished after 6 days p.e. (Figure 8B), indicating that GP160 does not intervene in the replication of RNA conducted by DENV E73 ~ NS proteins. The viral disappearance at later time points means that viral expansion did not occur in this RNA construct. This is possibly explained by a failure of particle formation and/or assembly by HIV GP160. BHK21 cells may also potentially lack a CD4 receptor, which is required for GP160 attachment and fusion to cells.

## Discussion

Flaviviruses in the *Flaviviridae* family and alphaviruses in the *Togaviridae* family transmit between vertebrates and arthropods, causing zoonotic infections in humans. Both viruses have 5′ cap single-stranded (+) sense RNA genomes. In alphaviruses, the alteration of translation initiation from 5′ cap to EMCV-IRES has suppressed viral replication selectively in mosquito cells (Volkova et al., 2008; Plante et al., 2011). In flaviviruses, it has been reported that EMCV-IRES insertion into the yellow fever virus (YFV) genome between C and prM suppressed viral replication in C6/36 cells (Wang et al., 2022). In this study, I revealed that EMCV-IRES insertion between E and NS1 in DENV2 also selectively suppressed viral replication in C6/36 cells, in which viral progenies evolved to reconstitute their RNA genomes by removing the IRES sequences (Figures 5–7). The reporter replicon RNA, coding NS polyprotein under EMCV-IRES translation initiation, could be replicated in BHK21 cells but not in C6/36 cells (Figure 4). These results indicate that EMCV-IRES does not efficiently promote translation in mosquito cells. It is also thought that the number of integrated IRES determines the time for completing translation, which eventually affects the replication cycle since each IRES requires a separate translation process that must bind eIFs and bring ribosomes for translating each coding protein. It is considered that a 5′ cap in the viral genomes enables flavivirus replication in a wide range of host and vector species in vertebrates and invertebrates.

Data also showed that the NS polyprotein required co-translation of the preceding hydrophobic amino acids at C-terminal E. The lack of preceding C-terminal E prior to NS1 does not produce the viral RNA in BHK21 cells, while E73, covering helices domain 2 (H2), TM1, and TM2 or E25, corresponding to only TM2 (Figure 1B) enabled the viral replication and expansion in BHK21 cells. These C-terminal E regions are replaceable with the corresponding from other serotypes, such as DENV4, which has the least a.a. identity with DENV2. These results indicate that the role of C-terminal E is based on its function but not on sequences that may interact with DENV proteins. The longer E73 provides full viral capacities comparable to WT DENV2, while the shorter E25 produces less cytotoxic viral progenies. The biological roles of H and TM domains were reported to serve for virus assembly, a process of packaging the synthesized RNA into the virus particle (Lin et al., 2011; Hsieh et al., 2010). Data showed that the viral expansion of IRES-E25-DENV2 in BHK21 cells was similar to WT DENV2 and did not suggest a major defect in viral replication, including assembly. It remains unknown how the upstream of E25, containing H2 and TM1 domains, is involved in cytotoxicity. The IRES-E25 insertion into DENV2 also did not induce viral genome recombination to remove the IRES sequence (Figure 6C). The virus progenies could not expand in C6/36 cells and disappeared (Figure 3A). Nucleotide sequence identities of E25 between DENV2 and DENV4 were lower (57%) than those of 5′ terminal E73 (77%), potentially preventing homologous recombination between structure protein E (DENV2) and E25 (DENV4) prior to NS1 (Figure 6D). Another possibility is a requirement for the same serotypic pairing of TM1 and TM2 for structure protein E to function. Even if the chimeric TM1/TM2 in structure protein E is made by recombination, this fused E may have lost replication competency and/or infectivity. Further analysis to compare chimeric structural E proteins made of the same or different serotype-derived TM1 and TM2 is necessary to verify this possibility.

NS1 is a N-glycosylated protein at N130 and N207 residues, both of which are conserved among flaviviruses, including all four DENV serotypes. N-glycosylation is an important modification of NS1 for its various functions and characteristics, such as secretion, stability, neurovirulence, and silencing immunity by interacting with “complement” components (Somnuke et al., 2011; Crabtree et al., 2005). It is known that the mutant DENV2 lacking these glycosylation sites in NS1 delayed and reduced the cytopathic effect (Crabtree et al., 2005) and attenuated virulence and pathogenesis (Fang et al., 2023). It was revealed that N-glycosylation of NS1 required 24 amino acid residues at the C-terminal E (Falgout et al., 1989). It is considered that the 24 amino acids direct NS1 to the ER since N-linked protein glycosylation occurs at the ER (reviewed in Aebi, 2013). In the given data, the lack of preceding NS1 did not replicate a virus progeny, indicating that co-translation of TM2 prior to NS1 is necessary for delivering NS1 to the ER, where NS1 and dsRNA co-localize in the viral replication compartment (Mackenzie et al., 1996). NS1 is required for flavivirus RNA synthesis by remodeling the ER structure for a flavivirus replication compartment (Ci et al., 2020; Youn et al., 2013). Moreover, since E-NS1 and NS1-NS2A boundaries are cleaved by cellular protease at the ER (Falgout et al., 1989; Falgout and Markoff, 1995), the lack of E25 also could affect completing E, NS1, and NS2A productions.

I created the dual IRES-integrated DENV2, which translates the viral NS gene as well as the non-viral additional gene independently of the 5′ cap-driven structural polyprotein. By integrating eGFP or Rluc into the dual IRES-E73-DENV2 RNA, virus replication in BHK21 cells was visualized or quantified. Furthermore, it was revealed that the cellular oncogene Mdm2 integration suppresses viral replication in BHK21 cells. Since Mdm2 plays an important role in antagonizing TP53, it is speculated that suppressing TP53 function via Mdm2 could affect DENV replication. It is known that DENV2 replication in BHK21 cells causes cell growth arrest at the G1 phase, followed by apoptosis, which could be inhibited by bcl-2 overexpression (Su et al., 2001). Elevation of TP53 protein amount by DENV1 infection was also shown in BHK21 cells (Nasirudeen et al., 2008). These results indicate that the pathway to TP53-dependent, mitochondria-mediated cell death by DENV infection is present in BHK21 cells. In contrast, HEK293 cells contain the integrated adenovirus genome fragment on chromosome 19 (Graham et al., 1977; Louis et al., 1997) and express the adenoviral E1A/E1B proteins, which interfere with the cell cycle and counteract apoptosis (Debbas and White, 1993). It has been shown that E1B-55 K has several activities that inhibit TP53 function (reviewed in Berk, 2005), suggesting that the function of TP53 is suppressed in HEK293 cells. Results indicate that TP53-integrated DENV2 replicated and expanded similarly compared to reporter-integrated DENV2 electroporated in BHK21 cells or HEK293 cells. Furthermore, the IRES-flanking TP53 region remained intact in the viruses replicating in HEK293 cells (Figure 7B, bottom). These results suggest that the role of TP53 in DENV replication is not essential in BHK21 and HEK21 cells. In contrast, Mdm2-integrated DENV2 ceased replication and disappeared from BHK21 cells. It also induced viral genome recombination, removing the IRES-flanking Mdm2 region in HEK293 cells (Figure 7B, top), clearly indicating that Mdm2 plays a role in suppressing DENV replication in these cells. Taken together, it seems that the anti-viral function of Mdm2 is not related to antagonizing the TP53 function. Mdm2 is known to have a TP53-independent function, e.g., as E3 ubiquitin ligase to degrade various proteins (reviewed in Bohlman and Manfredi, 2014). The antivirus effect of Mdm2 was reported, although the clear mechanism to prevent viral replication is not known (Yang et al., 2012). Mdm2 also has been reported to be expressed in many tumors, especially soft-tissue sarcomas and osteosarcomas (Kato et al., 2018). Since dual IRES-DENV2 could introduce Mdm2 expression in cells, there is a risk of transforming the cells into cancer cells. However, Mdm2 expression is conducted through viral replication and has anti-viral activity, resulting in ceasing the viral expansion at early time points; the risk of carcinogenesis in the infected cells thus seems minimal. Further analysis regarding the anti-viral activity of Mdm2 must be performed.

It is important to note that since IRES translation theoretically works with any exogenous gene in a given cell, the expression system itself is not questioned. However, the exogenous gene expression itself could be reduced or lost because the virus could recombine the genome or lose replication ability. TP53 is quite an important gene, whose effects on downstream molecules are regulated post-translationally by various antagonists such as Mdm2. Therefore, the lack of an effect of TP53 on DENV2 replication in this experiment is not conclusive of a lack of TP53 expression. Similarly, the suppressive effect of Mdm2 on DENV2 cannot be generalized. The effects of exogenous gene expression depend on the cell type, and further experimentation is required.

Successful cellular protein expression in the flavivirus genome was also reported in the Murray Valley encephalitis virus, in which beta interferon (IFN-β) and EMCV-IRES were inserted between C and prM (Frese et al., 2014). IFN-β was translated after the structure protein “C” by 5′ cap, while prM ~ NS5 polyprotein was translated by the inserted EMCV-IRES (Frese et al., 2014). It was shown that suppressed viral replication was observed in mouse embryo fibroblast cell cultures and mouse animal models although the virus replicated quite efficiently (Frese et al., 2014). In contrast, RNA constructs integrating IRES-flanking Mdm2 completely shut down viral replication. This enables the creation of artificial viruses that could have their replication and expansion controlled. Since structure proteins and NS proteins are translated differently in the construct, various patterns of chimeric viruses, e.g., structure proteins derived from different viral genera and families, could be made and tested for the mechanisms of viral life cycles under careful designs and attenuating strategies. The replacement of E in IRES-E73-DENV2 with HIV’s GP160 (HIV and DENV are both classified as Biosafety Level 2) showed that the initial replication conducted by DENV NS proteins was not prevented, but this “chimeric virus construct” perished. Therefore, this may be used as a vaccine strategy, although more clarity on the mechanisms around how the structural proteins and NS proteins in DENV collaborate is needed to prevent the risk of producing an undesirable or unexpected virus. It is also possible to exchange the IRES sequence for a different one, which would make species-specific, limited replicable flaviviruses that respond to specific cell types and species.

## Materials and methods

### Construction of full-length DENV2 cDNAs inserted by IRES, IRES-E73, IRES-E73(DENV4), IRES-E25(DENV4), or IRES-X-IRES-E73 as well as replicon cDNAs

The yeast/*E. coli* shuttle vector plasmid pRS424 containing the full-length DENV2 cDNAs (pRS424-FLDV2, gifts from Dr. Falgout, FDA) was used as a background genome for constructing artificial virus and replicon cDNAs. The inserted fragment cDNAs, EMCV-IRES, E73, E73 (DENV4), E25 (DENV4), eGFP, Rluc, Mdm2, or TP53 were individually amplified by PCR, including 5′ and 3′ adjacent homologous sequences (overlapping >30 nucleotides; primer details are described in Supplementary Table 1). The amplified PCR products were mixed with the linearized pRS424-FLDV2 lacking the replacing sequences (through 3,196–5,426 nt position by BstEII and XhoI) for homologous recombination in *Saccharomyces cerevisiae* YPH857 competent cells. The recombined full-length cDNA plasmid-contained *S. cerevisiae* was selected and amplified in tryptophan-lacked yeast media. Individual plasmids were extracted from the yeast cells and used for transforming *E. coli* stbl2 competent cells in ampicillin-contained LB media. The amplified plasmids were extracted from 100 mL of confluent media with *E. coli* cells.

### *In vitro* RNA transcription and electroporation into BHK21 or HEK293 mammalian cells

The created viral cDNA-contained plasmids were linearized at the viral 3′ end by digesting with SacI, BcgI, or XmaI, one of which was selected by the absence of internal cutting in the viral sequences. The linearized cDNAs were used as templates for *in vitro* transcription reactions to synthesize 5′ cap RNAs with SP6 RNA polymerase and m^7^GpppG cap analog (Polo et al., 1997; Adams et al., 2002). Each synthesized RNA (~3 μg) was electroporated into ~1 × 10^6^ BHK21 cells (American Type Culture Collection) or HEK293 cells (Bei Resources) using the Amaxa Nucleofector II system (Amaxa). The pulsed cells were immediately spread on a T-25 flask with DMEM medium (Gibco), supplemented with 5% fetal bovine serum (FBS), and were maintained at 37°C in a humid 5% CO2 incubator. The cells were split into a T-75 flask at 2 days p.e., which was followed by repeated splitting every 4 days using one-third of the trypsinized cells and fresh media (Teramoto et al., 2017).

### Immunofluorescence (IF) staining against NS1 for detecting WT or their genome-modified DENV2

The replications of the reproduced viruses in cell cultures were periodically monitored by IF staining against viral NS1. The acetone/methanol-fixed cells were incubated with PBS containing 7E11, a monoclonal anti-DENV NS1 antibody (1:200 dilution, gift from Dr. Falgout, FDA) for 4 h at room temperature. FITC-labeled goat anti-mouse IgG conjugate was used as a secondary antibody (1:100 dilution, SeraCare) to visualize viral NS1’s cytoplasmic localization under an epifluorescent microscope (Olympus IX-71), while nuclear staining was performed with DAPI (1:400 dilution, KPL). The rate of viral NS1-positive cells versus total cell numbers in more than six microscopic fields was counted under NIH ImageJ software and compared among samples of electroporated cells on different testing dates.

### Measurement of viral copy numbers (RT-qPCR)

WT or genome-modified DENV2s in the supernatants of cell cultures were extracted using a Quick-RNA™-Viral kit (Zymo Research). RT reaction was performed to make the viral cDNAs by ProtoScript II reverse transcriptase (NEB) with random primers and dNTPs (NEB) at 42°C for 2 h. The copy numbers of cDNAs were measured by qPCR with a primer pair amplifying NS1 region (Supplementary Table 1) and iTaq Universal SYBR green Supermix (Bio-Rad) in a Mic qPCR cycler (Bio Molecular Systems). The viral copy numbers were calculated from the average threshold cycle (Ct) values by comparing them against the standard amounts of DENV2 cDNAs.

### Infection of viruses collected from BHK21 cells with C6/36 mosquito cells

The collected supernatants of the electroporated BHK21 cells were used for infecting C6/36 cells (*Aedes albopictus* mosquito cells) at ~1,000 viral copies/cell (not MOI but copy numbers based on RT-qPCR). C6/36 cells were maintained in Schneider’s Insect media with 5% FBS and 25 mM HEPES buffer at 30°C before virus infection. After virus infection, cells were maintained in a DMEM medium with 5% FBS and 25 mM HEPES. The supernatants were collected every 5 days and replaced with fresh media for cell culture.

### Rluc reporter replicon assay

After restriction enzyme (e.g., SacI) digestion, the constructed cDNA plasmids at the 3′ termini, Rluc-contained replicons, or viral RNAs were *in vitro* transcribed with a cap analog under Sp6 polymerase reaction and were electroporated into BHK21 cells. The cells were cultured in six-well plates. Each well was lysed for protein extraction periodically with 250 μL of 1x Rluc lysis buffer (Promega). Rluc activities were measured in duplicated lysed samples (50 μL) using a Centro LB960 luminometer (Berthold Technologies) by injecting 50 μL of 1x Rluc substrate (Promega) with settings of 10-s reading and 2-s delay after substrate injection.

### RNA extraction from viral particles and RNA-seq analysis

Supernatants (~10 mL) were collected from BHK21, HEK293 or C6/36 cell cultures in T-75 flasks. Viral particles were precipitated by centrifugation at 15,000 *g* for 45 min in a 40% PEG8000 solution containing 10 mM Tris (pH 8.0), 120 mM NaCl, and 1 mM EDTA (Teramoto et al., 2017). The precipitates in the remaining 0.5 mL of solutions at the bottom of the tubes were thoroughly mixed with 1.5 mL of TRIzol-LS (Ambion) and 0.4 mL of chloroform. After the centrifugation of these mixed solutions at 12,000 g for 15 min, the upper layer that separated from TRIzol/chloroform (bottom layer) was used for ethanol precipitation to collect viral RNA. The pellet was washed with 70% ethanol, dried, and dissolved in Tris (10 mM, pH 8.0)-buffered DEPC-water. The RNA was selected with the RNA Clean & Concentrator™ kit (Zymo Research), containing DNase. Library preparation and RNA-seq sequencing were performed at Zymo Research Inc. (Irvine, CA), where each RNA sample (260 ng) was purified using the Zymo-Seq RiboFree Total RNA Library kit (Zymo Research). The purified, rRNA-depleted RNA samples were reverse-transcribed into cDNAs. The produced cDNAs were ligated with the P7 adaptor sequence at the 3′ end, followed by second-strand synthesis and P5 adaptor ligation at the opposite sites of the double-stranded DNA. After purification by DNA size (300–600 bp) with beads in the kit, index PCR was performed (initial denaturation at 95°C for 10 min, followed by 16 cycles of denaturation at 95°C for 30 s, annealing at 60°C for 30 s, and extension at 72°C for 60 s, in addition to a final extension at 72°C for 7 min). Successful library construction was confirmed with Agilent’s D1000 Screen Tape Assay on TapeStation. Libraries were sequenced on an Illumina NovaSeq to a sequence depth of >30 million read pairs (150 bp paired-end sequencing). Raw Fastq files created from RNA-seq reactions were used for alignment analysis of DENV sequences using Geneious Prime software (Biomatters). After trimming the 3′ end of R1 and R2 with the BBDuk plugin, the paired-end sequences were made and aligned to the reference sequence. The created contigs were analyzed for SNPs. Nucleotide alterations at more than 5% frequency were shown.

### Statistical analysis

Measurements of viral copy numbers, viral titers, and Rluc replicon assays were performed with duplicated samples, and the average numbers and 95% CI were calculated. Viral NS1-positive cell rates were calculated with six microscopic fields, and the average numbers and 95% CI were calculated. Variation measured by significant difference was assessed with one-way ANOVA with Bonferroni post-test using Prism (GraphPad software).

## Author’s note

I artificially created the translation-modified DENV2, in which viral NS genes as well as additional non-viral genes were translated by EMCV-IRES independently of the 5′ cap in the viral genome. The replication of eGFP or Rluc gene-integrated DENV2 could be traced with these markers in BHK21 and HEK293 mammalian cells, while both viruses were suppressed in C6/36 mosquito cells. Moreover, the integrated cellular Mdm2 gene suppressed viral replication in BHK21 cells, suggesting the potential creation of a replication-controllable virus by IRES insertion and cellular gene integration with the viral genome.

## Data Availability

The RNA-seq data as raw reads are available as Fastq files in the NCBI Short Read Archive (SRA) Data/Download Web page (The BioProject accession number, PRJNA1117057).
