# Supplementary material for: Dual EMCV-IRES-integrated dengue virus can express an exogenous gene and cellular Mdm2 integration suppresses the dengue viral replication
Source: Front Microbiol. 2025 Jan 22;16:1533062. doi: 10.3389/fmicb.2025.1533062 (PMC11794298; doi:10.3389/fmicb.2025.1533062)
Supplement: Supplementary file 1 [file Data_Sheet_1.docx]

The RNA-seq data as raw reads are available as Fastq files in the NCBI Short Read Archive (SRA) Data/Download Web page (The BioProject accession number, PRJNA1117057).

Supplement Table 1. PCR Primers used for the construction of full-length DENV2 cDNAs inserted by IRES, IRES-E73, IRES-E73(DENV4), IRES-E25(DENV4), IRES-X-IRES-E73, GP160(HIV)-IRES-E73(DENV4)-DENV2 as well as replicon cDNAs.

| PCR fragment | forward | reverse | DNA template |
| --- | --- | --- | --- |
| E-IRES | CATGTCAGCGGCCATAAAAGA (2970F) | ACGTTAGGGGGGGGGGAGGGAGAGGGGC  GGCCGCCTAGGCCTGCACCATAACTCCCA | pRS424-FLDV2 |
| E-IRES-NS1 | CGTGACGCTGTATTTGGGAGTTATGGTGC  AGGCCTAGGCGGCCGCCCCTCTCCCTCCC | AGTTCTTTGTTTTTCCAGCTCACAACGCAA  CCACTATCCATGGTATTATCGTGTTTTTC | EMCV-IRES |
| IRES-NS1-NS3 | GACGTGGTTTTCCTTTGAAAAACACGATAA  TACCATGGATAGTGGTTGCGTTGTGAGC | GGTTCTAGTCTTGACATACTC (5700R) | pRS424-FLDV2 |
| E-IRES-E73 | CGTGACGCTGTATTTGGGAGTTATGGTGC  AGGCCTAGGCGGCCGCCCCTCTCCCTCCC | TTCCTATAGATGTAAACACTCCTCCCAGG  GATCCCATGGTATTATCGTGTTTTTCAAA | EMCV-IRES |
| IRES-E73-NS3 | ACGTGGTTTTCCTTTGAAAAACACGATAA  TACCATGGGATCCCTGGGAGGAGTGTTT | GGTTCTAGTCTTGACATACTC (5700R) | pRS424-FLDV2 |
| E-IRES- E73(DENV4) | CGTGACGCTGTATTTGGGAGTTATGGTGC  AGGCCTAGGCGGCCGCCCCTCTCCCTCCC | TCCCAATGATGTGAACAGTCCACCAAC  GGAACCCATGGTATTATCGTGTTTTTC | IRES-DENV2 |
| IRES-E73(DENV4)-NS1 | GTGGTTTTCCTTTGAAAAACACGATAAT  ACCATGGGTTCCGTTGGTGGACTGTTC | GTTCTTTGTTTTTCCAGCTCACAACGCAAC  CACTATCTGCTTGAACTGTGAAGCCCAG | pRS424-FLDV4 |
| E73(DENV4)-NS1-NS3 | GGAATCACTCTGTTTCTGGGCTTCACAGTT  CAAGCAGATAGTGGTTGCGTTGTGAGC | GGTTCTAGTCTTGACATACTC (5700R) | pRS424-FLDV2 |
| E-IRES-Rluc | CGTGACGCTGTATTTGGGAGTTATGGTGC  AGGCCTAGGCGGCCGCCCCTCTCCCTCCC | GTTTGCGTTGCTCGGGGTCGTACACCTTG  GAAGCCATGGTATTATCGTGTTTTTCAAA | IRES-E73-DENV2 |
| IRES-Rluc-IRES | GACGTGGTTTTCCTTTGAAAAACACGATA  ATACCATGGCTTCCAAGGTGTACGACCCC | AACGTTAGGGGGGGGGGAGGGAGAGGGG  CGGCCGCCTACTGCTCGTTCTTCACCACGCG | Rluc |
| Rluc-IRES-E73(DENV4)-NS3 | CGCGTGGTGAAGAACGAGCAGTAGGCGGCCG  CCCCTCTCCCTCCCCCCCCCCTAACGTTACTGG | GGTTCTAGTCTTGACATACTC (5700R) | IRES-E73(DENV4)-DENV2 |
| E-IRES-eGFP | CGTGACGCTGTATTTGGGAGTTATGGTGC  AGGCCTAGGCGGCCGCCCCTCTCCCTCCC | ACCCCGGTGAACAGCTCCTCGCCCTTGC  TCACCATGGTATTATCGTGTTTTTCAAAG | IRES-E73-DENV2 |
| IRES-eGFP-IRES | GGGGACGTGGTTTTCCTTTGAAAAACACGA  TAATACCATGGTGAGCAAGGGCGAGGAG | ACGTTAGGGGGGGGGGAGGGAGAGGGGC  GGCCGCCTACTTGTACAGCTCGTCCATGCC | eGFP |
| eGFP-IRES-E73(DENV4)-NS3 | GCCGGGATCACTCTCGGCATGGACGAGCT  GTACAAGTAGGCGGCCGCCCCTCTCCCTCC | GGTTCTAGTCTTGACATACTC (5700R) | IRES-E73(DENV4)-DENV2 |
| E-IRES-p53 | CGTGACGCTGTATTTGGGAGTTATGGTGC  AGGCCTAGGCGGCCGCCCCTCTCCCTCCC | GGGGCTCGACGCTAGGATCTGACTGCGGC  TCCTCCATGGTATTATCGTGTTTTTCAAAG | IRES-E73-DENV2 |
| IRES-p53-IRES | GGGACGTGGTTTTCCTTTGAAAAACACGA  TAATACCATGGAGGAGCCGCAGTCAGAT | TAACGTTAGGGGGGGGGGAGGGAGAGGGG  CGGCCGCTCAGTCTGAGTCAGGCCCTTCTG | P53 |
| P53-IRES-E73(DENV4)-NS3 | AACTCATGTTCAAGACAGAAGGGCCTGAC  TCAGACTGAGCGGCCGCCCCTCTCCCTCCC | GGTTCTAGTCTTGACATACTC (5700R) | IRES-E73(DENV4)-DENV2 |
| E-IRES-Mdm2 | CGTGACGCTGTATTTGGGAGTTATGGTGC  AGGCCTAGGCGGCCGCCCCTCTCCCTCCC | CATGTTGGTATTGCACATTTGCCTGCTCC  TCACCATGGTATTATCGTGTTTTTCAAAG | IRES-E73-DENV2 |
| IRES-Mdm2-IRES | GGGACGTGGTTTTCCTTTGAAAAACACGAT  AATACCATGGTGAGGAGCAGGCAAATGT | TAACGTTAGGGGGGGGGGAGGGAGAGGGG  CGGCCGCCTAGGGGAAATAAGTTAGCACA | Mdm2 |
| Mdm2-IRES-E73(DENV4)-NS3 | AACCAATTCAAATGATTGTGCTAACTTATT  TCCCCTAGGCGGCCGCCCCTCTCCCTCCC | GGTTCTAGTCTTGACATACTC (5700R) | IRES-E73(DENV4)-DENV2 |
| E-IRES-E25(DENV4) | CATGTCAGCGGCCATAAAAGA (2970F) | AACAGCTATGCACGTCATAGCCATTGAAG  TGTTCCTCATGGTATTATCGTGTTTTTCA | IRES-E73-DENV2 |
| IRES-E25(DENV4)-NS3 | GACGTGGTTTTCCTTTGAAAAACACGATAA  TACCATGAGGAACACTTCAATGGCTATG | GGTTCTAGTCTTGACATACTC (5700R) | IRES-E73(DENV4)-DENV2 |
| prM-GP160-IRES | C TGACAGCTGTCGCTCCTTCAATGACAATG  CGTTGCAGAGTGAGGGGGATACAGACGA | CGTTAGGGGGGGGGGGAGGGAGAGGGG  CGGCCGCTTATAGCAAGCCCCTTTCAAG | GP160 |
| C-IRES for replicon | CTGAAACGCGAGAGAAACCGCGTGTCG  ACTTAGCCGCCCCTCTCCCTCCCCCCCTA | TTCCTATAGATGTAAACACTCCTCCCAGG  GATCCCATGGTATTATCGTGTTTTTCAAA | EMCV-IRES |
| NS1-for-qPCR | CATGTCAGCGGCCATAAAAGA (2970F) | CACCACTGTGGTTCCTTCGCA (3260R) |  |

Supplement Table 2. Nucleotide and amino acid alterations in IRES-E73-DENV2

| Host cell | BHK-21 | BHK-21 | C6/36 |
| --- | --- | --- | --- |
| RNA | IRES-E73-DENV2 (1) | IRES-E73-DENV2 (2) | IRES-E73-DENV2 (2)* |
| virus Collection | 10 days p.e. | 18 days p.e.** | 10 days p.i. |
| 5’-UTR | ND | ND | ND |
| C | ND | ND | ND |
| prM | ND | ND | ND |
| E | ND | 1035 (T33 silent 6.4 %)  1338 (N135 silent 12.4 %)  1837 (M301L 5.6%) | ND |
| IRES | ND | 2939 (G to T 6.6%) | ND*** |
| E73 | ND | 3182 (L59P 14.8%) | ND*** |
| NS1 | ND | ND | ND |
| NS2A | ND | ND | ND |
| NS2B | ND | 5259 (S107 silent 16.0%) | ND |
| NS3 PRO | ND | ND | ND |
| NS3 HEL | 6015 (A229 silent 6.4%) | ND | ND |
| NS4A | ND | ND | ND |
| 2K | ND | ND | ND |
| NS4B | ND | ND | 8033 (Q134P 6.2%) |
| NS5 MT | ND | ND | ND |
| NS5 POL | 9334 (M320L 6.8%)  9345 (G323 silent 9.6%)  9362 (T329K 9.1%)  9385 (M337L 17.8%)  9397 (M341L 9.8%)  9401 (A342E 13.0%) | 9362 (T329K 9.4%)  9384 (P336 silent 6.6%)  9385 (M337L 21.7%)  9390 (V338 silent 5.9%)  9395 (Q340L 5.5%)  9396 (Q340H 6.8%)  9397 (M341L 12.8%)  9401 (A342E 19.2%)  9403 (M343L 5.8%) | 9345 (G323 silent 8.2%)  9362 (T329K 9.7%)  9385 (M337L 16.5%)  9397 (M341L 9.2%)  9401 (A342E 13.4%) |
| 3’-UTR | ND | ND | ND |

The cutoff for inclusion is > 5% abundance. The numbers refer to the nucleotide localization (1 to 11530) in IRES-E73-DENV2 RNA, followed by the corresponding original amino acid, location, mutated amino acid and frequency of occurrence are shown within parentheses. ND, not detected.

* Viruses were infected using a supernatant collected at 10 days p.e. from (2)

** Cell culture was conducted by supplying the fresh uninfected cells after 10 days p.e.

*** Matched fragment numbers are too low (<500).

Supplemental Table 3. Nucleotide and amino acid alterations in 3xIRES-E73-DENV2

| Host cell | BHK-21 | C6/36 |
| --- | --- | --- |
| RNA | 3x IRES-E73-DENV2 | 3x IRES-E73-DENV2* |
| virus Collection | 22 days p.e. | 10 days p.i. |
| 5’-UTR | ND | ND |
| C | ND | ND |
| prM | ND | ND |
| E | 1345 (Y137P 46.5%)  1658 (L241R 6.2%)  1786 (K284Q 5.9%)  1837 (M302L 5.2%)  2170 (M412L 5.1%) | **1345 (Y137P 92.1%)**  2170 (M412L 5.5%) |
| 3xIRES | 2924, 3731, 4538 (A to T 5.0%) ^a^  2928, 3735, 4542 (G to T 5.9%) ^a^  2939, 3746, 4553 (G to T 8.6%) ^a^ | 2939, 3746, 4553 (G to T 6.2%) ^a^ |
| 3xE73 | 3116, 3923, 4730 (I458L 5.2%) ^b^  4796 (V480A 5.2%) ^b^ | ND |
| NS1 | ND | ND |
| NS2A | ND | ND |
| NS2B | ND | 6873(S107 silent 14.5%) |
| NS3PRO | ND | ND |
| NS3HEL | 8041 (A367T 23.7%) | ND |
| NS4A | ND | ND |
| 2K | 9211 (I12L 8.0%) | ND |
| NS4B | 9568 (T108P 6.9%) | ND |
| NS5MT | ND | ND |
| NS5POL | 10948 (M320L 6.8%)  10959 (G323 silent 7.5%)  10973 (L328Q 5.4%)  10976 (T329K 12.0%)  10998 (P336 silent 6.2%)  10999 (M337L 21.4%)  11004 (V338 silent 5.7%)  11010 (Q340H 6.3%)  11011 (M341L 11.4%)  11015 (A342E 18.1%)  11017 (M343L5.4%) | 10948 (M320L 5.4%)  10959 (G323 silent 7.1%)  10976 (T329K 9.7%)  10998 (P336 silent 5.1%)  10999 (M337L 18.3%)  11010 (Q340H 5.2%)  11011 (M341L 9.6%)  11015 (A342E 15.9%) |
| 3’-UTR | ND | ND |

The cutoff for inclusion is > 5% abundance. The numbers refer to the nucleotide localization in the viral RNA, followed by the corresponding original amino acid, location, mutation and frequency of occurrence are shown within parentheses. Bald numbers and letters mean > 50% frequency of amino acid alteration. ND, not detected.

^a^ ^b^ Since the same sequences are repeated three times, it can’t be determined which one of those were mutated.

*Viruses were infected using a supernatant collected at 22 days p.e. in BHK-21 cells.

Supplemental Table 4. Nucleotide and amino acid alterations in IRES-Rluc-IRES-E73(DENV4)-DENV2 ^a^

| Host cell | BHK-21 | C6/36 |
| --- | --- | --- |
| RNA | IRES-Rluc-IRES-E73(DENV4)-DENV2 | IRES-Rluc-IRES-E73(DENV4)-DENV2 * |
| virus collection | 14 days p.e. | 10 days p.i. |
| 5’-UTR | ND | ND |
| C | ND | ND |
| prM | ND | ND |
| E | ND | ND |
| 2xIRES ^b^ | ND | 2924 (A to T 5.3%) ^b^  2939, 4457 (G to T 6.3%) ^b^ |
| Rluc | ND | ND |
| E73 | ND | ND |
| NS1 | ND | 5202 (S152 silent 7.6%) |
| NS2A | **6018 (V72 silent 99.8%)** | **6018 (V72 silent 99.5%)** |
| NS2B | ND | 6734 (Q93L 6.6%) |
| NS3  PRO | ND | ND |
| NS3  HEL | ND | ND |
| NS4A | ND | ND |
| 2K | ND | ND |
| NS4B | ND | 9215 (Q22P 8.4%) |
| NS5  MT | ND | ND |
| NS5  POL | 10903 (M337L 10.0%)  10915 (M341L 9.4%)  10919 (A342E 12.3%) | 10903 (M337L 24.1 %)  10913 (Q340L 5.6%)  10915 (M341L 12.4%)  10919 (A342E 19.7%)  10921 (M343L 5.5%)  10927 (D345H 6.8%) |
| 3’-UTR | ND | 12887 (G to C at 10562** 6.8%) |

The cutoff for inclusion is > 5% abundance. The numbers refer to the nucleotide localization in the viral RNA, followed by the corresponding original amino acid, location, mutation and frequency of occurrence are shown within parentheses. Bald numbers and letters mean > 50% frequency of amino acid alteration. ND, not detected.

^a^ In the IRES-X-IRES-E73(DENV4)-DENV2 construct, Rluc gene was integrate to “X” portion.

^b^ Since the same sequences are repeated twice (2x), it can’t be determined which one or both of the sequences got mutation(s).

* Viruses were infected, using the viruses from the supernatant collected from the electroporated BHK-21 cells.

** The nucleotide number corresponding to WT DENV2 was shown.

Supplemental Table 5. Nucleotide and amino acid alterations in p53- or Mdm2-integrated DENV2 ^a^

| Host cell | HEK-293 | HEK-293 |
| --- | --- | --- |
| RNA | IRES-p53-IRES--E73(DENV4)-DENV2 | IRES-Mdm2--E73(DENV4)-DENV2 |
| virus collection | 24 days p.e. | 30 days p.e. |
| 5’-UTR | ND | ND |
| C | ND | ND |
| prM | 804 (R122 silent 5.6%) | 759 (T107 silent 54.8%) |
| E | 1302 (K122 silent 34.8%)  1437 (R167 silent 9.2%)  2368 (S478T 10.8%) | 1134 (T66 silent 47.3%) |
| 2xIRES | ND | ND |
| p53 or Mdm2 | ND | ND* |
| E73 | ND | ND |
| NS1 | 5145 (E51 silent 9.1%) | ND |
| NS2A | ND | 6812 (S212L 7.7%) |
| NS2B | ND | ND |
| NS3PRO | ND | ND |
| NS3HEL | 7930 (L280 silent 11.7%) | ND |
| NS4A | ND | ND |
| 2K | ND | ND |
| NS4B | 9719 (T108I 5.3%)  9727 (L111F 12.3%)  9932 (T179I 18.2%)  10109 (S238F 8.3%) | 9856 (L111F 6.8%) |
| NS5MT | ND | ND |
| NS5POL | 11723 (G529E 5.8%, G529A 6.2%)  11725 (A530T 9.2%) | ND |
| 3’-UTR | 13133 (G to C at 10562** 12.3 %) | 13262 (G to C at 10562** 32.8%,  G to A 5.3%) |

The cutoff for inclusion is > 5% abundance. The numbers refer to the nucleotide localization in the viral RNA, followed by the corresponding original amino acid, location, mutation and frequency of occurrence are shown within parentheses. Bald numbers and letters mean > 50% frequency of amino acid alteration. ND, not detected.

^a^ In the IRES-X-IRES-E73(DENV4)-DENV2 construct, p53 or Mdm2 gene were integrate into “X” portion.

* Matched fragment numbers are too low (<500).

**The nucleotide number corresponding to DENV2 sequence.

Supplement Fig. 1


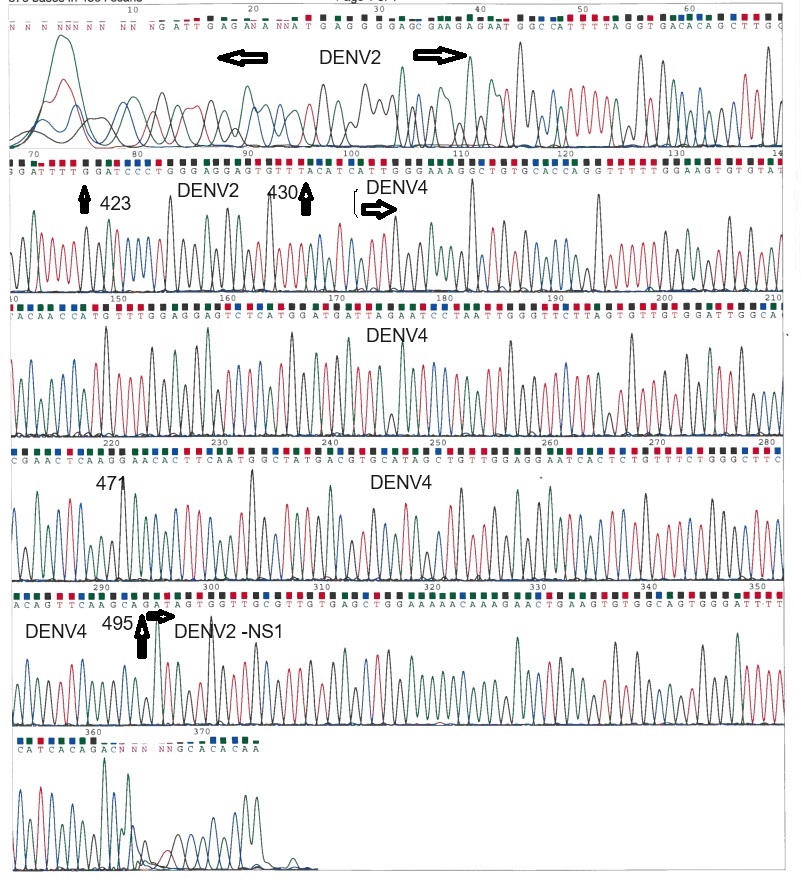


**Supplement Fig.1.** Sanger sequence of RT-PCR product, using primers amplifying DENV2, 2100~2500 nucleotide regions. The sequence data showed that the structure protein E recombined with the DENV4 sequences, which originally localized after the IRES sequence. Arrows show the portion of either DENV2 or DENV4 sequences.

Supplement Fig. 2


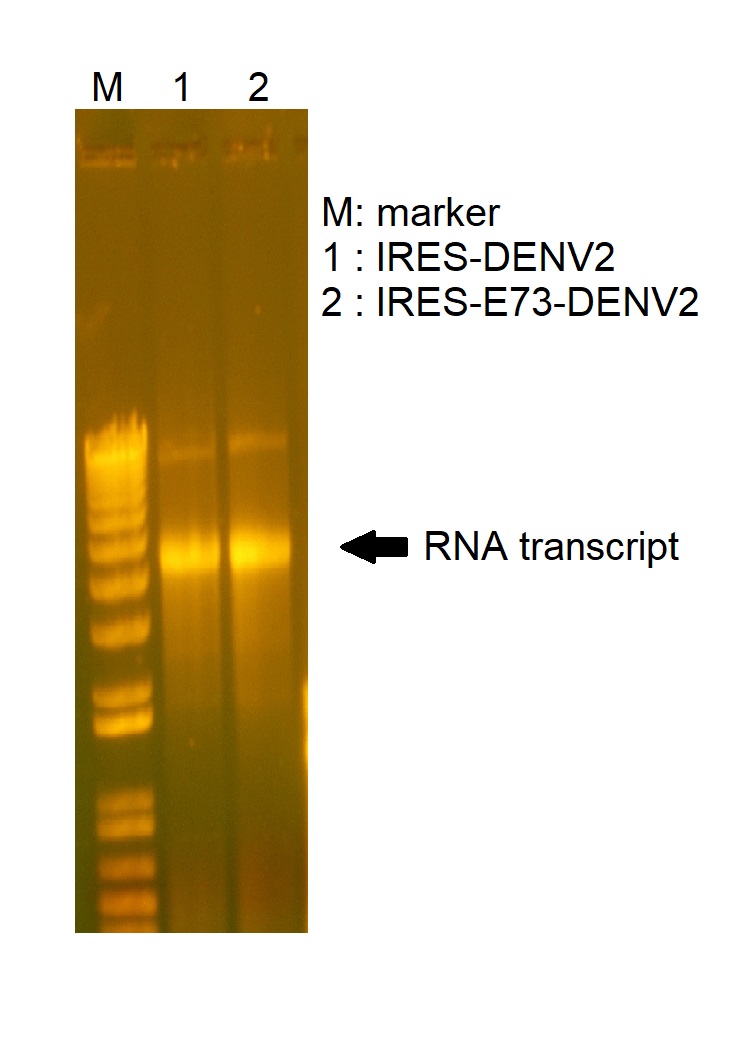


**Supplement Fig.2.** 0.8% agarose gel electrophoresis of the *in vitro*-transcribed RNAs. *in vitro*-transcribed RNA (from the specified cDNA construct) quantity and quality were examined.

Supplementary sequence data (cDNAs)

**IRES-E73-DENV2**

AGTTGTTAGTCTACGTGGACCGACAAAGACAGATTCTTTGAGGGAGCTAAGCTCAACGTAGTTCTAACAGTTTTTTAATTAGAGAGCAGATCTCTGATGAATAACCAACGAAAAAAGGCGAGAAATACGCCTTTCAATATGCTGAAACGCGAGAGAAACCGCGTGTCGACTGTACAACAGCTGACAAAGAGATTCTCACTTGGAATGCTGCAGGGACGAGGACCATTAAAACTGTTCATGGCCCTGGTGGCGTTCCTTCGTTTCCTAACAATCCCACCAACAGCAGGGATACTGAAGAGATGGGGAACAATTAAAAAATCAAAAGCCATTAATGTTTTGAGAGGGTTCAGGAAAGAGATTGGAAGGATGCTGAACATCTTGAACAGGAGACGCAGAACTGCAGGCATGATCATTATGCTGATTCCAACAGTGATGGCGTTCCATTTAACCACACGTAACGGAGAACCACACATGATCGTCAGTAGACAAGAGAAAGGGAAAAGTCTTCTGTTTAAAACAGAGGATGGTGTGAACATGTGTACCCTCATGGCCATGGACCTTGGTGAATTGTGTGAAGATACAATCACGTACAAGTGTCCTTTTCTCAAGCAGAATGAACCAGAAGACATAGATTGTTGGTGCAACTCTACGTCCACATGGGTAACTTATGGGACGTGTACCACCACAGGAGAACACAGAAGAGAAAAAAGATCAGTGGCACTCGTTCCACATGTGGGAATGGGACTGGAGACACGAACTGAAACATGGATGTCATCAGAAGGGGCCTGGAAACATGCCCAGAGAATTGAAACTTGGATCTTGAGACATCCAGGCTTTACCATAATGGCAGCAATCCTGGCATACACCATAGGAACGACACATTTCCAAAGAGCCCTGATTTTCATCTTACTGACAGCTGTCGCTCCTTCAATGACAATGCGTTGCATAGGAATATCAAATAGAGACTTTGTAGAAGGGGTTTCAGGAGGAAGCTGGGTTGACATAGTCTTAGAACATGGAAGCTGTGTGACGACGATGGCAAAAAACAAACCAACATTGGATTTTGAACTGATAAAAACAGAAGCCAAACAACCTGCCACTCTAAGGAAGTACTGTATAGAGGCAAAGCTGACCAACACAACAACAGATTCTCGCTGCCCAACACAAGGAGAACCCAGCCTAAATGAAGAGCAGGACAAAAGGTTCGTCTGCAAACACTCCATGGTGGACAGAGGATGGGGAAATGGATGTGGACTATTTGGAAAAGGAGGCATTGTGACCTGTGCTATGTTCACATGCAAAAAGAACATGAAAGGAAAAGTCGTGCAACCAGAAAACTTGGAATACACCATTGTGATAACACCTCACTCAGGGGAAGAGCATGCAGTCGGAAATGACACAGGAAAACATGGCAAGGAAATCAAAATAACACCACAGAGTTCCATCACAGAAGCAGAGTTGACAGGCTATGGCACTGTCACGATGGAGTGCTCTCCGAGAACGGGCCTCGACTTCAATGAGATGGTGTTGCTGCAAATGGAAAATAAAGCTTGGCTGGTGCACAGGCAATGGTTCCTAGACCTGCCGTTGCCATGGCTGCCCGGAGCGGACACACAAGGATCAAATTGGATACAGAAAGAGACATTGGTCACTTTCAAAAATCCCCATGCGAAGAAACAGGATGTTGTTGTTTTGGGATCCCAAGAAGGGGCCATGCACACAGCACTCACAGGGGCCACAGAAATCCAGATGTCATCAGGAAACTTACTGTTCACAGGACATCTCAAGTGCAGGCTGAGGATGGACAAACTACAGCTCAAAGGAATGTCATACTCTATGTGCACAGGAAAGTTTAAAGTTGTGAAGGAAATAGCAGAAACACAACATGGAACAATAGTTATCAGAGTACAATATGAAGGGGACGGTTCTCCATGTAAGATCCCTTTTGAGATAATGGATTTGGAAAAAAGACATGTTTTAGGTCGCCTGATTACAGTCAACCCAATCGTAACAGAAAAAGATAGCCCAGTCAACATAGAAGCAGAACCTCCATTCGGAGACAGCTACATCATCATAGGAGTAGAGCCGGGACAATTGAAGCTCAACTGGTTTAAGAAAGGAAGTTCTATCGGCCAAATGATTGAGACAACAATGAGGGGAGCGAAGAGAATGGCCATTTTAGGTGACACAGCTTGGGATTTTGGATCCCTGGGAGGAGTGTTTACATCTATAGGAAAGGCTCTCCACCAAGTTTTCGGAGCAATCTATGGGGCTGCCTTCAGTGGGGTCTCATGGATTATGAAAATCCTCATAGGAGTCATTATCACATGGATAGGAATGAATTCACGCAGCACCTCACTGTCTGTGTCACTAGTATTGGTGGGAGTCGTGACGCTGTATTTGGGAGTTATGGTGCAGGCCTAGGCGGCCGCCCCTCTCCCTCCCCCCCCCCTAACGTTACTGGCCGAAGCCGCTTGGAATAAGGCCGGTGTGCGTTTGTCTATATGTTATTTTCCACCATATTGCCGTCTTTTGGCAATGTGAGGGCCCGGAAACCTGGCCCTGTCTTCTTGACGAGCATTCCTAGGGGTCTTTCCCCTCTCGCCAAAGGAATGCAAGGTCTGTTGAATGTCGTGAAGGAAGCAGTTCCTCTGGAAGCTTCTTGAAGACAAACAACGTCTGTAGCGACCCTTTGCAGGCAGCGGAACCCCCCACCTGGCGACAGGTGCCTCTGCGGCCAAAAGCCACGTGTATAAGATACACCTGCAAAGGCGGCACAACCCCAGTGCCACGTTGTGAGTTGGATAGTTGTGGAAAGAGTCAAATGGCTCTCCTCAAGCGTATTCAACAAGGGGCTGAAGGATGCCCAGAAGGTACCCCATTGTATGGGATCTGATCTGGGGCCTCGGTGCACATGCTTTACATGTGTTTAGTCGAGGTTAAAAAAACGTCTAGGCCCCCCGAACCACGGGGACGTGGTTTTCCTTTGAAAAACACGATAATACCATGGGATCCCTGGGAGGAGTGTTTACATCTATAGGAAAGGCTCTCCACCAAGTTTTCGGAGCAATCTATGGGGCTGCCTTCAGTGGGGTCTCATGGATTATGAAAATCCTCATAGGAGTCATTATCACATGGATAGGAATGAATTCACGCAGCACCTCACTGTCTGTGTCACTAGTATTGGTGGGAGTCGTGACGCTGTATTTGGGAGTTATGGTGCAGGCCGATAGTGGTTGCGTTGTGAGCTGGAAAAACAAAGAACTGAAGTGTGGCAGTGGGATTTTCATCACAGACAACGTGCACACATGGACAGAACAATACAAGTTCCAACCAGAATCCCCTTCAAAACTAGCTTCAGCTATCCAGAAAGCTCATGAAGAGGGCATTTGTGGAATCCGCTCAGTAACAAGACTGGAAAATCTGATGTGGAAACAAATAACACCAGAATTGAATCACATTCTATCAGAAAATGAGGTGAAGTTGACTATTATGACAGGAGACATCAAAGGAATCATGCAGGCAGGAAAACGATCTCTGCAGCCCCAGCCCACTGAGCTGAAGTATTCATGGAAAACATGGGGCAAAGCGAAAATGCTCTCTACAGAGTCTCATAACCAGACCTTTCTCATTGATGGCCCCGAAACAGCAGAATGCCCCAACACAAACAGAGCTTGGAATTCGCTGGAAGTTGAAGACTATGGCTTTGGAGTATTCACCACCAATATATGGCTAAAGTTGAGAGAAAAGCAGGATGTATTCTGCGACTCAAAACTCATGTCAGCGGCCATAAAAGACAACAGAGCCGTCCATGCCGATATGGGTTATTGGATAGAAAGTGCACTCAATGACACATGGAAGATAGAGAAAGCCTCTTTCATCGAAGTTAAAAGCTGCCACTGGCCAAAGTCACACACCCTCTGGAGTAATGGAGTGTTAGAAAGTGAGATGATAATTCCAAAGAATTTCGCTGGACCAGTGTCACAACACAACTACAGACCAGGTTACCATACACAAACAGCAGGACCATGGCATCTAGGTAAGCTTGAGATGGACTTTGATTTCTGCGAAGGAACCACAGTGGTGGTGACTGAGGACTGTGGAAATAGAGGACCCTCTTTAAGAACAACTACTGCCTCTGGAAAACTCATAACAGAATGGTGCTGCCGATCTTGCACATTACCACCGCTAAGATACAGAGGTGAGGACGGATGCTGGTACGGGATGGAAATCAGACCATTGAAAGAGAAAGAAGAGAATTTGGTCAACTCCTTGGTCACAGCCGGACATGGGCAGATTGACAACTTTTCACTAGGAGTCTTGGGAATGGCATTGTTCCTGGAAGAAATGCTCAGGACCCGAGTAGGAACGAAACATGCAATACTACTAGTTGCAGTTTCTTTTGTGACATTGATCACAGGGAACATGTCCTTTAGAGACCTGGGAAGAGTGATGGTTATGGTGGGCGCTACTATGACGGATGACATAGGTATGGGCGTGACTTATCTTGCCCTACTAGCAGCCTTCAAAGTCAGACCAACTTTTGCAGCCGGACTACTCTTGAGAAAGTTGACCTCCAAGGAATTGATGATGACTACCATAGGAATCGTACTCCTCTCCCAGAGCACCATACCAGAGACCATTCTTGAACTGACTGATGCGTTAGCCTTGGGCATGATGGTCCTTAAAATGGTGAGAAAAATGGAAAAGTATCAATTGGCAGTGACTATCATGGCTATCTTGTGCGTCCCAAATGCAGTGATATTACAAAACGCATGGAAAGTGAGTTGCACAATATTGGCAGTGGTGTCCGTTTCCCCACTGTTCTTAACATCCTCACAGCAGAAAGCGGATTGGATACCATTAGCATTGACGATCAAGGGTCTCAATCCAACAGCTATTTTTCTAACAACCCTTTCAAGAACCAACAAGAAAAGGAGCTGGCCACTAAATGAGGCTATCATGGCAGTCGGGATGGTGAGCATTTTGGCCAGTTCACTCCTAAAGAATGACATTCCCATGACAGGACCATTAGTGGCTGGAGGGCTCCTCACTGTGTGCTACGTGCTCACTGGACGATCGGCCGATTTGGAACTGGAGAGAGCCGCCGATGTCAAATGGGAAGATCAGGCAGAGATATCAGGAAGCAGTCCAATCCTGTCAATAACAATATCAGAAGATGGTAGCATGTCGATAAAAAACGAAGAGGAAGAACAAACACTGACCATACTCATTAGAACAGGATTGCTGGTGATCTCAGGACTTTTTCCTGTATCAATACCAATCACGGCAGCAGCATGGTACCTGTGGGAAGTGAAGAAACAACGGGCTGGAGTATTGTGGGATGTCCCTTCACCCCCACCCGTGGGAAAGGCTGAACTGGAAGATGGAGCCTATAGAATCAAGCAAAAAGGGATTCTTGGATATTCCCAGATCGGAGCCGGAGTTTACAAAGAAGGAACATTCCATACAATGTGGCATGTCACACGCGGCGCTGTTCTAATGCATAAAGGAAAGAGGATTGAACCATCATGGGCGGACGTTAAGAAAGACCTAATATCATATGGAGGAGGCTGGAAGCTAGAAGGAGAATGGAAGGAAGGAGAAGAAGTCCAGGTCTTGGCATTGGAGCCTGGAAAAAATCCAAGAGCCGTCCAAACAAAACCTGGTCTTTTCAAAACCAACGCCGGAACCATAGGTGCCGTATCTCTGGACTTTTCTCCTGGAACCTCAGGATCTCCAATCATCGACAAAAAAGGAAAAGTTGTGGGTCTTTATGGTAATGGTGTTGTTACAAGGAGTGGAGCATATGTGAGTGCTATAGCCCAGACTGAAAAAAGTATTGAAGACAATCCAGAGATCGAAGATGACATTTTTCGAAAGAGAAAATTGACCATCATGGACCTCCACCCAGGAGCGGGAAAGACGAAGAGATACCTTCCGGCCATAGTCAGAGAGGCTATAAAACGGGGCCTGAGGACATTAATCCTGGCCCCCACTAGAGTCGTGGCAGCTGAAATGGAGGAAGCCCTAAGAGGACTTCCAATAAGATACCAAACCCCAGCCATCAGAGCTGAGCACACCGGGCGGGAGATTGTGGACCTAATGTGTCATGCCACATTCACTATGAGGCTGCTATCACCAGTTAGAGTGCCAAATTACAACCTGATCATCATGGACGAAGCCCATTTCACAGACCCAGCAAGTATAGCGGCTAGAGGATACATCTCAACTCGAGTAGAGATGGGTGAGGCAGCTGGGATTTTCATGACAGCCACTCCTCCGGGAAGCAGAGACCCATTCCCTCAGAGCAATGCACCAATCATGGATGAAGAAAGAGAAATCCCTGAACGTTCGTGGAGTTCTGGACATGAGTGGGTCACGGATTTTAAAGGGAAGACTGTTTGGTTCGTTCCAAGTATAAAAGCAGGAAATGATATAGCAGCTTGCCTGAGAAAAAATGGAAAGAAAGTGATACAACTCAGTAGGAAGACCTTTGATTCTGAGTATGTCAAGACTAGAACCAATGATTGGGACTTCGTGGTCACAACTGACATTTCAGAAATGGGTGCCAACTTCAAGGCTGAGAGGGTTATAGACCCCAGACGCTGCATGAAACCAGTTATACTAACAGATGGTGAAGAGCGGGTGATCCTGGCAGGACCTATGCCAGTGACCCACTCTAGTGCAGCACAAAGAAGAGGGAGAATAGGAAGAAATCCAAAAAATGAAAATGACCAGTACATATACATGGGGGAACCTCTGGAAAATGATGAAGACTGTGCACACTGGAAAGAAGCTAAAATGCTCCTAGATAACATCAACACACCTGAAGGAATCATTCCTAGCATGTTCGAACCAGAGCGTGAAAAGGTGGATGCCATTGATGGTGAATACCGCTTGAGAGGAGAAGCAAGGAAAACCTTTGTGGACCTAATGAGAAGAGGAGACCTACCAGTCTGGTTGGCCTACAGAGTGGCAGCTGAAGGCATCAACTACGCAGACAGAAGGTGGTGTTTTGATGGAATTAAGAACAACCAAATCTTGGAAGAAAATGTGGAGGTGGAAATCTGGACAAAAGAAGGGGAAAGGAAGAAATTAAAACCCAGATGGTTGGATGCCAGGATCTACTCTGACCCACTGGCGCTAAAGGAATTCAAGGAGTTTGCAGCTGGAAGAAAGTCCCTGACCCTGAACCTAATCACAGAAATGGGTAGGCTTCCAACTTTCATGACTCAGAAGGCAAGAGACGCACTGGACAACTTAGCAGTGCTGCACACGGCTGAAGCAGGTGGAAGGGCGTACAATCATGCTCTCAGTGAACTGCCGGAGACCCTGGAGACATTGCTTTTACTGACACTTCTGGCTACAGTCACAGGAGGAATCTTTTTATTCTTGATGAGCGGAAGGGGTATAGGGAAGATGACCCTGGGAATGTGCTGCATAATCACGGCTAGTATTCTCCTATGGTACGCACAAATACAGCCACACTGGATAGCAGCTTCAATAATACTGGAGTTTTTTCTCATAGTTTTGCTTATTCCAGAACCAGAAAAGCAGAGAACACCCCAAGATAACCAATTGACCTACGTTGTCATAGCCATCCTCACAGTGGTGGCCGCAACCATGGCAAACGAGATGGGTTTCCTGGAAAAAACGAAGAAAGATCTCGGATTGGGAAGCATTACAACCCAGCAACCCGAGAGCAACATCCTGGACATAGATCTACGTCCCGCATCAGCATGGACGCTGTATGCTGTGGCCACAACATTTGTCACACCAATGTTGAGACACAGCATTGAAAATTCCTCAGTGAACGTGTCCCTAACAGCTATTGCCAACCAAGCCACAGTGTTAATGGGTCTTGGGAAAGGATGGCCATTGTCAAAGATGGACATCGGAGTTCCCCTTCTCGCCATTGGATGCTACTCACAAGTCAACCCCATAACTCTCACAGCAGCTCTTTTCTTACTGGTAGCACATTATGCCATCATAGGGCCAGGACTCCAAGCAAAAGCAACCAGAGAAGCTCAGAAAAGAGCAGCAGCGGGCATCATGAAAAACCCAACTGTCGATGGAATAACAGTGATTGACCTAGATCCAATACCCTATGATCCAAAGTTTGAAAAGCAGTTGGGACAAGTAATGCTCCTAGTCCTCTGCGTGACTCAAGTGTTGATGATGAGGACTACATGGGCTCTGTGTGAGGCTTTAACCTTAGCGACCGGGCCTATCTCCACATTGTGGGAAGGAAATCCAGGGAGGTTTTGGAACACTACCATTGCAGTGTCAATGGCTAACATTTTTAGAGGGAGTTACTTGGCCGGAGCTGGACTTCTCTTTTCCATCATGAAGAACACAACCAACACGAGAAGGGGAACTGGCAACATAGGAGAGACGCTTGGAGAGAAATGGAAAAGCCGATTGAACGCATTGGGGAAAAGTGAATTCCAGATCTACAAGAAAAGTGGAATCCAGGAAGTGGATAGAACCTTAGCAAAAGAAGGCATTAAAAGAGGAGAAACGGACCATCACGCTGTGTCGCGAGGCTCAGCAAAACTGAGATGGTTCGTCGAGAGAAATATGGTCACACCAGAAGGGAAAGTAGTGGACCTCGGTTGCGGCAGAGGAGGCTGGTCATACTATTGTGGGGGACTAAAGAATGTAAGAGAAGTCAAAGGCCTAACAAAAGGAGGACCAGGACATGAAGAACCCATCCCCATGTCAACATATGGGTGGAATCTAGTACGTCTTCAAAGTGGAGTTGACGTTTTCTTCACTCCGCCAGAAAAGTGTGACACATTGTTGTGTGACATAGGGGAGTCGTCACCAAATCCCACGGTAGAAGCAGGACGAACACTCAGAGTCCTTAACTTAGTGGAAAATTGGTTGAACAACAACACCCAATTTTGCATAAAGGTTCTCAACCCATACATGCCCTCAGTCATAGAAAAAATGGAAGCACTACAAAGGAAATATGGAGGAGCCTTAGTGAGGAATCCACTCTCACGAAACTCCACACATGAGATGTACTGGGTATCCAATGCCTCCGGGAACATAGTGTCATCAGTGAACATGATTTCAAGGATGTTGATCAACAGATTCACAATGAGACACAAGAAAGCCACTTACGAGCCAGATGTAGACCTCGGAAGCGGAACCCGCAACATCGGAATTGAAAGTGAGATACCAAACCTAGACATAATCGGGAAAAGAATAGAAAAAATAAAACAAGAGCATGAAACATCATGGCACTATGACCAAGACCACCCATACAAAACGTGGGCTTACCATGGCAGCTATGAAACAAAACAAACTGGATCAGCATCATCCATGGTGAACGGAGTGGTCAGACTGCTGACAAAACCTTGGGACGTCGTCCCCATGGTGACACAGATGGCAATGACAGACACGACTCCATTTGGACAACAGCGCGTTTTTAAAGAAAAAGTGGACACGAGAACCCAAGAACCGAAAGAAGGCACAAAGAAACTAATGAAAATCACGGCAGAGTGGCTTTGGAAAGAACTAGGGAAGAAAAAGACACCTAGGATGTGCACTAGAGAAGAATTCACAAGAAAGGTGAGAAGCAATGCAGCCTTGGGGGCCATATTCACTGATGAGAACAAGTGGAAGTCGGCACGTGAGGCTGTTGAAGATAGTAGGTTTTGGGAGCTGGTTGACAAGGAAAGGAATCTCCATCTTGAAGGAAAGTGTGAAACATGTGTGTATAACATGATGGGAAAAAGAGAGAAGAAGCTAGGGGAGTTCGGCAAGGCAAAAGGCAGCAGAGCCATATGGTACATGTGGCTTGGAGCACGCTTCTTAGAGTTTGAAGCCCTAGGATTCTTGAATGAAGATCACTGGTTCTCCAGAGAGAACTCCTTGAGTGGAGTGGAAGGAGAAGGGCTGCACAAGCTAGGTTACATTTTAAGAGACGTGAGCAAGAAAGAGGGAGGAGCAATGTATGCCGATGACACCGCAGGATGGGACACAAGAATCACACTAGAAGACCTAAAAAATGAAGAAATGGTAACAAACCACATGGAAGGAGAACACAAGAAACTAGCCGAGGCCATTTTCAAATTAACGTACCAAAACAAGGTGGTGCGTGTGCAAAGACCAACACCAAGAGGCACAGTAATGGATATCATATCGAGAAGAGACCAAAGAGGTAGTGGACAAGTTGGTACCTATGGACTCAATACTTTCACCAATATGGAAGCCCAACTAATCAGACAGATGGAGGGAGAAGGAGTCTTCAAAAGCATTCAGCACCTGACAGTCACAGAAGAAATCGCCGTGCAAAACTGGTTAGCAAGAGTAGGGCGCGAAAGGTTATCAAGAATGGCCATCAGTGGAGATGATTGTGTTGTGAAACCTTTAGATGACAGGTTCGCAAGCGCTTTAACAGCTCTAAATGACATGGGAAAGGTTAGGAAAGACATACAACAATGGGAACCTTCAAGAGGATGGAACGATTGGACACAAGTGCCCTTCTGTTCACACCATTTCCATGAGTTAATCATGAAAGACGGCCGCGTACTTGTAGTTCCATGCAGAAACCAAGATGAACTGATTGGTAGAGCCCGAATTTCCCAAGGAGCTGGGTGGTCTTTGCGAGAGACGGCCTGTTTGGGGAAGTCCTACGCCCAAATGTGGAGCTTGATGTACTTCCACAGACGTGACCTCAGGCTGGCGGCTAATGCTATTTGCTCGGCAGTCCCATCACATTGGGTTCCAACAAGTAGAACAACCTGGTCCATACACGCCAAACATGAATGGATGACAACGGAAGACATGCTGACAGTCTGGAACAGGGTGTGGATTCAAGAAAACCCATGGATGGAAGACAAAACTCCAGTGGAATCATGGGAGGAAATCCCATACTTGGGGAAAAGAGAAGACCAATGGTGCGGCTCATTGATTGGGCTAACAAGCAGGGCCACCTGGGCAAAGAACATCCAAACAGCAATAAATCAAGTTAGATCCCTTATAGGCTATGAGGAATACACAGATTACATGCCATCCATGAAAAGATTCAGAAGAGAAGAGGAAGAGGCAGGAGTCCTGTGGTAGAAGGCAAAACTAACATGAAACAAGGCTAGAAGTCAGGTCGGATTAAGCCATAGTACGGAAAAAACTATGCTACCTGTGAGCCCCGTCCAAGGACGTTAAAAGAAGTCAGGCCATTACAAATGCCATAGCTTGAGTAAACTGAGCAGCCTGTAGCTCCACCTGAGAAGGTGTAAAAAATCTGGGAGGCCACAAACCATGGAAGCTGTACGCATGGCGTAGTGGACTAGCGGTTAGAGGAGACCCCTCCCTTACAAATCGCAGCAACAATGGGGGCCCAAGGTGAGATGAAGCTGTAGTCTCACTGGAAGGACTAGAGGTTAGAGGAGACCCCCCCAAAACAAAAAACAGCATATTGACGCTGGGAAAGACCAGAGATCCTGCTGTCTCCTCAGCATCATTCCAGGCACAGAACGCCAGAAAATGGAATGGTGCTGTTGAATCAACAGGTTCT

**3XIRES-E73-DENV2**

AGTTGTTAGTCTACGTGGACCGACAAAGACAGATTCTTTGAGGGAGCTAAGCTCAACGTAGTTCTAACAGTTTTTTAATTAGAGAGCAGATCTCTGATGAATAACCAACGAAAAAAGGCGAGAAATACGCCTTTCAATATGCTGAAACGCGAGAGAAACCGCGTGTCGACTGTACAACAGCTGACAAAGAGATTCTCACTTGGAATGCTGCAGGGACGAGGACCATTAAAACTGTTCATGGCCCTGGTGGCGTTCCTTCGTTTCCTAACAATCCCACCAACAGCAGGGATACTGAAGAGATGGGGAACAATTAAAAAATCAAAAGCCATTAATGTTTTGAGAGGGTTCAGGAAAGAGATTGGAAGGATGCTGAACATCTTGAACAGGAGACGCAGAACTGCAGGCATGATCATTATGCTGATTCCAACAGTGATGGCGTTCCATTTAACCACACGTAACGGAGAACCACACATGATCGTCAGTAGACAAGAGAAAGGGAAAAGTCTTCTGTTTAAAACAGAGGATGGTGTGAACATGTGTACCCTCATGGCCATGGACCTTGGTGAATTGTGTGAAGATACAATCACGTACAAGTGTCCTTTTCTCAAGCAGAATGAACCAGAAGACATAGATTGTTGGTGCAACTCTACGTCCACATGGGTAACTTATGGGACGTGTACCACCACAGGAGAACACAGAAGAGAAAAAAGATCAGTGGCACTCGTTCCACATGTGGGAATGGGACTGGAGACACGAACTGAAACATGGATGTCATCAGAAGGGGCCTGGAAACATGCCCAGAGAATTGAAACTTGGATCTTGAGACATCCAGGCTTTACCATAATGGCAGCAATCCTGGCATACACCATAGGAACGACACATTTCCAAAGAGCCCTGATTTTCATCTTACTGACAGCTGTCGCTCCTTCAATGACAATGCGTTGCATAGGAATATCAAATAGAGACTTTGTAGAAGGGGTTTCAGGAGGAAGCTGGGTTGACATAGTCTTAGAACATGGAAGCTGTGTGACGACGATGGCAAAAAACAAACCAACATTGGATTTTGAACTGATAAAAACAGAAGCCAAACAACCTGCCACTCTAAGGAAGTACTGTATAGAGGCAAAGCTGACCAACACAACAACAGATTCTCGCTGCCCAACACAAGGAGAACCCAGCCTAAATGAAGAGCAGGACAAAAGGTTCGTCTGCAAACACTCCATGGTGGACAGAGGATGGGGAAATGGATGTGGACTATTTGGAAAAGGAGGCATTGTGACCTGTGCTATGTTCACATGCAAAAAGAACATGAAAGGAAAAGTCGTGCAACCAGAAAACTTGGAATACACCATTGTGATAACACCTCACTCAGGGGAAGAGCATGCAGTCGGAAATGACACAGGAAAACATGGCAAGGAAATCAAAATAACACCACAGAGTTCCATCACAGAAGCAGAGTTGACAGGCTATGGCACTGTCACGATGGAGTGCTCTCCGAGAACGGGCCTCGACTTCAATGAGATGGTGTTGCTGCAAATGGAAAATAAAGCTTGGCTGGTGCACAGGCAATGGTTCCTAGACCTGCCGTTGCCATGGCTGCCCGGAGCGGACACACAAGGATCAAATTGGATACAGAAAGAGACATTGGTCACTTTCAAAAATCCCCATGCGAAGAAACAGGATGTTGTTGTTTTGGGATCCCAAGAAGGGGCCATGCACACAGCACTCACAGGGGCCACAGAAATCCAGATGTCATCAGGAAACTTACTGTTCACAGGACATCTCAAGTGCAGGCTGAGGATGGACAAACTACAGCTCAAAGGAATGTCATACTCTATGTGCACAGGAAAGTTTAAAGTTGTGAAGGAAATAGCAGAAACACAACATGGAACAATAGTTATCAGAGTACAATATGAAGGGGACGGTTCTCCATGTAAGATCCCTTTTGAGATAATGGATTTGGAAAAAAGACATGTTTTAGGTCGCCTGATTACAGTCAACCCAATCGTAACAGAAAAAGATAGCCCAGTCAACATAGAAGCAGAACCTCCATTCGGAGACAGCTACATCATCATAGGAGTAGAGCCGGGACAATTGAAGCTCAACTGGTTTAAGAAAGGAAGTTCTATCGGCCAAATGATTGAGACAACAATGAGGGGAGCGAAGAGAATGGCCATTTTAGGTGACACAGCTTGGGATTTTGGATCCCTGGGAGGAGTGTTTACATCTATAGGAAAGGCTCTCCACCAAGTTTTCGGAGCAATCTATGGGGCTGCCTTCAGTGGGGTCTCATGGATTATGAAAATCCTCATAGGAGTCATTATCACATGGATAGGAATGAATTCACGCAGCACCTCACTGTCTGTGTCACTAGTATTGGTGGGAGTCGTGACGCTGTATTTGGGAGTTATGGTGCAGGCCTAGGCGGCCGCCCCTCTCCCTCCCCCCCCCCTAACGTTACTGGCCGAAGCCGCTTGGAATAAGGCCGGTGTGCGTTTGTCTATATGTTATTTTCCACCATATTGCCGTCTTTTGGCAATGTGAGGGCCCGGAAACCTGGCCCTGTCTTCTTGACGAGCATTCCTAGGGGTCTTTCCCCTCTCGCCAAAGGAATGCAAGGTCTGTTGAATGTCGTGAAGGAAGCAGTTCCTCTGGAAGCTTCTTGAAGACAAACAACGTCTGTAGCGACCCTTTGCAGGCAGCGGAACCCCCCACCTGGCGACAGGTGCCTCTGCGGCCAAAAGCCACGTGTATAAGATACACCTGCAAAGGCGGCACAACCCCAGTGCCACGTTGTGAGTTGGATAGTTGTGGAAAGAGTCAAATGGCTCTCCTCAAGCGTATTCAACAAGGGGCTGAAGGATGCCCAGAAGGTACCCCATTGTATGGGATCTGATCTGGGGCCTCGGTGCACATGCTTTACATGTGTTTAGTCGAGGTTAAAAAAACGTCTAGGCCCCCCGAACCACGGGGACGTGGTTTTCCTTTGAAAAACACGATAATACCATGGGATCCCTGGGAGGAGTGTTTACATCTATAGGAAAGGCTCTCCACCAAGTTTTCGGAGCAATCTATGGGGCTGCCTTCAGTGGGGTCTCATGGATTATGAAAATCCTCATAGGAGTCATTATCACATGGATAGGAATGAATTCACGCAGCACCTCACTGTCTGTGTCACTAGTATTGGTGGGAGTCGTGACGCTGTATTTGGGAGTTATGGTGCAGGCCTAGGCGGCCGCCCCTCTCCCTCCCCCCCCCCTAACGTTACTGGCCGAAGCCGCTTGGAATAAGGCCGGTGTGCGTTTGTCTATATGTTATTTTCCACCATATTGCCGTCTTTTGGCAATGTGAGGGCCCGGAAACCTGGCCCTGTCTTCTTGACGAGCATTCCTAGGGGTCTTTCCCCTCTCGCCAAAGGAATGCAAGGTCTGTTGAATGTCGTGAAGGAAGCAGTTCCTCTGGAAGCTTCTTGAAGACAAACAACGTCTGTAGCGACCCTTTGCAGGCAGCGGAACCCCCCACCTGGCGACAGGTGCCTCTGCGGCCAAAAGCCACGTGTATAAGATACACCTGCAAAGGCGGCACAACCCCAGTGCCACGTTGTGAGTTGGATAGTTGTGGAAAGAGTCAAATGGCTCTCCTCAAGCGTATTCAACAAGGGGCTGAAGGATGCCCAGAAGGTACCCCATTGTATGGGATCTGATCTGGGGCCTCGGTGCACATGCTTTACATGTGTTTAGTCGAGGTTAAAAAAACGTCTAGGCCCCCCGAACCACGGGGACGTGGTTTTCCTTTGAAAAACACGATAATACCATGGGATCCCTGGGAGGAGTGTTTACATCTATAGGAAAGGCTCTCCACCAAGTTTTCGGAGCAATCTATGGGGCTGCCTTCAGTGGGGTCTCATGGATTATGAAAATCCTCATAGGAGTCATTATCACATGGATAGGAATGAATTCACGCAGCACCTCACTGTCTGTGTCACTAGTATTGGTGGGAGTCGTGACGCTGTATTTGGGAGTTATGGTGCAGGCCTAGGCGGCCGCCCCTCTCCCTCCCCCCCCCCTAACGTTACTGGCCGAAGCCGCTTGGAATAAGGCCGGTGTGCGTTTGTCTATATGTTATTTTCCACCATATTGCCGTCTTTTGGCAATGTGAGGGCCCGGAAACCTGGCCCTGTCTTCTTGACGAGCATTCCTAGGGGTCTTTCCCCTCTCGCCAAAGGAATGCAAGGTCTGTTGAATGTCGTGAAGGAAGCAGTTCCTCTGGAAGCTTCTTGAAGACAAACAACGTCTGTAGCGACCCTTTGCAGGCAGCGGAACCCCCCACCTGGCGACAGGTGCCTCTGCGGCCAAAAGCCACGTGTATAAGATACACCTGCAAAGGCGGCACAACCCCAGTGCCACGTTGTGAGTTGGATAGTTGTGGAAAGAGTCAAATGGCTCTCCTCAAGCGTATTCAACAAGGGGCTGAAGGATGCCCAGAAGGTACCCCATTGTATGGGATCTGATCTGGGGCCTCGGTGCACATGCTTTACATGTGTTTAGTCGAGGTTAAAAAAACGTCTAGGCCCCCCGAACCACGGGGACGTGGTTTTCCTTTGAAAAACACGATAATACCATGGGATCCCTGGGAGGAGTGTTTACATCTATAGGAAAGGCTCTCCACCAAGTTTTCGGAGCAATCTATGGGGCTGCCTTCAGTGGGGTCTCATGGATTATGAAAATCCTCATAGGAGTCATTATCACATGGATAGGAATGAATTCACGCAGCACCTCACTGTCTGTGTCACTAGTATTGGTGGGAGTCGTGACGCTGTATTTGGGAGTTATGGTGCAGGCCGATAGTGGTTGCGTTGTGAGCTGGAAAAACAAAGAACTGAAGTGTGGCAGTGGGATTTTCATCACAGACAACGTGCACACATGGACAGAACAATACAAGTTCCAACCAGAATCCCCTTCAAAACTAGCTTCAGCTATCCAGAAAGCTCATGAAGAGGGCATTTGTGGAATCCGCTCAGTAACAAGACTGGAAAATCTGATGTGGAAACAAATAACACCAGAATTGAATCACATTCTATCAGAAAATGAGGTGAAGTTGACTATTATGACAGGAGACATCAAAGGAATCATGCAGGCAGGAAAACGATCTCTGCAGCCCCAGCCCACTGAGCTGAAGTATTCATGGAAAACATGGGGCAAAGCGAAAATGCTCTCTACAGAGTCTCATAACCAGACCTTTCTCATTGATGGCCCCGAAACAGCAGAATGCCCCAACACAAACAGAGCTTGGAATTCGCTGGAAGTTGAAGACTATGGCTTTGGAGTATTCACCACCAATATATGGCTAAAGTTGAGAGAAAAGCAGGATGTATTCTGCGACTCAAAACTCATGTCAGCGGCCATAAAAGACAACAGAGCCGTCCATGCCGATATGGGTTATTGGATAGAAAGTGCACTCAATGACACATGGAAGATAGAGAAAGCCTCTTTCATCGAAGTTAAAAGCTGCCACTGGCCAAAGTCACACACCCTCTGGAGTAATGGAGTGTTAGAAAGTGAGATGATAATTCCAAAGAATTTCGCTGGACCAGTGTCACAACACAACTACAGACCAGGTTACCATACACAAACAGCAGGACCATGGCATCTAGGTAAGCTTGAGATGGACTTTGATTTCTGCGAAGGAACCACAGTGGTGGTGACTGAGGACTGTGGAAATAGAGGACCCTCTTTAAGAACAACTACTGCCTCTGGAAAACTCATAACAGAATGGTGCTGCCGATCTTGCACATTACCACCGCTAAGATACAGAGGTGAGGACGGATGCTGGTACGGGATGGAAATCAGACCATTGAAAGAGAAAGAAGAGAATTTGGTCAACTCCTTGGTCACAGCCGGACATGGGCAGATTGACAACTTTTCACTAGGAGTCTTGGGAATGGCATTGTTCCTGGAAGAAATGCTCAGGACCCGAGTAGGAACGAAACATGCAATACTACTAGTTGCAGTTTCTTTTGTGACATTGATCACAGGGAACATGTCCTTTAGAGACCTGGGAAGAGTGATGGTTATGGTGGGCGCTACTATGACGGATGACATAGGTATGGGCGTGACTTATCTTGCCCTACTAGCAGCCTTCAAAGTCAGACCAACTTTTGCAGCCGGACTACTCTTGAGAAAGTTGACCTCCAAGGAATTGATGATGACTACCATAGGAATCGTACTCCTCTCCCAGAGCACCATACCAGAGACCATTCTTGAACTGACTGATGCGTTAGCCTTGGGCATGATGGTCCTTAAAATGGTGAGAAAAATGGAAAAGTATCAATTGGCAGTGACTATCATGGCTATCTTGTGCGTCCCAAATGCAGTGATATTACAAAACGCATGGAAAGTGAGTTGCACAATATTGGCAGTGGTGTCCGTTTCCCCACTGTTCTTAACATCCTCACAGCAGAAAGCGGATTGGATACCATTAGCATTGACGATCAAGGGTCTCAATCCAACAGCTATTTTTCTAACAACCCTTTCAAGAACCAACAAGAAAAGGAGCTGGCCACTAAATGAGGCTATCATGGCAGTCGGGATGGTGAGCATTTTGGCCAGTTCACTCCTAAAGAATGACATTCCCATGACAGGACCATTAGTGGCTGGAGGGCTCCTCACTGTGTGCTACGTGCTCACTGGACGATCGGCCGATTTGGAACTGGAGAGAGCCGCCGATGTCAAATGGGAAGATCAGGCAGAGATATCAGGAAGCAGTCCAATCCTGTCAATAACAATATCAGAAGATGGTAGCATGTCGATAAAAAACGAAGAGGAAGAACAAACACTGACCATACTCATTAGAACAGGATTGCTGGTGATCTCAGGACTTTTTCCTGTATCAATACCAATCACGGCAGCAGCATGGTACCTGTGGGAAGTGAAGAAACAACGGGCTGGAGTATTGTGGGATGTCCCTTCACCCCCACCCGTGGGAAAGGCTGAACTGGAAGATGGAGCCTATAGAATCAAGCAAAAAGGGATTCTTGGATATTCCCAGATCGGAGCCGGAGTTTACAAAGAAGGAACATTCCATACAATGTGGCATGTCACACGCGGCGCTGTTCTAATGCATAAAGGAAAGAGGATTGAACCATCATGGGCGGACGTTAAGAAAGACCTAATATCATATGGAGGAGGCTGGAAGCTAGAAGGAGAATGGAAGGAAGGAGAAGAAGTCCAGGTCTTGGCATTGGAGCCTGGAAAAAATCCAAGAGCCGTCCAAACAAAACCTGGTCTTTTCAAAACCAACGCCGGAACCATAGGTGCCGTATCTCTGGACTTTTCTCCTGGAACCTCAGGATCTCCAATCATCGACAAAAAAGGAAAAGTTGTGGGTCTTTATGGTAATGGTGTTGTTACAAGGAGTGGAGCATATGTGAGTGCTATAGCCCAGACTGAAAAAAGTATTGAAGACAATCCAGAGATCGAAGATGACATTTTTCGAAAGAGAAAATTGACCATCATGGACCTCCACCCAGGAGCGGGAAAGACGAAGAGATACCTTCCGGCCATAGTCAGAGAGGCTATAAAACGGGGCCTGAGGACATTAATCCTGGCCCCCACTAGAGTCGTGGCAGCTGAAATGGAGGAAGCCCTAAGAGGACTTCCAATAAGATACCAAACCCCAGCCATCAGAGCTGAGCACACCGGGCGGGAGATTGTGGACCTAATGTGTCATGCCACATTCACTATGAGGCTGCTATCACCAGTTAGAGTGCCAAATTACAACCTGATCATCATGGACGAAGCCCATTTCACAGACCCAGCAAGTATAGCGGCTAGAGGATACATCTCAACTCGAGTAGAGATGGGTGAGGCAGCTGGGATTTTCATGACAGCCACTCCTCCGGGAAGCAGAGACCCATTCCCTCAGAGCAATGCACCAATCATGGATGAAGAAAGAGAAATCCCTGAACGTTCGTGGAGTTCTGGACATGAGTGGGTCACGGATTTTAAAGGGAAGACTGTTTGGTTCGTTCCAAGTATAAAAGCAGGAAATGATATAGCAGCTTGCCTGAGAAAAAATGGAAAGAAAGTGATACAACTCAGTAGGAAGACCTTTGATTCTGAGTATGTCAAGACTAGAACCAATGATTGGGACTTCGTGGTCACAACTGACATTTCAGAAATGGGTGCCAACTTCAAGGCTGAGAGGGTTATAGACCCCAGACGCTGCATGAAACCAGTTATACTAACAGATGGTGAAGAGCGGGTGATCCTGGCAGGACCTATGCCAGTGACCCACTCTAGTGCAGCACAAAGAAGAGGGAGAATAGGAAGAAATCCAAAAAATGAAAATGACCAGTACATATACATGGGGGAACCTCTGGAAAATGATGAAGACTGTGCACACTGGAAAGAAGCTAAAATGCTCCTAGATAACATCAACACACCTGAAGGAATCATTCCTAGCATGTTCGAACCAGAGCGTGAAAAGGTGGATGCCATTGATGGTGAATACCGCTTGAGAGGAGAAGCAAGGAAAACCTTTGTGGACCTAATGAGAAGAGGAGACCTACCAGTCTGGTTGGCCTACAGAGTGGCAGCTGAAGGCATCAACTACGCAGACAGAAGGTGGTGTTTTGATGGAATTAAGAACAACCAAATCTTGGAAGAAAATGTGGAGGTGGAAATCTGGACAAAAGAAGGGGAAAGGAAGAAATTAAAACCCAGATGGTTGGATGCCAGGATCTACTCTGACCCACTGGCGCTAAAGGAATTCAAGGAGTTTGCAGCTGGAAGAAAGTCCCTGACCCTGAACCTAATCACAGAAATGGGTAGGCTTCCAACTTTCATGACTCAGAAGGCAAGAGACGCACTGGACAACTTAGCAGTGCTGCACACGGCTGAAGCAGGTGGAAGGGCGTACAATCATGCTCTCAGTGAACTGCCGGAGACCCTGGAGACATTGCTTTTACTGACACTTCTGGCTACAGTCACAGGAGGAATCTTTTTATTCTTGATGAGCGGAAGGGGTATAGGGAAGATGACCCTGGGAATGTGCTGCATAATCACGGCTAGTATTCTCCTATGGTACGCACAAATACAGCCACACTGGATAGCAGCTTCAATAATACTGGAGTTTTTTCTCATAGTTTTGCTTATTCCAGAACCAGAAAAGCAGAGAACACCCCAAGATAACCAATTGACCTACGTTGTCATAGCCATCCTCACAGTGGTGGCCGCAACCATGGCAAACGAGATGGGTTTCCTGGAAAAAACGAAGAAAGATCTCGGATTGGGAAGCATTACAACCCAGCAACCCGAGAGCAACATCCTGGACATAGATCTACGTCCCGCATCAGCATGGACGCTGTATGCTGTGGCCACAACATTTGTCACACCAATGTTGAGACACAGCATTGAAAATTCCTCAGTGAACGTGTCCCTAACAGCTATTGCCAACCAAGCCACAGTGTTAATGGGTCTTGGGAAAGGATGGCCATTGTCAAAGATGGACATCGGAGTTCCCCTTCTCGCCATTGGATGCTACTCACAAGTCAACCCCATAACTCTCACAGCAGCTCTTTTCTTACTGGTAGCACATTATGCCATCATAGGGCCAGGACTCCAAGCAAAAGCAACCAGAGAAGCTCAGAAAAGAGCAGCAGCGGGCATCATGAAAAACCCAACTGTCGATGGAATAACAGTGATTGACCTAGATCCAATACCCTATGATCCAAAGTTTGAAAAGCAGTTGGGACAAGTAATGCTCCTAGTCCTCTGCGTGACTCAAGTGTTGATGATGAGGACTACATGGGCTCTGTGTGAGGCTTTAACCTTAGCGACCGGGCCTATCTCCACATTGTGGGAAGGAAATCCAGGGAGGTTTTGGAACACTACCATTGCAGTGTCAATGGCTAACATTTTTAGAGGGAGTTACTTGGCCGGAGCTGGACTTCTCTTTTCCATCATGAAGAACACAACCAACACGAGAAGGGGAACTGGCAACATAGGAGAGACGCTTGGAGAGAAATGGAAAAGCCGATTGAACGCATTGGGGAAAAGTGAATTCCAGATCTACAAGAAAAGTGGAATCCAGGAAGTGGATAGAACCTTAGCAAAAGAAGGCATTAAAAGAGGAGAAACGGACCATCACGCTGTGTCGCGAGGCTCAGCAAAACTGAGATGGTTCGTCGAGAGAAATATGGTCACACCAGAAGGGAAAGTAGTGGACCTCGGTTGCGGCAGAGGAGGCTGGTCATACTATTGTGGGGGACTAAAGAATGTAAGAGAAGTCAAAGGCCTAACAAAAGGAGGACCAGGACATGAAGAACCCATCCCCATGTCAACATATGGGTGGAATCTAGTACGTCTTCAAAGTGGAGTTGACGTTTTCTTCACTCCGCCAGAAAAGTGTGACACATTGTTGTGTGACATAGGGGAGTCGTCACCAAATCCCACGGTAGAAGCAGGACGAACACTCAGAGTCCTTAACTTAGTGGAAAATTGGTTGAACAACAACACCCAATTTTGCATAAAGGTTCTCAACCCATACATGCCCTCAGTCATAGAAAAAATGGAAGCACTACAAAGGAAATATGGAGGAGCCTTAGTGAGGAATCCACTCTCACGAAACTCCACACATGAGATGTACTGGGTATCCAATGCCTCCGGGAACATAGTGTCATCAGTGAACATGATTTCAAGGATGTTGATCAACAGATTCACAATGAGACACAAGAAAGCCACTTACGAGCCAGATGTAGACCTCGGAAGCGGAACCCGCAACATCGGAATTGAAAGTGAGATACCAAACCTAGACATAATCGGGAAAAGAATAGAAAAAATAAAACAAGAGCATGAAACATCATGGCACTATGACCAAGACCACCCATACAAAACGTGGGCTTACCATGGCAGCTATGAAACAAAACAAACTGGATCAGCATCATCCATGGTGAACGGAGTGGTCAGACTGCTGACAAAACCTTGGGACGTCGTCCCCATGGTGACACAGATGGCAATGACAGACACGACTCCATTTGGACAACAGCGCGTTTTTAAAGAAAAAGTGGACACGAGAACCCAAGAACCGAAAGAAGGCACAAAGAAACTAATGAAAATCACGGCAGAGTGGCTTTGGAAAGAACTAGGGAAGAAAAAGACACCTAGGATGTGCACTAGAGAAGAATTCACAAGAAAGGTGAGAAGCAATGCAGCCTTGGGGGCCATATTCACTGATGAGAACAAGTGGAAGTCGGCACGTGAGGCTGTTGAAGATAGTAGGTTTTGGGAGCTGGTTGACAAGGAAAGGAATCTCCATCTTGAAGGAAAGTGTGAAACATGTGTGTATAACATGATGGGAAAAAGAGAGAAGAAGCTAGGGGAGTTCGGCAAGGCAAAAGGCAGCAGAGCCATATGGTACATGTGGCTTGGAGCACGCTTCTTAGAGTTTGAAGCCCTAGGATTCTTGAATGAAGATCACTGGTTCTCCAGAGAGAACTCCTTGAGTGGAGTGGAAGGAGAAGGGCTGCACAAGCTAGGTTACATTTTAAGAGACGTGAGCAAGAAAGAGGGAGGAGCAATGTATGCCGATGACACCGCAGGATGGGACACAAGAATCACACTAGAAGACCTAAAAAATGAAGAAATGGTAACAAACCACATGGAAGGAGAACACAAGAAACTAGCCGAGGCCATTTTCAAATTAACGTACCAAAACAAGGTGGTGCGTGTGCAAAGACCAACACCAAGAGGCACAGTAATGGATATCATATCGAGAAGAGACCAAAGAGGTAGTGGACAAGTTGGTACCTATGGACTCAATACTTTCACCAATATGGAAGCCCAACTAATCAGACAGATGGAGGGAGAAGGAGTCTTCAAAAGCATTCAGCACCTGACAGTCACAGAAGAAATCGCCGTGCAAAACTGGTTAGCAAGAGTAGGGCGCGAAAGGTTATCAAGAATGGCCATCAGTGGAGATGATTGTGTTGTGAAACCTTTAGATGACAGGTTCGCAAGCGCTTTAACAGCTCTAAATGACATGGGAAAGGTTAGGAAAGACATACAACAATGGGAACCTTCAAGAGGATGGAACGATTGGACACAAGTGCCCTTCTGTTCACACCATTTCCATGAGTTAATCATGAAAGACGGCCGCGTACTTGTAGTTCCATGCAGAAACCAAGATGAACTGATTGGTAGAGCCCGAATTTCCCAAGGAGCTGGGTGGTCTTTGCGAGAGACGGCCTGTTTGGGGAAGTCCTACGCCCAAATGTGGAGCTTGATGTACTTCCACAGACGTGACCTCAGGCTGGCGGCTAATGCTATTTGCTCGGCAGTCCCATCACATTGGGTTCCAACAAGTAGAACAACCTGGTCCATACACGCCAAACATGAATGGATGACAACGGAAGACATGCTGACAGTCTGGAACAGGGTGTGGATTCAAGAAAACCCATGGATGGAAGACAAAACTCCAGTGGAATCATGGGAGGAAATCCCATACTTGGGGAAAAGAGAAGACCAATGGTGCGGCTCATTGATTGGGCTAACAAGCAGGGCCACCTGGGCAAAGAACATCCAAACAGCAATAAATCAAGTTAGATCCCTTATAGGCTATGAGGAATACACAGATTACATGCCATCCATGAAAAGATTCAGAAGAGAAGAGGAAGAGGCAGGAGTCCTGTGGTAGAAGGCAAAACTAACATGAAACAAGGCTAGAAGTCAGGTCGGATTAAGCCATAGTACGGAAAAAACTATGCTACCTGTGAGCCCCGTCCAAGGACGTTAAAAGAAGTCAGGCCATTACAAATGCCATAGCTTGAGTAAACTGAGCAGCCTGTAGCTCCACCTGAGAAGGTGTAAAAAATCTGGGAGGCCACAAACCATGGAAGCTGTACGCATGGCGTAGTGGACTAGCGGTTAGAGGAGACCCCTCCCTTACAAATCGCAGCAACAATGGGGGCCCAAGGTGAGATGAAGCTGTAGTCTCACTGGAAGGACTAGAGGTTAGAGGAGACCCCCCCAAAACAAAAAACAGCATATTGACGCTGGGAAAGACCAGAGATCCTGCTGTCTCCTCAGCATCATTCCAGGCACAGAACGCCAGAAAATGGAATGGTGCTGTTGAATCAACAGGTTCT

**IRES-Rluc-IRES-E73(DENV4)-DENV2**

AGTTGTTAGTCTACGTGGACCGACAAAGACAGATTCTTTGAGGGAGCTAAGCTCAACGTAGTTCTAACAGTTTTTTAATTAGAGAGCAGATCTCTGATGAATAACCAACGAAAAAAGGCGAGAAATACGCCTTTCAATATGCTGAAACGCGAGAGAAACCGCGTGTCGACTGTACAACAGCTGACAAAGAGATTCTCACTTGGAATGCTGCAGGGACGAGGACCATTAAAACTGTTCATGGCCCTGGTGGCGTTCCTTCGTTTCCTAACAATCCCACCAACAGCAGGGATACTGAAGAGATGGGGAACAATTAAAAAATCAAAAGCCATTAATGTTTTGAGAGGGTTCAGGAAAGAGATTGGAAGGATGCTGAACATCTTGAACAGGAGACGCAGAACTGCAGGCATGATCATTATGCTGATTCCAACAGTGATGGCGTTCCATTTAACCACACGTAACGGAGAACCACACATGATCGTCAGTAGACAAGAGAAAGGGAAAAGTCTTCTGTTTAAAACAGAGGATGGTGTGAACATGTGTACCCTCATGGCCATGGACCTTGGTGAATTGTGTGAAGATACAATCACGTACAAGTGTCCTTTTCTCAAGCAGAATGAACCAGAAGACATAGATTGTTGGTGCAACTCTACGTCCACATGGGTAACTTATGGGACGTGTACCACCACAGGAGAACACAGAAGAGAAAAAAGATCAGTGGCACTCGTTCCACATGTGGGAATGGGACTGGAGACACGAACTGAAACATGGATGTCATCAGAAGGGGCCTGGAAACATGCCCAGAGAATTGAAACTTGGATCTTGAGACATCCAGGCTTTACCATAATGGCAGCAATCCTGGCATACACCATAGGAACGACACATTTCCAAAGAGCCCTGATTTTCATCTTACTGACAGCTGTCGCTCCTTCAATGACAATGCGTTGCATAGGAATATCAAATAGAGACTTTGTAGAAGGGGTTTCAGGAGGAAGCTGGGTTGACATAGTCTTAGAACATGGAAGCTGTGTGACGACGATGGCAAAAAACAAACCAACATTGGATTTTGAACTGATAAAAACAGAAGCCAAACAACCTGCCACTCTAAGGAAGTACTGTATAGAGGCAAAGCTGACCAACACAACAACAGATTCTCGCTGCCCAACACAAGGAGAACCCAGCCTAAATGAAGAGCAGGACAAAAGGTTCGTCTGCAAACACTCCATGGTGGACAGAGGATGGGGAAATGGATGTGGACTATTTGGAAAAGGAGGCATTGTGACCTGTGCTATGTTCACATGCAAAAAGAACATGAAAGGAAAAGTCGTGCAACCAGAAAACTTGGAATACACCATTGTGATAACACCTCACTCAGGGGAAGAGCATGCAGTCGGAAATGACACAGGAAAACATGGCAAGGAAATCAAAATAACACCACAGAGTTCCATCACAGAAGCAGAGTTGACAGGCTATGGCACTGTCACGATGGAGTGCTCTCCGAGAACGGGCCTCGACTTCAATGAGATGGTGTTGCTGCAAATGGAAAATAAAGCTTGGCTGGTGCACAGGCAATGGTTCCTAGACCTGCCGTTGCCATGGCTGCCCGGAGCGGACACACAAGGATCAAATTGGATACAGAAAGAGACATTGGTCACTTTCAAAAATCCCCATGCGAAGAAACAGGATGTTGTTGTTTTGGGATCCCAAGAAGGGGCCATGCACACAGCACTCACAGGGGCCACAGAAATCCAGATGTCATCAGGAAACTTACTGTTCACAGGACATCTCAAGTGCAGGCTGAGGATGGACAAACTACAGCTCAAAGGAATGTCATACTCTATGTGCACAGGAAAGTTTAAAGTTGTGAAGGAAATAGCAGAAACACAACATGGAACAATAGTTATCAGAGTACAATATGAAGGGGACGGTTCTCCATGTAAGATCCCTTTTGAGATAATGGATTTGGAAAAAAGACATGTTTTAGGTCGCCTGATTACAGTCAACCCAATCGTAACAGAAAAAGATAGCCCAGTCAACATAGAAGCAGAACCTCCATTCGGAGACAGCTACATCATCATAGGAGTAGAGCCGGGACAATTGAAGCTCAACTGGTTTAAGAAAGGAAGTTCTATCGGCCAAATGATTGAGACAACAATGAGGGGAGCGAAGAGAATGGCCATTTTAGGTGACACAGCTTGGGATTTTGGATCCCTGGGAGGAGTGTTTACATCTATAGGAAAGGCTCTCCACCAAGTTTTCGGAGCAATCTATGGGGCTGCCTTCAGTGGGGTCTCATGGATTATGAAAATCCTCATAGGAGTCATTATCACATGGATAGGAATGAATTCACGCAGCACCTCACTGTCTGTGTCACTAGTATTGGTGGGAGTCGTGACGCTGTATTTGGGAGTTATGGTGCAGGCCTAGGCGGCCGCCCCTCTCCCTCCCCCCCCCCTAACGTTACTGGCCGAAGCCGCTTGGAATAAGGCCGGTGTGCGTTTGTCTATATGTTATTTTCCACCATATTGCCGTCTTTTGGCAATGTGAGGGCCCGGAAACCTGGCCCTGTCTTCTTGACGAGCATTCCTAGGGGTCTTTCCCCTCTCGCCAAAGGAATGCAAGGTCTGTTGAATGTCGTGAAGGAAGCAGTTCCTCTGGAAGCTTCTTGAAGACAAACAACGTCTGTAGCGACCCTTTGCAGGCAGCGGAACCCCCCACCTGGCGACAGGTGCCTCTGCGGCCAAAAGCCACGTGTATAAGATACACCTGCAAAGGCGGCACAACCCCAGTGCCACGTTGTGAGTTGGATAGTTGTGGAAAGAGTCAAATGGCTCTCCTCAAGCGTATTCAACAAGGGGCTGAAGGATGCCCAGAAGGTACCCCATTGTATGGGATCTGATCTGGGGCCTCGGTGCACATGCTTTACATGTGTTTAGTCGAGGTTAAAAAAACGTCTAGGCCCCCCGAACCACGGGGACGTGGTTTTCCTTTGAAAAACACGATAATACCATGGCTTCCAAGGTGTACGACCCCGAGCAACGCAAACGCATGATCACTGGGCCTCAGTGGTGGGCTCGCTGCAAGCAAATGAACGTGCTGGACTCCTTCATCAACTACTATGATTCCGAGAAGCACGCCGAGAACGCCGTGATTTTTCTGCATGGTAACGCTGCCTCCAGCTACCTGTGGAGGCACGTCGTGCCTCACATCGAGCCCGTGGCTAGATGCATCATCCCTGATCTGATCGGAATGGGTAAGTCCGGCAAGAGCGGGAATGGCTCATATCGCCTCCTGGATCACTACAAGTACCTCACCGCTTGGTTCGAGCTGCTGAACCTTCCAAAGAAAATCATCTTTGTGGGCCACGACTGGGGGGCTTGTCTGGCCTTTCACTACTCCTACGAGCACCAAGACAAGATCAAGGCCATCGTCCATGCTGAGAGTGTCGTGGACGTGATCGAGTCCTGGGACGAGTGGCCTGACATCGAGGAGGATATCGCCCTGATCAAGAGCGAAGAGGGCGAGAAAATGGTGCTTGAGAATAACTTCTTCGTCGAGACCATGCTCCCAAGCAAGATCATGCGGAAACTGGAGCCTGAGGAGTTCGCTGCCTACCTGGAGCCATTCAAGGAGAAGGGCGAGGTTAGACGGCCTACCCTCTCCTGGCCTCGCGAGATCCCTCTCGTTAAGGGAGGCAAGCCCGACGTCGTCCAGATTGTCCGCAACTACAACGCCTACCTTCGGGCCAGCGACGATCTGCCTAAGATGTTCATCGAGTCCGACCCTGGGTTCTTTTCCAACGCTATTGTCGAGGGAGCTAAGAAGTTCCCTAACACCGAGTTCGTGAAGGTGAAGGGCCTCCACTTCAGCCAGGAGGACGCTCCAGATGAAATGGGTAAGTACACCAAGAGCTTCGTGGAGCGCGTGGTGAAGAACGAGCAGTAGGCGGCCGCCCCTCTCCCTCCCCCCCCCCTAACGTTACTGGCCGAAGCCGCTTGGAATAAGGCCGGTGTGCGTTTGTCTATATGTTATTTTCCACCATATTGCCGTCTTTTGGCAATGTGAGGGCCCGGAAACCTGGCCCTGTCTTCTTGACGAGCATTCCTAGGGGTCTTTCCCCTCTCGCCAAAGGAATGCAAGGTCTGTTGAATGTCGTGAAGGAAGCAGTTCCTCTGGAAGCTTCTTGAAGACAAACAACGTCTGTAGCGACCCTTTGCAGGCAGCGGAACCCCCCACCTGGCGACAGGTGCCTCTGCGGCCAAAAGCCACGTGTATAAGATACACCTGCAAAGGCGGCACAACCCCAGTGCCACGTTGTGAGTTGGATAGTTGTGGAAAGAGTCAAATGGCTCTCCTCAAGCGTATTCAACAAGGGGCTGAAGGATGCCCAGAAGGTACCCCATTGTATGGGATCTGATCTGGGGCCTCGGTGCACATGCTTTACATGTGTTTAGTCGAGGTTAAAAAAACGTCTAGGCCCCCCGAACCACGGGGACGTGGTTTTCCTTTGAAAAACACGATAATACCATGGGTTCCGTTGGTGGACTGTTCACATCATTGGGAAAGGCTGTGCACCAGGTTTTTGGAAGTGTGTATACAACCATGTTTGGAGGAGTCTCATGGATGATTAGAATCCTAATTGGGTTCTTAGTGTTGTGGATTGGCACGAACTCAAGGAACACTTCAATGGCTATGACGTGCATAGCTGTTGGAGGAATCACTCTGTTTCTGGGCTTCACAGTTCAAGCAGATAGTGGTTGCGTTGTGAGCTGGAAAAACAAAGAACTGAAGTGTGGCAGTGGGATTTTCATCACAGACAACGTGCACACATGGACAGAACAATACAAGTTCCAACCAGAATCCCCTTCAAAACTAGCTTCAGCTATCCAGAAAGCTCATGAAGAGGGCATTTGTGGAATCCGCTCAGTAACAAGACTGGAAAATCTGATGTGGAAACAAATAACACCAGAATTGAATCACATTCTATCAGAAAATGAGGTGAAGTTGACTATTATGACAGGAGACATCAAAGGAATCATGCAGGCAGGAAAACGATCTCTGCAGCCCCAGCCCACTGAGCTGAAGTATTCATGGAAAACATGGGGCAAAGCGAAAATGCTCTCTACAGAGTCTCATAACCAGACCTTTCTCATTGATGGCCCCGAAACAGCAGAATGCCCCAACACAAACAGAGCTTGGAATTCGCTGGAAGTTGAAGACTATGGCTTTGGAGTATTCACCACCAATATATGGCTAAAGTTGAGAGAAAAGCAGGATGTATTCTGCGACTCAAAACTCATGTCAGCGGCCATAAAAGACAACAGAGCCGTCCATGCCGATATGGGTTATTGGATAGAAAGTGCACTCAATGACACATGGAAGATAGAGAAAGCCTCTTTCATCGAAGTTAAAAGCTGCCACTGGCCAAAGTCACACACCCTCTGGAGTAATGGAGTGTTAGAAAGTGAGATGATAATTCCAAAGAATTTCGCTGGACCAGTGTCACAACACAACTACAGACCAGGTTACCATACACAAACAGCAGGACCATGGCATCTAGGTAAGCTTGAGATGGACTTTGATTTCTGCGAAGGAACCACAGTGGTGGTGACTGAGGACTGTGGAAATAGAGGACCCTCTTTAAGAACAACTACTGCCTCTGGAAAACTCATAACAGAATGGTGCTGCCGATCTTGCACATTACCACCGCTAAGATACAGAGGTGAGGACGGATGCTGGTACGGGATGGAAATCAGACCATTGAAAGAGAAAGAAGAGAATTTGGTCAACTCCTTGGTCACAGCCGGACATGGGCAGATTGACAACTTTTCACTAGGAGTCTTGGGAATGGCATTGTTCCTGGAAGAAATGCTCAGGACCCGAGTAGGAACGAAACATGCAATACTACTAGTTGCAGTTTCTTTTGTGACATTGATCACAGGGAACATGTCCTTTAGAGACCTGGGAAGAGTGATGGTTATGGTGGGCGCTACTATGACGGATGACATAGGTATGGGCGTGACTTATCTTGCCCTACTAGCAGCCTTCAAAGTCAGACCAACTTTTGCAGCCGGACTACTCTTGAGAAAGTTGACCTCCAAGGAATTGATGATGACTACCATAGGAATCGTACTCCTCTCCCAGAGCACCATACCAGAGACCATTCTTGAACTGACTGATGCGTTAGCCTTGGGCATGATGGTCCTTAAAATGGTGAGAAAAATGGAAAAGTATCAATTGGCAGTGACTATCATGGCTATCTTGTGCGTCCCAAATGCAGTGATATTACAAAACGCATGGAAAGTGAGTTGCACAATATTGGCAGTGGTGTCCGTTTCCCCACTGTTCTTAACATCCTCACAGCAGAAAGCGGATTGGATACCATTAGCATTGACGATCAAGGGTCTCAATCCAACAGCTATTTTTCTAACAACCCTTTCAAGAACCAACAAGAAAAGGAGCTGGCCACTAAATGAGGCTATCATGGCAGTCGGGATGGTGAGCATTTTGGCCAGTTCACTCCTAAAGAATGACATTCCCATGACAGGACCATTAGTGGCTGGAGGGCTCCTCACTGTGTGCTACGTGCTCACTGGACGATCGGCCGATTTGGAACTGGAGAGAGCCGCCGATGTCAAATGGGAAGATCAGGCAGAGATATCAGGAAGCAGTCCAATCCTGTCAATAACAATATCAGAAGATGGTAGCATGTCGATAAAAAACGAAGAGGAAGAACAAACACTGACCATACTCATTAGAACAGGATTGCTGGTGATCTCAGGACTTTTTCCTGTATCAATACCAATCACGGCAGCAGCATGGTACCTGTGGGAAGTGAAGAAACAACGGGCTGGAGTATTGTGGGATGTCCCTTCACCCCCACCCGTGGGAAAGGCTGAACTGGAAGATGGAGCCTATAGAATCAAGCAAAAAGGGATTCTTGGATATTCCCAGATCGGAGCCGGAGTTTACAAAGAAGGAACATTCCATACAATGTGGCATGTCACACGCGGCGCTGTTCTAATGCATAAAGGAAAGAGGATTGAACCATCATGGGCGGACGTTAAGAAAGACCTAATATCATATGGAGGAGGCTGGAAGCTAGAAGGAGAATGGAAGGAAGGAGAAGAAGTCCAGGTCTTGGCATTGGAGCCTGGAAAAAATCCAAGAGCCGTCCAAACAAAACCTGGTCTTTTCAAAACCAACGCCGGAACCATAGGTGCCGTATCTCTGGACTTTTCTCCTGGAACCTCAGGATCTCCAATCATCGACAAAAAAGGAAAAGTTGTGGGTCTTTATGGTAATGGTGTTGTTACAAGGAGTGGAGCATATGTGAGTGCTATAGCCCAGACTGAAAAAAGTATTGAAGACAATCCAGAGATCGAAGATGACATTTTTCGAAAGAGAAAATTGACCATCATGGACCTCCACCCAGGAGCGGGAAAGACGAAGAGATACCTTCCGGCCATAGTCAGAGAGGCTATAAAACGGGGCCTGAGGACATTAATCCTGGCCCCCACTAGAGTCGTGGCAGCTGAAATGGAGGAAGCCCTAAGAGGACTTCCAATAAGATACCAAACCCCAGCCATCAGAGCTGAGCACACCGGGCGGGAGATTGTGGACCTAATGTGTCATGCCACATTCACTATGAGGCTGCTATCACCAGTTAGAGTGCCAAATTACAACCTGATCATCATGGACGAAGCCCATTTCACAGACCCAGCAAGTATAGCGGCTAGAGGATACATCTCAACTCGAGTAGAGATGGGTGAGGCAGCTGGGATTTTCATGACAGCCACTCCTCCGGGAAGCAGAGACCCATTCCCTCAGAGCAATGCACCAATCATGGATGAAGAAAGAGAAATCCCTGAACGTTCGTGGAGTTCTGGACATGAGTGGGTCACGGATTTTAAAGGGAAGACTGTTTGGTTCGTTCCAAGTATAAAAGCAGGAAATGATATAGCAGCTTGCCTGAGAAAAAATGGAAAGAAAGTGATACAACTCAGTAGGAAGACCTTTGATTCTGAGTATGTCAAGACTAGAACCAATGATTGGGACTTCGTGGTCACAACTGACATTTCAGAAATGGGTGCCAACTTCAAGGCTGAGAGGGTTATAGACCCCAGACGCTGCATGAAACCAGTTATACTAACAGATGGTGAAGAGCGGGTGATCCTGGCAGGACCTATGCCAGTGACCCACTCTAGTGCAGCACAAAGAAGAGGGAGAATAGGAAGAAATCCAAAAAATGAAAATGACCAGTACATATACATGGGGGAACCTCTGGAAAATGATGAAGACTGTGCACACTGGAAAGAAGCTAAAATGCTCCTAGATAACATCAACACACCTGAAGGAATCATTCCTAGCATGTTCGAACCAGAGCGTGAAAAGGTGGATGCCATTGATGGTGAATACCGCTTGAGAGGAGAAGCAAGGAAAACCTTTGTGGACCTAATGAGAAGAGGAGACCTACCAGTCTGGTTGGCCTACAGAGTGGCAGCTGAAGGCATCAACTACGCAGACAGAAGGTGGTGTTTTGATGGAATTAAGAACAACCAAATCTTGGAAGAAAATGTGGAGGTGGAAATCTGGACAAAAGAAGGGGAAAGGAAGAAATTAAAACCCAGATGGTTGGATGCCAGGATCTACTCTGACCCACTGGCGCTAAAGGAATTCAAGGAGTTTGCAGCTGGAAGAAAGTCCCTGACCCTGAACCTAATCACAGAAATGGGTAGGCTTCCAACTTTCATGACTCAGAAGGCAAGAGACGCACTGGACAACTTAGCAGTGCTGCACACGGCTGAAGCAGGTGGAAGGGCGTACAATCATGCTCTCAGTGAACTGCCGGAGACCCTGGAGACATTGCTTTTACTGACACTTCTGGCTACAGTCACAGGAGGAATCTTTTTATTCTTGATGAGCGGAAGGGGTATAGGGAAGATGACCCTGGGAATGTGCTGCATAATCACGGCTAGTATTCTCCTATGGTACGCACAAATACAGCCACACTGGATAGCAGCTTCAATAATACTGGAGTTTTTTCTCATAGTTTTGCTTATTCCAGAACCAGAAAAGCAGAGAACACCCCAAGATAACCAATTGACCTACGTTGTCATAGCCATCCTCACAGTGGTGGCCGCAACCATGGCAAACGAGATGGGTTTCCTGGAAAAAACGAAGAAAGATCTCGGATTGGGAAGCATTACAACCCAGCAACCCGAGAGCAACATCCTGGACATAGATCTACGTCCCGCATCAGCATGGACGCTGTATGCTGTGGCCACAACATTTGTCACACCAATGTTGAGACACAGCATTGAAAATTCCTCAGTGAACGTGTCCCTAACAGCTATTGCCAACCAAGCCACAGTGTTAATGGGTCTTGGGAAAGGATGGCCATTGTCAAAGATGGACATCGGAGTTCCCCTTCTCGCCATTGGATGCTACTCACAAGTCAACCCCATAACTCTCACAGCAGCTCTTTTCTTACTGGTAGCACATTATGCCATCATAGGGCCAGGACTCCAAGCAAAAGCAACCAGAGAAGCTCAGAAAAGAGCAGCAGCGGGCATCATGAAAAACCCAACTGTCGATGGAATAACAGTGATTGACCTAGATCCAATACCCTATGATCCAAAGTTTGAAAAGCAGTTGGGACAAGTAATGCTCCTAGTCCTCTGCGTGACTCAAGTGTTGATGATGAGGACTACATGGGCTCTGTGTGAGGCTTTAACCTTAGCGACCGGGCCTATCTCCACATTGTGGGAAGGAAATCCAGGGAGGTTTTGGAACACTACCATTGCAGTGTCAATGGCTAACATTTTTAGAGGGAGTTACTTGGCCGGAGCTGGACTTCTCTTTTCCATCATGAAGAACACAACCAACACGAGAAGGGGAACTGGCAACATAGGAGAGACGCTTGGAGAGAAATGGAAAAGCCGATTGAACGCATTGGGGAAAAGTGAATTCCAGATCTACAAGAAAAGTGGAATCCAGGAAGTGGATAGAACCTTAGCAAAAGAAGGCATTAAAAGAGGAGAAACGGACCATCACGCTGTGTCGCGAGGCTCAGCAAAACTGAGATGGTTCGTCGAGAGAAATATGGTCACACCAGAAGGGAAAGTAGTGGACCTCGGTTGCGGCAGAGGAGGCTGGTCATACTATTGTGGGGGACTAAAGAATGTAAGAGAAGTCAAAGGCCTAACAAAAGGAGGACCAGGACATGAAGAACCCATCCCCATGTCAACATATGGGTGGAATCTAGTACGTCTTCAAAGTGGAGTTGACGTTTTCTTCACTCCGCCAGAAAAGTGTGACACATTGTTGTGTGACATAGGGGAGTCGTCACCAAATCCCACGGTAGAAGCAGGACGAACACTCAGAGTCCTTAACTTAGTGGAAAATTGGTTGAACAACAACACCCAATTTTGCATAAAGGTTCTCAACCCATACATGCCCTCAGTCATAGAAAAAATGGAAGCACTACAAAGGAAATATGGAGGAGCCTTAGTGAGGAATCCACTCTCACGAAACTCCACACATGAGATGTACTGGGTATCCAATGCCTCCGGGAACATAGTGTCATCAGTGAACATGATTTCAAGGATGTTGATCAACAGATTCACAATGAGACACAAGAAAGCCACTTACGAGCCAGATGTAGACCTCGGAAGCGGAACCCGCAACATCGGAATTGAAAGTGAGATACCAAACCTAGACATAATCGGGAAAAGAATAGAAAAAATAAAACAAGAGCATGAAACATCATGGCACTATGACCAAGACCACCCATACAAAACGTGGGCTTACCATGGCAGCTATGAAACAAAACAAACTGGATCAGCATCATCCATGGTGAACGGAGTGGTCAGACTGCTGACAAAACCTTGGGACGTCGTCCCCATGGTGACACAGATGGCAATGACAGACACGACTCCATTTGGACAACAGCGCGTTTTTAAAGAAAAAGTGGACACGAGAACCCAAGAACCGAAAGAAGGCACAAAGAAACTAATGAAAATCACGGCAGAGTGGCTTTGGAAAGAACTAGGGAAGAAAAAGACACCTAGGATGTGCACTAGAGAAGAATTCACAAGAAAGGTGAGAAGCAATGCAGCCTTGGGGGCCATATTCACTGATGAGAACAAGTGGAAGTCGGCACGTGAGGCTGTTGAAGATAGTAGGTTTTGGGAGCTGGTTGACAAGGAAAGGAATCTCCATCTTGAAGGAAAGTGTGAAACATGTGTGTATAACATGATGGGAAAAAGAGAGAAGAAGCTAGGGGAGTTCGGCAAGGCAAAAGGCAGCAGAGCCATATGGTACATGTGGCTTGGAGCACGCTTCTTAGAGTTTGAAGCCCTAGGATTCTTGAATGAAGATCACTGGTTCTCCAGAGAGAACTCCTTGAGTGGAGTGGAAGGAGAAGGGCTGCACAAGCTAGGTTACATTTTAAGAGACGTGAGCAAGAAAGAGGGAGGAGCAATGTATGCCGATGACACCGCAGGATGGGACACAAGAATCACACTAGAAGACCTAAAAAATGAAGAAATGGTAACAAACCACATGGAAGGAGAACACAAGAAACTAGCCGAGGCCATTTTCAAATTAACGTACCAAAACAAGGTGGTGCGTGTGCAAAGACCAACACCAAGAGGCACAGTAATGGATATCATATCGAGAAGAGACCAAAGAGGTAGTGGACAAGTTGGTACCTATGGACTCAATACTTTCACCAATATGGAAGCCCAACTAATCAGACAGATGGAGGGAGAAGGAGTCTTCAAAAGCATTCAGCACCTGACAGTCACAGAAGAAATCGCCGTGCAAAACTGGTTAGCAAGAGTAGGGCGCGAAAGGTTATCAAGAATGGCCATCAGTGGAGATGATTGTGTTGTGAAACCTTTAGATGACAGGTTCGCAAGCGCTTTAACAGCTCTAAATGACATGGGAAAGGTTAGGAAAGACATACAACAATGGGAACCTTCAAGAGGATGGAACGATTGGACACAAGTGCCCTTCTGTTCACACCATTTCCATGAGTTAATCATGAAAGACGGCCGCGTACTTGTAGTTCCATGCAGAAACCAAGATGAACTGATTGGTAGAGCCCGAATTTCCCAAGGAGCTGGGTGGTCTTTGCGAGAGACGGCCTGTTTGGGGAAGTCCTACGCCCAAATGTGGAGCTTGATGTACTTCCACAGACGTGACCTCAGGCTGGCGGCTAATGCTATTTGCTCGGCAGTCCCATCACATTGGGTTCCAACAAGTAGAACAACCTGGTCCATACACGCCAAACATGAATGGATGACAACGGAAGACATGCTGACAGTCTGGAACAGGGTGTGGATTCAAGAAAACCCATGGATGGAAGACAAAACTCCAGTGGAATCATGGGAGGAAATCCCATACTTGGGGAAAAGAGAAGACCAATGGTGCGGCTCATTGATTGGGCTAACAAGCAGGGCCACCTGGGCAAAGAACATCCAAACAGCAATAAATCAAGTTAGATCCCTTATAGGCTATGAGGAATACACAGATTACATGCCATCCATGAAAAGATTCAGAAGAGAAGAGGAAGAGGCAGGAGTCCTGTGGTAGAAGGCAAAACTAACATGAAACAAGGCTAGAAGTCAGGTCGGATTAAGCCATAGTACGGAAAAAACTATGCTACCTGTGAGCCCCGTCCAAGGACGTTAAAAGAAGTCAGGCCATTACAAATGCCATAGCTTGAGTAAACTGAGCAGCCTGTAGCTCCACCTGAGAAGGTGTAAAAAATCTGGGAGGCCACAAACCATGGAAGCTGTACGCATGGCGTAGTGGACTAGCGGTTAGAGGAGACCCCTCCCTTACAAATCGCAGCAACAATGGGGGCCCAAGGTGAGATGAAGCTGTAGTCTCACTGGAAGGACTAGAGGTTAGAGGAGACCCCCCCAAAACAAAAAACAGCATATTGACGCTGGGAAAGACCAGAGATCCTGCTGTCTCCTCAGCATCATTCCAGGCACAGAACGCCAGAAAATGGAATGGTGCTGTTGAATCAACAGGTTCT

**IRES-TP53-IRES--E73(DENV4)-DENV2**

AGTTGTTAGTCTACGTGGACCGACAAAGACAGATTCTTTGAGGGAGCTAAGCTCAACGTAGTTCTAACAGTTTTTTAATTAGAGAGCAGATCTCTGATGAATAACCAACGAAAAAAGGCGAGAAATACGCCTTTCAATATGCTGAAACGCGAGAGAAACCGCGTGTCGACTGTACAACAGCTGACAAAGAGATTCTCACTTGGAATGCTGCAGGGACGAGGACCATTAAAACTGTTCATGGCCCTGGTGGCGTTCCTTCGTTTCCTAACAATCCCACCAACAGCAGGGATACTGAAGAGATGGGGAACAATTAAAAAATCAAAAGCCATTAATGTTTTGAGAGGGTTCAGGAAAGAGATTGGAAGGATGCTGAACATCTTGAACAGGAGACGCAGAACTGCAGGCATGATCATTATGCTGATTCCAACAGTGATGGCGTTCCATTTAACCACACGTAACGGAGAACCACACATGATCGTCAGTAGACAAGAGAAAGGGAAAAGTCTTCTGTTTAAAACAGAGGATGGTGTGAACATGTGTACCCTCATGGCCATGGACCTTGGTGAATTGTGTGAAGATACAATCACGTACAAGTGTCCTTTTCTCAAGCAGAATGAACCAGAAGACATAGATTGTTGGTGCAACTCTACGTCCACATGGGTAACTTATGGGACGTGTACCACCACAGGAGAACACAGAAGAGAAAAAAGATCAGTGGCACTCGTTCCACATGTGGGAATGGGACTGGAGACACGAACTGAAACATGGATGTCATCAGAAGGGGCCTGGAAACATGCCCAGAGAATTGAAACTTGGATCTTGAGACATCCAGGCTTTACCATAATGGCAGCAATCCTGGCATACACCATAGGAACGACACATTTCCAAAGAGCCCTGATTTTCATCTTACTGACAGCTGTCGCTCCTTCAATGACAATGCGTTGCATAGGAATATCAAATAGAGACTTTGTAGAAGGGGTTTCAGGAGGAAGCTGGGTTGACATAGTCTTAGAACATGGAAGCTGTGTGACGACGATGGCAAAAAACAAACCAACATTGGATTTTGAACTGATAAAAACAGAAGCCAAACAACCTGCCACTCTAAGGAAGTACTGTATAGAGGCAAAGCTGACCAACACAACAACAGATTCTCGCTGCCCAACACAAGGAGAACCCAGCCTAAATGAAGAGCAGGACAAAAGGTTCGTCTGCAAACACTCCATGGTGGACAGAGGATGGGGAAATGGATGTGGACTATTTGGAAAAGGAGGCATTGTGACCTGTGCTATGTTCACATGCAAAAAGAACATGAAAGGAAAAGTCGTGCAACCAGAAAACTTGGAATACACCATTGTGATAACACCTCACTCAGGGGAAGAGCATGCAGTCGGAAATGACACAGGAAAACATGGCAAGGAAATCAAAATAACACCACAGAGTTCCATCACAGAAGCAGAGTTGACAGGCTATGGCACTGTCACGATGGAGTGCTCTCCGAGAACGGGCCTCGACTTCAATGAGATGGTGTTGCTGCAAATGGAAAATAAAGCTTGGCTGGTGCACAGGCAATGGTTCCTAGACCTGCCGTTGCCATGGCTGCCCGGAGCGGACACACAAGGATCAAATTGGATACAGAAAGAGACATTGGTCACTTTCAAAAATCCCCATGCGAAGAAACAGGATGTTGTTGTTTTGGGATCCCAAGAAGGGGCCATGCACACAGCACTCACAGGGGCCACAGAAATCCAGATGTCATCAGGAAACTTACTGTTCACAGGACATCTCAAGTGCAGGCTGAGGATGGACAAACTACAGCTCAAAGGAATGTCATACTCTATGTGCACAGGAAAGTTTAAAGTTGTGAAGGAAATAGCAGAAACACAACATGGAACAATAGTTATCAGAGTACAATATGAAGGGGACGGTTCTCCATGTAAGATCCCTTTTGAGATAATGGATTTGGAAAAAAGACATGTTTTAGGTCGCCTGATTACAGTCAACCCAATCGTAACAGAAAAAGATAGCCCAGTCAACATAGAAGCAGAACCTCCATTCGGAGACAGCTACATCATCATAGGAGTAGAGCCGGGACAATTGAAGCTCAACTGGTTTAAGAAAGGAAGTTCTATCGGCCAAATGATTGAGACAACAATGAGGGGAGCGAAGAGAATGGCCATTTTAGGTGACACAGCTTGGGATTTTGGATCCCTGGGAGGAGTGTTTACATCTATAGGAAAGGCTCTCCACCAAGTTTTCGGAGCAATCTATGGGGCTGCCTTCAGTGGGGTCTCATGGATTATGAAAATCCTCATAGGAGTCATTATCACATGGATAGGAATGAATTCACGCAGCACCTCACTGTCTGTGTCACTAGTATTGGTGGGAGTCGTGACGCTGTATTTGGGAGTTATGGTGCAGGCCTAGGCGGCCGCCCCTCTCCCTCCCCCCCCCCTAACGTTACTGGCCGAAGCCGCTTGGAATAAGGCCGGTGTGCGTTTGTCTATATGTTATTTTCCACCATATTGCCGTCTTTTGGCAATGTGAGGGCCCGGAAACCTGGCCCTGTCTTCTTGACGAGCATTCCTAGGGGTCTTTCCCCTCTCGCCAAAGGAATGCAAGGTCTGTTGAATGTCGTGAAGGAAGCAGTTCCTCTGGAAGCTTCTTGAAGACAAACAACGTCTGTAGCGACCCTTTGCAGGCAGCGGAACCCCCCACCTGGCGACAGGTGCCTCTGCGGCCAAAAGCCACGTGTATAAGATACACCTGCAAAGGCGGCACAACCCCAGTGCCACGTTGTGAGTTGGATAGTTGTGGAAAGAGTCAAATGGCTCTCCTCAAGCGTATTCAACAAGGGGCTGAAGGATGCCCAGAAGGTACCCCATTGTATGGGATCTGATTCTGGGGCCTCGGTGCACATGCTTTACATGTGTTTAGTCGAGGTTAAAAAAACGTCTAGGCCCCCCGAACCACGGGGACGTGGTTTTCCTTTGAAAAACACGATAATACCATGGAGGAGCCGCAGTCAGATCCTAGCGTCGAGCCCCCTCTGAGTCAGGAAACATTTTCAGACCTATGGAAACTACTTCCTGAAAACAACGTTCTGTCCCCCTTGCCGTCCCAAGCAATGGATGATTTGATGCTGTCCCCGGACGATATTGAACAATGGTTCACTGAAGACCCAGGTCCAGATGAAGCTCCCAGAATGCCAGAGGCTGCTCCCCGCGTGGCCCCTGCACCAGCAGCTCCTACACCGGCGGCCCCTGCACCAGCCCCCTCCTGGCCCCTGTCATCTTCTGTCCCTTCCCAGAAAACCTACCAGGGCAGCTACGGTTTCCGTCTGGGCTTCTTGCATTCTGGGACAGCCAAGTCTGTGACTTGCACGTACTCCCCTGCCCTCAACAAGATGTTTTGCCAACTGGCCAAGACCTGCCCTGTGCAGCTGTGGGTTGATTCCACACCCCCGCCCGGCACCCGCGTCCGCGCCATGGCCATCTACAAGCAGTCACAGCACATGACGGAGGTTGTGAGGCGCTGCCCCCACCATGAGCGCTGCTCAGATAGCGATGGTCTGGCCCCTCCTCAGCATCTTATCCGAGTGGAAGGAAATCTGCGTGTGGAGTATTTGGATGACAGAAACACTTTTCGACATAGTGTGGTGGTGCCCTATGAGCCGCCTGAGGTTGGCTCTGACTGTACCACCATCCACTACAACTACATGTGTAACAGTTCCTGCATGGGCGGCATGAACCGGAGGCCCATCCTCACCATCATCACACTGGAAGACTCCAGTGGTAATCTACTGGGACGGAACAGCTTTGAGGTGCGTGTTTGTGCCTGTCCTGGGAGAGACCGGCGCACAGAGGAAGAGAATCTCCGCAAGAAAGGGGAGCCTCACCACGAGCTGCCCCCAGGGAGCACTAAGCGAGCACTGCCCAACAACACCAGCTCCTCTCCCCAGCCAAAGAAGAAACCACTGGATGGAGAATATTTCACCCTTCAGATCCGTGGGCGTGAGCGCTTCGAGATGTTCCGAGAGCTGAATGAGGCCTTGGAACTCAAGGATGCCCAGGCTGGGAAGGAGCCAGGGGGGAGCAGGGCTCACTCCAGCCACCTGAAGTCCAAAAAGGGTCAGTCTACCTCCCGCCATAAAAAACTCATGTTCAAGACAGAAGGGCCTGACTCAGACTGAGCGGCCGCCCCTCTCCCTCCCCCCCCCCTAACGTTACTGGCCGAAGCCGCTTGGAATAAGGCCGGTGTGCGTTTGTCTATATGTTATTTTCCACCATATTGCCGTCTTTTGGCAATGTGAGGGCCCGGAAACCTGGCCCTGTCTTCTTGACGAGCATTCCTAGGGGTCTTTCCCCTCTCGCCAAAGGAATGCAAGGTCTGTTGAATGTCGTGAAGGAAGCAGTTCCTCTGGAAGCTTCTTGAAGACAAACAACGTCTGTAGCGACCCTTTGCAGGCAGCGGAACCCCCCACCTGGCGACAGGTGCCTCTGCGGCCAAAAGCCACGTGTATAAGATACACCTGCAAAGGCGGCACAACCCCAGTGCCACGTTGTGAGTTGGATAGTTGTGGAAAGAGTCAAATGGCTCTCCTCAAGCGTATTCAACAAGGGGCTGAAGGATGCCCAGAAGGTACCCCATTGTATGGGATCTGATCTGGGGCCTCGGTGCACATGCTTTACATGTGTTTAGTCGAGGTTAAAAAAACGTCTAGGCCCCCCGAACCACGGGGACGTGGTTTTCCTTTGAAAAACACGATAATACCATGGGTTCCGTTGGTGGACTGTTCACATCATTGGGAAAGGCTGTGCACCAGGTTTTTGGAAGTGTGTATACAACCATGTTTGGAGGAGTCTCATGGATGATTAGAATCCTAATTGGGTTCTTAGTGTTGTGGATTGGCACGAACTCAAGGAACACTTCAATGGCTATGACGTGCATAGCTGTTGGAGGAATCACTCTGTTTCTGGGCTTCACAGTTCAAGCAGATAGTGGTTGCGTTGTGAGCTGGAAAAACAAAGAACTGAAGTGTGGCAGTGGGATTTTCATCACAGACAACGTGCACACATGGACAGAACAATACAAGTTCCAACCAGAATCCCCTTCAAAACTAGCTTCAGCTATCCAGAAAGCTCATGAAGAGGGCATTTGTGGAATCCGCTCAGTAACAAGACTGGAAAATCTGATGTGGAAACAAATAACACCAGAATTGAATCACATTCTATCAGAAAATGAGGTGAAGTTGACTATTATGACAGGAGACATCAAAGGAATCATGCAGGCAGGAAAACGATCTCTGCAGCCCCAGCCCACTGAGCTGAAGTATTCATGGAAAACATGGGGCAAAGCGAAAATGCTCTCTACAGAGTCTCATAACCAGACCTTTCTCATTGATGGCCCCGAAACAGCAGAATGCCCCAACACAAACAGAGCTTGGAATTCGCTGGAAGTTGAAGACTATGGCTTTGGAGTATTCACCACCAATATATGGCTAAAGTTGAGAGAAAAGCAGGATGTATTCTGCGACTCAAAACTCATGTCAGCGGCCATAAAAGACAACAGAGCCGTCCATGCCGATATGGGTTATTGGATAGAAAGTGCACTCAATGACACATGGAAGATAGAGAAAGCCTCTTTCATCGAAGTTAAAAGCTGCCACTGGCCAAAGTCACACACCCTCTGGAGTAATGGAGTGTTAGAAAGTGAGATGATAATTCCAAAGAATTTCGCTGGACCAGTGTCACAACACAACTACAGACCAGGTTACCATACACAAACAGCAGGACCATGGCATCTAGGTAAGCTTGAGATGGACTTTGATTTCTGCGAAGGAACCACAGTGGTGGTGACTGAGGACTGTGGAAATAGAGGACCCTCTTTAAGAACAACTACTGCCTCTGGAAAACTCATAACAGAATGGTGCTGCCGATCTTGCACATTACCACCGCTAAGATACAGAGGTGAGGACGGATGCTGGTACGGGATGGAAATCAGACCATTGAAAGAGAAAGAAGAGAATTTGGTCAACTCCTTGGTCACAGCCGGACATGGGCAGATTGACAACTTTTCACTAGGAGTCTTGGGAATGGCATTGTTCCTGGAAGAAATGCTCAGGACCCGAGTAGGAACGAAACATGCAATACTACTAGTTGCAGTTTCTTTTGTGACATTGATCACAGGGAACATGTCCTTTAGAGACCTGGGAAGAGTGATGGTTATGGTGGGCGCTACTATGACGGATGACATAGGTATGGGCGTGACTTATCTTGCCCTACTAGCAGCCTTCAAAGTCAGACCAACTTTTGCAGCCGGACTACTCTTGAGAAAGTTGACCTCCAAGGAATTGATGATGACTACCATAGGAATCGTACTCCTCTCCCAGAGCACCATACCAGAGACCATTCTTGAACTGACTGATGCGTTAGCCTTGGGCATGATGGTCCTTAAAATGGTGAGAAAAATGGAAAAGTATCAATTGGCAGTGACTATCATGGCTATCTTGTGCGTCCCAAATGCAGTGATATTACAAAACGCATGGAAAGTGAGTTGCACAATATTGGCAGTGGTGTCCGTTTCCCCACTGTTCTTAACATCCTCACAGCAGAAAGCGGATTGGATACCATTAGCATTGACGATCAAGGGTCTCAATCCAACAGCTATTTTTCTAACAACCCTTTCAAGAACCAACAAGAAAAGGAGCTGGCCACTAAATGAGGCTATCATGGCAGTCGGGATGGTGAGCATTTTGGCCAGTTCACTCCTAAAGAATGACATTCCCATGACAGGACCATTAGTGGCTGGAGGGCTCCTCACTGTGTGCTACGTGCTCACTGGACGATCGGCCGATTTGGAACTGGAGAGAGCCGCCGATGTCAAATGGGAAGATCAGGCAGAGATATCAGGAAGCAGTCCAATCCTGTCAATAACAATATCAGAAGATGGTAGCATGTCGATAAAAAACGAAGAGGAAGAACAAACACTGACCATACTCATTAGAACAGGATTGCTGGTGATCTCAGGACTTTTTCCTGTATCAATACCAATCACGGCAGCAGCATGGTACCTGTGGGAAGTGAAGAAACAACGGGCTGGAGTATTGTGGGATGTCCCTTCACCCCCACCCGTGGGAAAGGCTGAACTGGAAGATGGAGCCTATAGAATCAAGCAAAAAGGGATTCTTGGATATTCCCAGATCGGAGCCGGAGTTTACAAAGAAGGAACATTCCATACAATGTGGCATGTCACACGCGGCGCTGTTCTAATGCATAAAGGAAAGAGGATTGAACCATCATGGGCGGACGTTAAGAAAGACCTAATATCATATGGAGGAGGCTGGAAGCTAGAAGGAGAATGGAAGGAAGGAGAAGAAGTCCAGGTCTTGGCATTGGAGCCTGGAAAAAATCCAAGAGCCGTCCAAACAAAACCTGGTCTTTTCAAAACCAACGCCGGAACCATAGGTGCCGTATCTCTGGACTTTTCTCCTGGAACCTCAGGATCTCCAATCATCGACAAAAAAGGAAAAGTTGTGGGTCTTTATGGTAATGGTGTTGTTACAAGGAGTGGAGCATATGTGAGTGCTATAGCCCAGACTGAAAAAAGTATTGAAGACAATCCAGAGATCGAAGATGACATTTTTCGAAAGAGAAAATTGACCATCATGGACCTCCACCCAGGAGCGGGAAAGACGAAGAGATACCTTCCGGCCATAGTCAGAGAGGCTATAAAACGGGGCCTGAGGACATTAATCCTGGCCCCCACTAGAGTCGTGGCAGCTGAAATGGAGGAAGCCCTAAGAGGACTTCCAATAAGATACCAAACCCCAGCCATCAGAGCTGAGCACACCGGGCGGGAGATTGTGGACCTAATGTGTCATGCCACATTCACTATGAGGCTGCTATCACCAGTTAGAGTGCCAAATTACAACCTGATCATCATGGACGAAGCCCATTTCACAGACCCAGCAAGTATAGCGGCTAGAGGATACATCTCAACTCGAGTAGAGATGGGTGAGGCAGCTGGGATTTTCATGACAGCCACTCCTCCGGGAAGCAGAGACCCATTCCCTCAGAGCAATGCACCAATCATGGATGAAGAAAGAGAAATCCCTGAACGTTCGTGGAGTTCTGGACATGAGTGGGTCACGGATTTTAAAGGGAAGACTGTTTGGTTCGTTCCAAGTATAAAAGCAGGAAATGATATAGCAGCTTGCCTGAGAAAAAATGGAAAGAAAGTGATACAACTCAGTAGGAAGACCTTTGATTCTGAGTATGTCAAGACTAGAACCAATGATTGGGACTTCGTGGTCACAACTGACATTTCAGAAATGGGTGCCAACTTCAAGGCTGAGAGGGTTATAGACCCCAGACGCTGCATGAAACCAGTTATACTAACAGATGGTGAAGAGCGGGTGATCCTGGCAGGACCTATGCCAGTGACCCACTCTAGTGCAGCACAAAGAAGAGGGAGAATAGGAAGAAATCCAAAAAATGAAAATGACCAGTACATATACATGGGGGAACCTCTGGAAAATGATGAAGACTGTGCACACTGGAAAGAAGCTAAAATGCTCCTAGATAACATCAACACACCTGAAGGAATCATTCCTAGCATGTTCGAACCAGAGCGTGAAAAGGTGGATGCCATTGATGGTGAATACCGCTTGAGAGGAGAAGCAAGGAAAACCTTTGTGGACCTAATGAGAAGAGGAGACCTACCAGTCTGGTTGGCCTACAGAGTGGCAGCTGAAGGCATCAACTACGCAGACAGAAGGTGGTGTTTTGATGGAATTAAGAACAACCAAATCTTGGAAGAAAATGTGGAGGTGGAAATCTGGACAAAAGAAGGGGAAAGGAAGAAATTAAAACCCAGATGGTTGGATGCCAGGATCTACTCTGACCCACTGGCGCTAAAGGAATTCAAGGAGTTTGCAGCTGGAAGAAAGTCCCTGACCCTGAACCTAATCACAGAAATGGGTAGGCTTCCAACTTTCATGACTCAGAAGGCAAGAGACGCACTGGACAACTTAGCAGTGCTGCACACGGCTGAAGCAGGTGGAAGGGCGTACAATCATGCTCTCAGTGAACTGCCGGAGACCCTGGAGACATTGCTTTTACTGACACTTCTGGCTACAGTCACAGGAGGAATCTTTTTATTCTTGATGAGCGGAAGGGGTATAGGGAAGATGACCCTGGGAATGTGCTGCATAATCACGGCTAGTATTCTCCTATGGTACGCACAAATACAGCCACACTGGATAGCAGCTTCAATAATACTGGAGTTTTTTCTCATAGTTTTGCTTATTCCAGAACCAGAAAAGCAGAGAACACCCCAAGATAACCAATTGACCTACGTTGTCATAGCCATCCTCACAGTGGTGGCCGCAACCATGGCAAACGAGATGGGTTTCCTGGAAAAAACGAAGAAAGATCTCGGATTGGGAAGCATTACAACCCAGCAACCCGAGAGCAACATCCTGGACATAGATCTACGTCCCGCATCAGCATGGACGCTGTATGCTGTGGCCACAACATTTGTCACACCAATGTTGAGACACAGCATTGAAAATTCCTCAGTGAACGTGTCCCTAACAGCTATTGCCAACCAAGCCACAGTGTTAATGGGTCTTGGGAAAGGATGGCCATTGTCAAAGATGGACATCGGAGTTCCCCTTCTCGCCATTGGATGCTACTCACAAGTCAACCCCATAACTCTCACAGCAGCTCTTTTCTTACTGGTAGCACATTATGCCATCATAGGGCCAGGACTCCAAGCAAAAGCAACCAGAGAAGCTCAGAAAAGAGCAGCAGCGGGCATCATGAAAAACCCAACTGTCGATGGAATAACAGTGATTGACCTAGATCCAATACCCTATGATCCAAAGTTTGAAAAGCAGTTGGGACAAGTAATGCTCCTAGTCCTCTGCGTGACTCAAGTGTTGATGATGAGGACTACATGGGCTCTGTGTGAGGCTTTAACCTTAGCGACCGGGCCTATCTCCACATTGTGGGAAGGAAATCCAGGGAGGTTTTGGAACACTACCATTGCAGTGTCAATGGCTAACATTTTTAGAGGGAGTTACTTGGCCGGAGCTGGACTTCTCTTTTCCATCATGAAGAACACAACCAACACGAGAAGGGGAACTGGCAACATAGGAGAGACGCTTGGAGAGAAATGGAAAAGCCGATTGAACGCATTGGGGAAAAGTGAATTCCAGATCTACAAGAAAAGTGGAATCCAGGAAGTGGATAGAACCTTAGCAAAAGAAGGCATTAAAAGAGGAGAAACGGACCATCACGCTGTGTCGCGAGGCTCAGCAAAACTGAGATGGTTCGTCGAGAGAAATATGGTCACACCAGAAGGGAAAGTAGTGGACCTCGGTTGCGGCAGAGGAGGCTGGTCATACTATTGTGGGGGACTAAAGAATGTAAGAGAAGTCAAAGGCCTAACAAAAGGAGGACCAGGACATGAAGAACCCATCCCCATGTCAACATATGGGTGGAATCTAGTACGTCTTCAAAGTGGAGTTGACGTTTTCTTCACTCCGCCAGAAAAGTGTGACACATTGTTGTGTGACATAGGGGAGTCGTCACCAAATCCCACGGTAGAAGCAGGACGAACACTCAGAGTCCTTAACTTAGTGGAAAATTGGTTGAACAACAACACCCAATTTTGCATAAAGGTTCTCAACCCATACATGCCCTCAGTCATAGAAAAAATGGAAGCACTACAAAGGAAATATGGAGGAGCCTTAGTGAGGAATCCACTCTCACGAAACTCCACACATGAGATGTACTGGGTATCCAATGCCTCCGGGAACATAGTGTCATCAGTGAACATGATTTCAAGGATGTTGATCAACAGATTCACAATGAGACACAAGAAAGCCACTTACGAGCCAGATGTAGACCTCGGAAGCGGAACCCGCAACATCGGAATTGAAAGTGAGATACCAAACCTAGACATAATCGGGAAAAGAATAGAAAAAATAAAACAAGAGCATGAAACATCATGGCACTATGACCAAGACCACCCATACAAAACGTGGGCTTACCATGGCAGCTATGAAACAAAACAAACTGGATCAGCATCATCCATGGTGAACGGAGTGGTCAGACTGCTGACAAAACCTTGGGACGTCGTCCCCATGGTGACACAGATGGCAATGACAGACACGACTCCATTTGGACAACAGCGCGTTTTTAAAGAAAAAGTGGACACGAGAACCCAAGAACCGAAAGAAGGCACAAAGAAACTAATGAAAATCACGGCAGAGTGGCTTTGGAAAGAACTAGGGAAGAAAAAGACACCTAGGATGTGCACTAGAGAAGAATTCACAAGAAAGGTGAGAAGCAATGCAGCCTTGGGGGCCATATTCACTGATGAGAACAAGTGGAAGTCGGCACGTGAGGCTGTTGAAGATAGTAGGTTTTGGGAGCTGGTTGACAAGGAAAGGAATCTCCATCTTGAAGGAAAGTGTGAAACATGTGTGTATAACATGATGGGAAAAAGAGAGAAGAAGCTAGGGGAGTTCGGCAAGGCAAAAGGCAGCAGAGCCATATGGTACATGTGGCTTGGAGCACGCTTCTTAGAGTTTGAAGCCCTAGGATTCTTGAATGAAGATCACTGGTTCTCCAGAGAGAACTCCTTGAGTGGAGTGGAAGGAGAAGGGCTGCACAAGCTAGGTTACATTTTAAGAGACGTGAGCAAGAAAGAGGGAGGAGCAATGTATGCCGATGACACCGCAGGATGGGACACAAGAATCACACTAGAAGACCTAAAAAATGAAGAAATGGTAACAAACCACATGGAAGGAGAACACAAGAAACTAGCCGAGGCCATTTTCAAATTAACGTACCAAAACAAGGTGGTGCGTGTGCAAAGACCAACACCAAGAGGCACAGTAATGGATATCATATCGAGAAGAGACCAAAGAGGTAGTGGACAAGTTGGTACCTATGGACTCAATACTTTCACCAATATGGAAGCCCAACTAATCAGACAGATGGAGGGAGAAGGAGTCTTCAAAAGCATTCAGCACCTGACAGTCACAGAAGAAATCGCCGTGCAAAACTGGTTAGCAAGAGTAGGGCGCGAAAGGTTATCAAGAATGGCCATCAGTGGAGATGATTGTGTTGTGAAACCTTTAGATGACAGGTTCGCAAGCGCTTTAACAGCTCTAAATGACATGGGAAAGGTTAGGAAAGACATACAACAATGGGAACCTTCAAGAGGATGGAACGATTGGACACAAGTGCCCTTCTGTTCACACCATTTCCATGAGTTAATCATGAAAGACGGCCGCGTACTTGTAGTTCCATGCAGAAACCAAGATGAACTGATTGGTAGAGCCCGAATTTCCCAAGGAGCTGGGTGGTCTTTGCGAGAGACGGCCTGTTTGGGGAAGTCCTACGCCCAAATGTGGAGCTTGATGTACTTCCACAGACGTGACCTCAGGCTGGCGGCTAATGCTATTTGCTCGGCAGTCCCATCACATTGGGTTCCAACAAGTAGAACAACCTGGTCCATACACGCCAAACATGAATGGATGACAACGGAAGACATGCTGACAGTCTGGAACAGGGTGTGGATTCAAGAAAACCCATGGATGGAAGACAAAACTCCAGTGGAATCATGGGAGGAAATCCCATACTTGGGGAAAAGAGAAGACCAATGGTGCGGCTCATTGATTGGGCTAACAAGCAGGGCCACCTGGGCAAAGAACATCCAAACAGCAATAAATCAAGTTAGATCCCTTATAGGCTATGAGGAATACACAGATTACATGCCATCCATGAAAAGATTCAGAAGAGAAGAGGAAGAGGCAGGAGTCCTGTGGTAGAAGGCAAAACTAACATGAAACAAGGCTAGAAGTCAGGTCGGATTAAGCCATAGTACGGAAAAAACTATGCTACCTGTGAGCCCCGTCCAAGGACGTTAAAAGAAGTCAGGCCATTACAAATGCCATAGCTTGAGTAAACTGAGCAGCCTGTAGCTCCACCTGAGAAGGTGTAAAAAATCTGGGAGGCCACAAACCATGGAAGCTGTACGCATGGCGTAGTGGACTAGCGGTTAGAGGAGACCCCTCCCTTACAAATCGCAGCAACAATGGGGGCCCAAGGTGAGATGAAGCTGTAGTCTCACTGGAAGGACTAGAGGTTAGAGGAGACCCCCCCAAAACAAAAAACAGCATATTGACGCTGGGAAAGACCAGAGATCCTGCTGTCTCCTCAGCATCATTCCAGGCACAGAACGCCAGAAAATGGAATGGTGCTGTTGAATCAACAGGTTCT

**IRES-Mdm2-IRES-E73(DENV4)-DENV2**

AGTTGTTAGTCTACGTGGACCGACAAAGACAGATTCTTTGAGGGAGCTAAGCTCAACGTAGTTCTAACAGTTTTTTAATTAGAGAGCAGATCTCTGATGAATAACCAACGAAAAAAGGCGAGAAATACGCCTTTCAATATGCTGAAACGCGAGAGAAACCGCGTGTCGACTGTACAACAGCTGACAAAGAGATTCTCACTTGGAATGCTGCAGGGACGAGGACCATTAAAACTGTTCATGGCCCTGGTGGCGTTCCTTCGTTTCCTAACAATCCCACCAACAGCAGGGATACTGAAGAGATGGGGAACAATTAAAAAATCAAAAGCCATTAATGTTTTGAGAGGGTTCAGGAAAGAGATTGGAAGGATGCTGAACATCTTGAACAGGAGACGCAGAACTGCAGGCATGATCATTATGCTGATTCCAACAGTGATGGCGTTCCATTTAACCACACGTAACGGAGAACCACACATGATCGTCAGTAGACAAGAGAAAGGGAAAAGTCTTCTGTTTAAAACAGAGGATGGTGTGAACATGTGTACCCTCATGGCCATGGACCTTGGTGAATTGTGTGAAGATACAATCACGTACAAGTGTCCTTTTCTCAAGCAGAATGAACCAGAAGACATAGATTGTTGGTGCAACTCTACGTCCACATGGGTAACTTATGGGACGTGTACCACCACAGGAGAACACAGAAGAGAAAAAAGATCAGTGGCACTCGTTCCACATGTGGGAATGGGACTGGAGACACGAACTGAAACATGGATGTCATCAGAAGGGGCCTGGAAACATGCCCAGAGAATTGAAACTTGGATCTTGAGACATCCAGGCTTTACCATAATGGCAGCAATCCTGGCATACACCATAGGAACGACACATTTCCAAAGAGCCCTGATTTTCATCTTACTGACAGCTGTCGCTCCTTCAATGACAATGCGTTGCATAGGAATATCAAATAGAGACTTTGTAGAAGGGGTTTCAGGAGGAAGCTGGGTTGACATAGTCTTAGAACATGGAAGCTGTGTGACGACGATGGCAAAAAACAAACCAACATTGGATTTTGAACTGATAAAAACAGAAGCCAAACAACCTGCCACTCTAAGGAAGTACTGTATAGAGGCAAAGCTGACCAACACAACAACAGATTCTCGCTGCCCAACACAAGGAGAACCCAGCCTAAATGAAGAGCAGGACAAAAGGTTCGTCTGCAAACACTCCATGGTGGACAGAGGATGGGGAAATGGATGTGGACTATTTGGAAAAGGAGGCATTGTGACCTGTGCTATGTTCACATGCAAAAAGAACATGAAAGGAAAAGTCGTGCAACCAGAAAACTTGGAATACACCATTGTGATAACACCTCACTCAGGGGAAGAGCATGCAGTCGGAAATGACACAGGAAAACATGGCAAGGAAATCAAAATAACACCACAGAGTTCCATCACAGAAGCAGAGTTGACAGGCTATGGCACTGTCACGATGGAGTGCTCTCCGAGAACGGGCCTCGACTTCAATGAGATGGTGTTGCTGCAAATGGAAAATAAAGCTTGGCTGGTGCACAGGCAATGGTTCCTAGACCTGCCGTTGCCATGGCTGCCCGGAGCGGACACACAAGGATCAAATTGGATACAGAAAGAGACATTGGTCACTTTCAAAAATCCCCATGCGAAGAAACAGGATGTTGTTGTTTTGGGATCCCAAGAAGGGGCCATGCACACAGCACTCACAGGGGCCACAGAAATCCAGATGTCATCAGGAAACTTACTGTTCACAGGACATCTCAAGTGCAGGCTGAGGATGGACAAACTACAGCTCAAAGGAATGTCATACTCTATGTGCACAGGAAAGTTTAAAGTTGTGAAGGAAATAGCAGAAACACAACATGGAACAATAGTTATCAGAGTACAATATGGAGGGGACGGTTCTCCATGTAAGATCCCTTTTGAGATAATGGATTTGGAAAAAAGACATGTTTTAGGTCGCCTGATTACAGTCAACCCAATCGTAACAGAAAAAGATAGCCCAGTCAACATAGAAGCAGAACCTCCATTCGGAGACAGCTACATCATCATAGGAGTAGAGCCGGGACAATTGAAGCTCAACTGGTTTAAGAAAGGAAGTTCTATCGGCCAAATGATTGAGACAACAATGAGGGGAGCGAAGAGAATGGCCATTTTAGGTGACACAGCTTGGGATTTTGGATCCCTGGGAGGAGTGTTTACATCTATAGGAAAGGCTCTCCACCAAGTTTTCGGAGCAATCTATGGGGCTGCCTTCAGTGGGGTCTCATGGATTATGAAAATCCTCATAGGAGTCATTATCACATGGATAGGAATGAATTCACGCAGCACCTCACTGTCTGTGTCACTAGTATTGGTGGGAGTCGTGACGCTGTATTTGGGAGTTATGGTGCAGGCCTAGGCGGCCGCCCCTCTCCCTCCCCCCCCCCTAACGTTACTGGCCGAAGCCGCTTGGAATAAGGCCGGTGTGCGTTTGTCTATATGTTATTTTCCACCATATTGCCGTCTTTTGGCAATGTGAGGGCCCGGAAACCTGGCCCTGTCTTCTTGACGAGCATTCCTAGGGGTCTTTCCCCTCTCGCCAAAGGAATGCAAGGTCTGTTGAATGTCGTGAAGGAAGCAGTTCCTCTGGAAGCTTCTTGAAGACAAACAACGTCTGTAGCGACCCTTTGCAGGCAGCGGAACCCCCCACCTGGCGACAGGTGCCTCTGCGGCCAAAAGCCACGTGTATAAGATACACCTGCAAAGGCGGCACAACCCCAGTGCCACGTTGTGAGTTGGATAGTTGTGGAAAGAGTCAAATGGCTCTCCTCAAGCGTATTCAACAAGGGGCTGAAGGATGCCCAGAAGGTACCCCATTGTATGGGATCTGATCTGGGGCCTCGGTGCACATGCTTTACATGTGTTTAGTCGAGGTTAAAAAAACGTCTAGGCCCCCCGAACCACGGGGACGTGGTTTTCCTTTGAAAAACACGATAATACCATGGTGAGGAGCAGGCAAATGTGCAATACCAACATGTCTGTACCTACTGATGGTGCTGTAACCACCTCACAGATTCCAGCTTCGGAACAAGAGACCCTGGTTAGACCAAAGCCATTGCTTTTGAAGTTATTAAAGTCTGTTGGTGCACAAAAAGACACTTATACTATGAAAGAGAATCATCGGACTCAGGTACATCTGTGAGTGAGAACAGGTGTCACCTTGAAGGTGGGAGTGATCAAAAGGACCTTGTACAAGAGCTTCAGGAAGAGAAACCTTCATCTTCACATTTGGTTTCTAGACCATCTACCTCATCTAGAAGGAGAGCAATTAGTGAGACAGAAGAAAATTCAGATGAATTATCTGGTGAACGACAAAGAAAACGCCACAAATCTGATAGTATTTCCCTTTCCTTTGATGAAAGCCTGGCTCTGTGTGTAATAAGGGAGATATGTTGTGAAAGAAGCAGTAGCAGTGAATCTACAGGGACGCCATCGAATCCGGATCTTGATGCTGGTGTAAGTGAACATTCAGGTGATTGGTTGGATCAGGATTCAGTTTCAGATCAGTTTAGTGTAGAATTTGAAGTTGAATCTCTCGACTCAGAAGATTATAGCCTTAGTGAAGAAGGACAAGAACTCTCAGATGAAGATGATGAGGTATATCAAGTTACTGTGTATCAGGCAGGGGAGAGTGATACAGATTCATTTGAAGAAGATCCTGAAATTTCCTTAGCTGACTATTGGAAATGCACTTCATGCAATGAAATGAATCCCCCCCTTCCATCACATTGCAACAGATGTTGGGCCCTTCGTGAGAATTGGCTTCCTGAAGATAAAGGGAAAGATAAAGGGGAAATCTCTGAGAAAGCCAAACTGGAAAACTCAACACAAGCTGAAGAGGGCTTTGATGTTCCTGATTGTAAAAAAACTATAGTGAATGATTCCAGAGAGTCATGTGTTGAGGAAAATGATGATAAAATTACACAAGCTTCACAATCACAAGAAAGTGAAGACTATTCTCAGCCATCAACTTCTAGTAGCATTATTTATAGCAGCCAAGAAGATGTGAAAGAGTTTGAAAGGGAAGAAACCCAAGACAAAGAAGAGAGTGTGGAATCTAGTTTGCCCCTTAATGCCATTGAACCTTGTGTGATTTGTCAAGGTCGACCTAAAAATGGTTGCATTGTCCATGGCAAAACAGGACATCTTATGGCCTGCTTTACATGTGCAAAGAAGCTAAAGAAAAGGAATAAGCCCTGCCCAGTATGTAGACAACCAATTCAAATGATTGTGCTAACTTATTTCCCCTAGGCGGCCGCCCCTCTCCCTCCCCCCCCCCCTAACGTTACTGGCCGAAGCCGCTTGGAATAAGGCCGGTGTGCGTTTGTCTATATGTTATTTTCCACCATATTGCCGTCTTTTGGCAATGTGAGGGCCCGGAAACCTGGCCCTGTCTTCTTGACGAGCATTCCTAGGGGTCTTTCCCCTCTCGCCAAAGGAATGCAAGGTCTGTTGAATGTCGTGAAGGAAGCAGTTCCTCTGGAAGCTTCTTGAAGACAAACAACGTCTGTAGCGACCCTTTGCAGGCAGCGGAACCCCCCACCTGGCGACAGGTGCCTCTGCGGCCAAAAGCCACGTGTATAAGATACACCTGCAAAGGCGGCACAACCCCAGTGCCACGTTGTGAGTTGGATAGTTGTGGAAAGAGTCAAATGGCTCTCCTCAAGCGTATTCAACAAGGGGCTGAAGGATGCCCAGAAGGTACCCCATTGTATGGGATCTGATCTGGGGCCTCGGTGCACATGCTTTACATGTGTTTAGTCGAGGTTAAAAAAACGTCTAGGCCCCCCGAACCACGGGGACGTGGTTTTCCTTTGAAAAACACGATAATACCATGGGTTCCGTTGGTGGACTGTTCACATCATTGGGAAAGGCTGTGCACCAGGTTTTTGGAAGTGTGTATACAACCATGTTTGGAGGAGTCTCATGGATGATTAGAATCCTAATTGGGTTCTTAGTGTTGTGGATTGGCACGAACTCAAGGAACACTTCAATGGCTATGACGTGCATAGCTGTTGGAGGAATCACTCTGTTTCTGGGCTTCACAGTTCAAGCAGATAGTGGTTGCGTTGTGAGCTGGAAAAACAAAGAACTGAAGTGTGGCAGTGGGATTTTCATCACAGACAACGTGCACACATGGACAGAACAATACAAGTTCCAACCAGAATCCCCTTCAAAACTAGCTTCAGCTATCCAGAAAGCTCATGAAGAGGGCATTTGTGGAATCCGCTCAGTAACAAGACTGGAAAATCTGATGTGGAAACAAATAACACCAGAATTGAATCACATTCTATCAGAAAATGAGGTGAAGTTGACTATTATGACAGGAGACATCAAAGGAATCATGCAGGCAGGAAAACGATCTCTGCAGCCCCAGCCCACTGAGCTGAAGTATTCATGGAAAACATGGGGCAAAGCGAAAATGCTCTCTACAGAGTCTCATAACCAGACCTTTCTCATTGATGGCCCCGAAACAGCAGAATGCCCCAACACAAACAGAGCTTGGAATTCGCTGGAAGTTGAAGACTATGGCTTTGGAGTATTCACCACCAATATATGGCTAAAGTTGAGAGAAAAGCAGGATGTATTCTGCGACTCAAAACTCATGTCAGCGGCCATAAAAGACAACAGAGCCGTCCATGCCGATATGGGTTATTGGATAGAAAGTGCACTCAATGACACATGGAAGATAGAGAAAGCCTCTTTCATCGAAGTTAAAAGCTGCCACTGGCCAAAGTCACACACCCTCTGGAGTAATGGAGTGTTAGAAAGTGAGATGATAATTCCAAAGAATTTCGCTGGACCAGTGTCACAACACAACTACAGACCAGGTTACCATACACAAACAGCAGGACCATGGCATCTAGGTAAGCTTGAGATGGACTTTGATTTCTGCGAAGGAACCACAGTGGTGGTGACTGAGGACTGTGGAAATAGAGGACCCTCTTTAAGAACAACTACTGCCTCTGGAAAACTCATAACAGAATGGTGCTGCCGATCTTGCACATTACCACCGCTAAGATACAGAGGTGAGGACGGATGCTGGTACGGGATGGAAATCAGACCATTGAAAGAGAAAGAAGAGAATTTGGTCAACTCCTTGGTCACAGCCGGACATGGGCAGATTGACAACTTTTCACTAGGAGTCTTGGGAATGGCATTGTTCCTGGAAGAAATGCTCAGGACCCGAGTAGGAACGAAACATGCAATACTACTAGTTGCAGTTTCTTTTGTGACATTGATCACAGGGAACATGTCCTTTAGAGACCTGGGAAGAGTGATGGTTATGGTGGGCGCTACTATGACGGATGACATAGGTATGGGCGTGACTTATCTTGCCCTACTAGCAGCCTTCAAAGTCAGACCAACTTTTGCAGCCGGACTACTCTTGAGAAAGTTGACCTCCAAGGAATTGATGATGACTACCATAGGAATCGTACTCCTCTCCCAGAGCACCATACCAGAGACCATTCTTGAACTGACTGATGCGTTAGCCTTGGGCATGATGGTCCTTAAAATGGTGAGAAAAATGGAAAAGTATCAATTGGCAGTGACTATCATGGCTATCTTGTGCGTCCCAAATGCAGTGATATTACAAAACGCATGGAAAGTGAGTTGCACAATATTGGCAGTGGTGTCCGTTTCCCCACTGTTCTTAACATCCTCACAGCAGAAAGCGGATTGGATACCATTAGCATTGACGATCAAGGGTCTCAATCCAACAGCTATTTTTCTAACAACCCTTTCAAGAACCAACAAGAAAAGGAGCTGGCCACTAAATGAGGCTATCATGGCAGTCGGGATGGTGAGCATTTTGGCCAGTTCACTCCTAAAGAATGACATTCCCATGACAGGACCATTAGTGGCTGGAGGGCTCCTCACTGTGTGCTACGTGCTCACTGGACGATCGGCCGATTTGGAACTGGAGAGAGCCGCCGATGTCAAATGGGAAGATCAGGCAGAGATATCAGGAAGCAGTCCAATCCTGTCAATAACAATATCAGAAGATGGTAGCATGTCGATAAAAAACGAAGAGGAAGAACAAACACTGACCATACTCATTAGAACAGGATTGCTGGTGATCTCAGGACTTTTTCCTGTATCAATACCAATCACGGCAGCAGCATGGTACCTGTGGGAAGTGAAGAAACAACGGGCTGGAGTATTGTGGGATGTCCCTTCACCCCCACCCGTGGGAAAGGCTGAACTGGAAGATGGAGCCTATAGAATCAAGCAAAAAGGGATTCTTGGATATTCCCAGATCGGAGCCGGAGTTTACAAAGAAGGAACATTCCATACAATGTGGCATGTCACACGCGGCGCTGTTCTAATGCATAAAGGAAAGAGGATTGAACCATCATGGGCGGACGTTAAGAAAGACCTAATATCATATGGAGGAGGCTGGAAGCTAGAAGGAGAATGGAAGGAAGGAGAAGAAGTCCAGGTCTTGGCATTGGAGCCTGGAAAAAATCCAAGAGCCGTCCAAACAAAACCTGGTCTTTTCAAAACCAACGCCGGAACCATAGGTGCCGTATCTCTGGACTTTTCTCCTGGAACCTCAGGATCTCCAATCATCGACAAAAAAGGAAAAGTTGTGGGTCTTTATGGTAATGGTGTTGTTACAAGGAGTGGAGCATATGTGAGTGCTATAGCCCAGACTGAAAAAAGTATTGAAGACAATCCAGAGATCGAAGATGACATTTTTCGAAAGAGAAAATTGACCATCATGGACCTCCACCCAGGAGCGGGAAAGACGAAGAGATACCTTCCGGCCATAGTCAGAGAGGCTATAAAACGGGGCCTGAGGACATTAATCCTGGCCCCCACTAGAGTCGTGGCAGCTGAAATGGAGGAAGCCCTAAGAGGACTTCCAATAAGATACCAAACCCCAGCCATCAGAGCTGAGCACACCGGGCGGGAGATTGTGGACCTAATGTGTCATGCCACATTCACTATGAGGCTGCTATCACCAGTTAGAGTGCCAAATTACAACCTGATCATCATGGACGAAGCCCATTTCACAGACCCAGCAAGTATAGCGGCTAGAGGATACATCTCAACTCGAGTAGAGATGGGTGAGGCAGCTGGGATTTTCATGACAGCCACTCCTCCGGGAAGCAGAGACCCATTCCCTCAGAGCAATGCACCAATCATGGATGAAGAAAGAGAAATCCCTGAACGTTCGTGGAGTTCTGGACATGAGTGGGTCACGGATTTTAAAGGGAAGACTGTTTGGTTCGTTCCAAGTATAAAAGCAGGAAATGATATAGCAGCTTGCCTGAGAAAAAATGGAAAGAAAGTGATACAACTCAGTAGGAAGACCTTTGATTCTGAGTATGTCAAGACTAGAACCAATGATTGGGACTTCGTGGTCACAACTGACATTTCAGAAATGGGTGCCAACTTCAAGGCTGAGAGGGTTATAGACCCCAGACGCTGCATGAAACCAGTTATACTAACAGATGGTGAAGAGCGGGTGATCCTGGCAGGACCTATGCCAGTGACCCACTCTAGTGCAGCACAAAGAAGAGGGAGAATAGGAAGAAATCCAAAAAATGAAAATGACCAGTACATATACATGGGGGAACCTCTGGAAAATGATGAAGACTGTGCACACTGGAAAGAAGCTAAAATGCTCCTAGATAACATCAACACACCTGAAGGAATCATTCCTAGCATGTTCGAACCAGAGCGTGAAAAGGTGGATGCCATTGATGGTGAATACCGCTTGAGAGGAGAAGCAAGGAAAACCTTTGTGGACCTAATGAGAAGAGGAGACCTACCAGTCTGGTTGGCCTACAGAGTGGCAGCTGAAGGCATCAACTACGCAGACAGAAGGTGGTGTTTTGATGGAATTAAGAACAACCAAATCTTGGAAGAAAATGTGGAGGTGGAAATCTGGACAAAAGAAGGGGAAAGGAAGAAATTAAAACCCAGATGGTTGGATGCCAGGATCTACTCTGACCCACTGGCGCTAAAGGAATTCAAGGAGTTTGCAGCTGGAAGAAAGTCCCTGACCCTGAACCTAATCACAGAAATGGGTAGGCTTCCAACTTTCATGACTCAGAAGGCAAGAGACGCACTGGACAACTTAGCAGTGCTGCACACGGCTGAAGCAGGTGGAAGGGCGTACAATCATGCTCTCAGTGAACTGCCGGAGACCCTGGAGACATTGCTTTTACTGACACTTCTGGCTACAGTCACAGGAGGAATCTTTTTATTCTTGATGAGCGGAAGGGGTATAGGGAAGATGACCCTGGGAATGTGCTGCATAATCACGGCTAGTATTCTCCTATGGTACGCACAAATACAGCCACACTGGATAGCAGCTTCAATAATACTGGAGTTTTTTCTCATAGTTTTGCTTATTCCAGAACCAGAAAAGCAGAGAACACCCCAAGATAACCAATTGACCTACGTTGTCATAGCCATCCTCACAGTGGTGGCCGCAACCATGGCAAACGAGATGGGTTTCCTGGAAAAAACGAAGAAAGATCTCGGATTGGGAAGCATTACAACCCAGCAACCCGAGAGCAACATCCTGGACATAGATCTACGTCCCGCATCAGCATGGACGCTGTATGCTGTGGCCACAACATTTGTCACACCAATGTTGAGACACAGCATTGAAAATTCCTCAGTGAACGTGTCCCTAACAGCTATTGCCAACCAAGCCACAGTGTTAATGGGTCTTGGGAAAGGATGGCCATTGTCAAAGATGGACATCGGAGTTCCCCTTCTCGCCATTGGATGCTACTCACAAGTCAACCCCATAACTCTCACAGCAGCTCTTTTCTTACTGGTAGCACATTATGCCATCATAGGGCCAGGACTCCAAGCAAAAGCAACCAGAGAAGCTCAGAAAAGAGCAGCAGCGGGCATCATGAAAAACCCAACTGTCGATGGAATAACAGTGATTGACCTAGATCCAATACCCTATGATCCAAAGTTTGAAAAGCAGTTGGGACAAGTAATGCTCCTAGTCCTCTGCGTGACTCAAGTGTTGATGATGAGGACTACATGGGCTCTGTGTGAGGCTTTAACCTTAGCGACCGGGCCTATCTCCACATTGTGGGAAGGAAATCCAGGGAGGTTTTGGAACACTACCATTGCAGTGTCAATGGCTAACATTTTTAGAGGGAGTTACTTGGCCGGAGCTGGACTTCTCTTTTCCATCATGAAGAACACAACCAACACGAGAAGGGGAACTGGCAACATAGGAGAGACGCTTGGAGAGAAATGGAAAAGCCGATTGAACGCATTGGGGAAAAGTGAATTCCAGATCTACAAGAAAAGTGGAATCCAGGAAGTGGATAGAACCTTAGCAAAAGAAGGCATTAAAAGAGGAGAAACGGACCATCACGCTGTGTCGCGAGGCTCAGCAAAACTGAGATGGTTCGTCGAGAGAAATATGGTCACACCAGAAGGGAAAGTAGTGGACCTCGGTTGCGGCAGAGGAGGCTGGTCATACTATTGTGGGGGACTAAAGAATGTAAGAGAAGTCAAAGGCCTAACAAAAGGAGGACCAGGACATGAAGAACCCATCCCCATGTCAACATATGGGTGGAATCTAGTACGTCTTCAAAGTGGAGTTGACGTTTTCTTCACTCCGCCAGAAAAGTGTGACACATTGTTGTGTGACATAGGGGAGTCGTCACCAAATCCCACGGTAGAAGCAGGACGAACACTCAGAGTCCTTAACTTAGTGGAAAATTGGTTGAACAACAACACCCAATTTTGCATAAAGGTTCTCAACCCATACATGCCCTCAGTCATAGAAAAAATGGAAGCACTACAAAGGAAATATGGAGGAGCCTTAGTGAGGAATCCACTCTCACGAAACTCCACACATGAGATGTACTGGGTATCCAATGCCTCCGGGAACATAGTGTCATCAGTGAACATGATTTCAAGGATGTTGATCAACAGATTCACAATGAGACACAAGAAAGCCACTTACGAGCCAGATGTAGACCTCGGAAGCGGAACCCGCAACATCGGAATTGAAAGTGAGATACCAAACCTAGACATAATCGGGAAAAGAATAGAAAAAATAAAACAAGAGCATGAAACATCATGGCACTATGACCAAGACCACCCATACAAAACGTGGGCTTACCATGGCAGCTATGAAACAAAACAAACTGGATCAGCATCATCCATGGTGAACGGAGTGGTCAGACTGCTGACAAAACCTTGGGACGTCGTCCCCATGGTGACACAGATGGCAATGACAGACACGACTCCATTTGGACAACAGCGCGTTTTTAAAGAAAAAGTGGACACGAGAACCCAAGAACCGAAAGAAGGCACAAAGAAACTAATGAAAATCACGGCAGAGTGGCTTTGGAAAGAACTAGGGAAGAAAAAGACACCTAGGATGTGCACTAGAGAAGAATTCACAAGAAAGGTGAGAAGCAATGCAGCCTTGGGGGCCATATTCACTGATGAGAACAAGTGGAAGTCGGCACGTGAGGCTGTTGAAGATAGTAGGTTTTGGGAGCTGGTTGACAAGGAAAGGAATCTCCATCTTGAAGGAAAGTGTGAAACATGTGTGTATAACATGATGGGAAAAAGAGAGAAGAAGCTAGGGGAGTTCGGCAAGGCAAAAGGCAGCAGAGCCATATGGTACATGTGGCTTGGAGCACGCTTCTTAGAGTTTGAAGCCCTAGGATTCTTGAATGAAGATCACTGGTTCTCCAGAGAGAACTCCTTGAGTGGAGTGGAAGGAGAAGGGCTGCACAAGCTAGGTTACATTTTAAGAGACGTGAGCAAGAAAGAGGGAGGAGCAATGTATGCCGATGACACCGCAGGATGGGACACAAGAATCACACTAGAAGACCTAAAAAATGAAGAAATGGTAACAAACCACATGGAAGGAGAACACAAGAAACTAGCCGAGGCCATTTTCAAATTAACGTACCAAAACAAGGTGGTGCGTGTGCAAAGACCAACACCAAGAGGCACAGTAATGGATATCATATCGAGAAGAGACCAAAGAGGTAGTGGACAAGTTGGTACCTATGGACTCAATACTTTCACCAATATGGAAGCCCAACTAATCAGACAGATGGAGGGAGAAGGAGTCTTCAAAAGCATTCAGCACCTGACAGTCACAGAAGAAATCGCCGTGCAAAACTGGTTAGCAAGAGTAGGGCGCGAAAGGTTATCAAGAATGGCCATCAGTGGAGATGATTGTGTTGTGAAACCTTTAGATGACAGGTTCGCAAGCGCTTTAACAGCTCTAAATGACATGGGAAAGGTTAGGAAAGACATACAACAATGGGAACCTTCAAGAGGATGGAACGATTGGACACAAGTGCCCTTCTGTTCACACCATTTCCATGAGTTAATCATGAAAGACGGCCGCGTACTTGTAGTTCCATGCAGAAACCAAGATGAACTGATTGGTAGAGCCCGAATTTCCCAAGGAGCTGGGTGGTCTTTGCGAGAGACGGCCTGTTTGGGGAAGTCCTACGCCCAAATGTGGAGCTTGATGTACTTCCACAGACGTGACCTCAGGCTGGCGGCTAATGCTATTTGCTCGGCAGTCCCATCACATTGGGTTCCAACAAGTAGAACAACCTGGTCCATACACGCCAAACATGAATGGATGACAACGGAAGACATGCTGACAGTCTGGAACAGGGTGTGGATTCAAGAAAACCCATGGATGGAAGACAAAACTCCAGTGGAATCATGGGAGGAAATCCCATACTTGGGGAAAAGAGAAGACCAATGGTGCGGCTCATTGATTGGGCTAACAAGCAGGGCCACCTGGGCAAAGAACATCCAAACAGCAATAAATCAAGTTAGATCCCTTATAGGCTATGAGGAATACACAGATTACATGCCATCCATGAAAAGATTCAGAAGAGAAGAGGAAGAGGCAGGAGTCCTGTGGTAGAAGGCAAAACTAACATGAAACAAGGCTAGAAGTCAGGTCGGATTAAGCCATAGTACGGAAAAAACTATGCTACCTGTGAGCCCCGTCCAAGGACGTTAAAAGAAGTCAGGCCATTACAAATGCCATAGCTTGAGTAAACTGAGCAGCCTGTAGCTCCACCTGAGAAGGTGTAAAAAATCTGGGAGGCCACAAACCATGGAAGCTGTACGCATGGCGTAGTGGACTAGCGGTTAGAGGAGACCCCTCCCTTACAAATCGCAGCAACAATGGGGGCCCAAGGTGAGATGAAGCTGTAGTCTCACTGGAAGGACTAGAGGTTAGAGGAGACCCCCCCAAAACAAAAAACAGCATATTGACGCTGGGAAAGACCAGAGATCCTGCTGTCTCCTCAGCATCATTCCAGGCACAGAACGCCAGAAAATGGAATGGTGCTGTTGAATCAACAGGTTCT
